# Homologues of bacterial TnpB_*IS605* are widespread in diverse eukaryotic transposable elements

**DOI:** 10.1186/1759-8753-4-12

**Published:** 2013-04-01

**Authors:** Weidong Bao, Jerzy Jurka

**Affiliations:** 1 Genetic Information Research Institute, 1925 Landings Drive, Mountain View, CA, 94043, USA

**Keywords:** DNA transposon, TnpB, Fanzor, *Helitron*, *Helitron2*, *IS200/605*, *IS607*, Methyltransferase

## Abstract

**Background:**

Bacterial insertion sequences (IS) of *IS200*/*IS605* and *IS607* family often encode a transposase (TnpA) and a protein of unknown function, TnpB.

**Results:**

Here we report two groups of TnpB-like proteins (Fanzor1 and Fanzor2) that are widespread in diverse eukaryotic transposable elements (TEs), and in large double-stranded DNA (dsDNA) viruses infecting eukaryotes. Fanzor and TnpB proteins share the same conserved amino acid motif in their C-terminal half regions: D-X(125, 275)-[TS]-[TS]-X-X-[C4 zinc finger]-X(5,50)-RD, but are highly variable in their N-terminal regions. Fanzor1 proteins are frequently captured by DNA transposons from different superfamilies including *Helitron*, *Mariner*, *IS4*-like, *Sola* and *MuDr*. In contrast, Fanzor2 proteins appear only in some *IS607*-type elements. We also analyze a new *Helitron2* group from the *Helitron* superfamily, which contains elements with hairpin structures on both ends. Non-autonomous *Helitron2* elements (*CRe*-*1*, *2*, *3*) in the genome of green alga *Chlamydomonas reinhardtii* are flanked by target site duplications (TSDs) of variable length (approximately 7 to 19 bp).

**Conclusions:**

The phylogeny and distribution of the TnpB/Fanzor proteins indicate that they may be disseminated among eukaryotic species by viruses. We hypothesize that TnpB/Fanzor proteins may act as methyltransferases.

## Background

Transposable elements (TEs) are DNA segments that are duplicated and inserted into genomic DNA by a variety of mechanisms. There are two major groups of TEs: DNA transposons and retrotransposons. Retrotranposons are further divided into those containing long terminal repeats (LTRs), or LTR retrotransposons, and non-LTR retrotransposons, which are not flanked by LTRs. Typically, TEs encode only proteins essential for their reproduction and insertion, including reverse transcriptases and transposases (Tpases). Currently, there are four known types of transposases encoded by TEs. The most common type is the DDE-transposase encoded by most bacterial insertion sequences (IS), eukaryotic DNA transposons, and LTR retrotransposons. The second group is represented by reverse transcriptases (RT), encoded by a variety of non-LTR and LTR-retrotransposons. The third group includes tyrosine recombinases (YR) encoded by *IS91*[1], *Helitron*[2], *IS200*/*IS605*[1], *Crypton*[3], and *DIRS*-retrotransposon families [4,5]. The last group is represented by serine recombinases (SR), encoded by *IS607* family, *Tn4451*, and bacteriophage phiC31 [6]. The structural features and specific transposition mechanisms differ fundamentally among these TE groups. Most DNA transposons are flanked by terminal inverted repeats (TIRs) and target site duplications (TSDs), and are transposed by the ‘cut-and-paste’ mechanism used by DDE transposases, although some use replicative mechanism (Tn3) [7], or are able to switch to replicative mode (for example, *MuDr*, *Tn7* and *IS903*[8-11]). LTR-retrotransposons use RT and integrase (DDE-transposase) to complete their transposition. Non-LTR retrotransposons need both RT and endonuclease (EN) in their transposition process termed target site-primed reverse transcription (TPRT) [12]. Transposons using YR and SR as Tpase lack TIRs and produce no TSDs upon insertion. However, their terminal hairpin structures (*IS200*/*605* family) or terminal short direct repeats (*Crypton*) are important for transposition [3,13,14].

Elements from the *IS200*/*IS605* and *IS607* families usually encode a secondary protein (TnpB) of unknown function, in addition to transposase (TnpA). Three independent experiments on *IS607*, *ISHp608*, and *ISDra2* elements (the latter two belong to the *IS200*/*IS605* family), have shown that TnpB is dispensable for the transposition in *Escherichia coli*[14,15] and *Deinococcus radiodurans*[16]. Interestingly, numerous IS elements (for example, *IS1341*, *IS809* and *IS1136*) encode TnpB as the only protein (putative transposase), but the supporting evidence for TnpB-mediated transposition is still missing. Like other elements from the *IS200*/*IS605* and *IS607* families, these TnpB-only transposons lack TIRs and TSDs. One possibility is that these elements represent non-autonomous derivatives of *IS607* or *IS200*/*IS605*-like transposons, where TnpA is deleted. Due to this uncertainty, most of the TnpB-only elements are ambiguously assigned to the *IS200*/*IS605* family in the ISfinder database (http://www-is.biotoul.fr) [17].

In this paper, we report two groups of TnpB-like proteins, named as Fanzor1 and Fanzor2 (collectively called Fanzor), from diverse eukaryotic genomes, including metazoans, fungi, and protists (amoeba, chlorophyte, stramenopile, choanoflagellate and rhodophyta), as well as dsDNA viruses that infect eukaryotes. Fanzor and TnpB protein both contain a constellation of strictly conserved residues stretching from the protein center to the C-terminus, D-X(125, 275)-[TS]-[TS]-X-X-[C4 zinc finger]-X(5,50)-RD. The C4 zinc finger is called OrfB_Zn_ribbon ([CDD:pfam07282]) in the Conserved Domain Database (CDD) [18]. Phylogenetically, Fanzor1 proteins form a single separate clade, and Fanzor2 proteins co-cluster with a small set of bacterial TnpB proteins from the *IS607* family. Fanzor1 proteins were captured by transposable elements from at least five different superfamilies: *Mariner*, *Sola*, *IS4*, *Helitron* and *MuDr*. Fanzor2 proteins are encoded by the *IS607*-type transposons. While biological function of the Fanzor/TnpB proteins is not known at present, there are indications that the Fanzor1 protein may be functioning as a methyltransferase. This is based on comparison of three elements, *PGv*-*1*, *Mariner*-*2*_*PGv* and *Mariner*-*1*_*OLpv*, each encoding three proteins, including *Mariner*-Tpase, endonuclease and either methyltransferase or Fanzor1 protein. Our data also suggest that viruses may facilitate spreading Fanzor proteins in eukaryotes.

The analysis of Fanzor proteins also revealed ‘one-ended transposition’ in three non-autonomous *Helitron* transposon families (*CRe*-*1*, *2*, *3*) in green algae *Chlamydomonas reinhardtii*. Of particular interest is the ‘one-ended’ group of *Helitrons* flanked by TSDs. One-ended transposition has been previously reported in *IS91* family in bacteria [19,20], but not as associated with generation of TSDs [20]. Finally, we describe a new *Helitron* group (*Helitron2*) that is distinct from the canonical *Helitron* elements (*Helitron1*). *Helitron1* elements contain only one hairpin structure at the 3^′^-subterminal region, and with conserved 5^′^-TC and CTRR-3^′^ ends [2]. In contrast, *Helitron2* elements carry two hairpin structures and short (8 to 15 bp) asymmetric terminal inverted repeats (ATIRs) at the ends. The 5^′^-ATIR is close to the 5^′^-terminus, pairing with its downstream nucleotides to form a 5^′^-hairpin structure; the 3^′^-ATIR is subterminally located, immediately upstream from the hairpin structure. Individual *Helitron2*-like elements were reported to differ from the canonical *Helitron1* sequences in terms of their terminal features [21-24], however the features were not associated with any separate *Helitron* group. The characteristic *Helitron2* features may help improve the performance of the automatic detection programs that are currently using only the *Helitron1* features [25,26].

## Results

### Identification of the eukaryotic TnpB-like proteins

**Figure 1:**
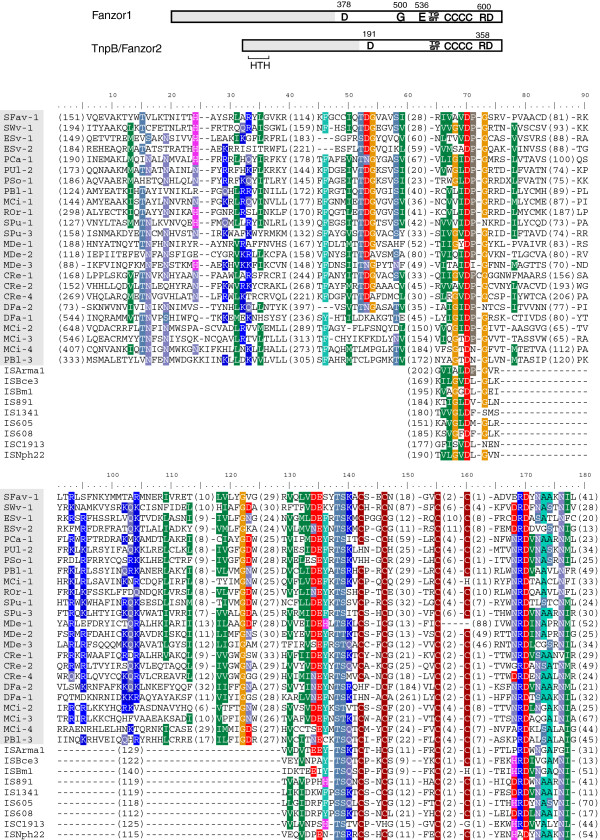
**Motifs and alignments of Fanzor and TnpB proteins.** Conserved amino acids and helix-turn-helix (HTH) domain are marked (above); gray regions indicate the variable N-terminal halves. Numbers above the diagram refer to the residue position in SPu-1-1p or TnpB_IS608. Titles of Fanzor1 proteins are shaded in the alignment.

During a systematic screening of TEs, a prototype of the eukaryotic TnpB-*IS200*/*IS605*-like protein was first discovered in the genome of the fungus *Spizellomyces punctatus*. This protein, called SPu-1-1p (633-aa), is encoded by one single open reading frame (ORF) in the *SPu*-*1* element (approximately 2,100-bp long), flanked by 33-bp terminal inverted repeats (TIRs) and putative TA target site duplications (TSDs). We identified 17 full-length *SPu*-*1* copies, approximately 92% identical to the family consensus, including nine copies with intact ORFs. The immediate homologues of the SPu-1-1p were found in some related eukaryotes, but distant homologues were identified among TnpB proteins encoded by bacterial insertions elements (ISs) from the *IS200*/*IS605* and *IS607* families (approximately 15% identity over an approximately 300 aa C-terminal region; Figure 1). To date, we have identified dozens of SPu-1-1p homologues in at least 26 diverse eukaryotic genomes, as well as in 18 large dsDNA virus species infecting eukaryotes (Table 1). The 26 eukaryotes belong to 7 taxonomic groups: metazoa, choanoflagellida, fungi, amoebozoa, chlorophyta, rhodophyta, and stramenopiles (Table 1). Hereafter, these eukaryotic TnpB-like proteins are referred to as Fanzor proteins and their bacterial counterparts are referred to as TnpB proteins. As expected, the vast majority of the Fanzor proteins, if not all of them, are encoded by TEs, which are collectively referred to as *Fanzor* elements. Consensus sequences of these elements were reconstructed whenever possible. Some elements are flanked by TIRs but others display no TIRs at their ends (see Additional file 1). In some *Fanzor* elements, a bona fide transposase is encoded along with the Fanzor protein (see Additional file 1). Therefore, based on the presence of Tpase or other characteristic DNA features most *Fanzor* elements can be classified into different superfamilies (see below). The corresponding DNA and protein sequences are listed in Additional file 2 and Additional file 3.

**Table 1:** Species harboring Fanzor sequences

**Taxon /Group**	**Species/Strain name**	**Number *Fanzor1 *family**	**Number *Fanzor2 *family**	**Element prefix**
Metazoa	*Mayetiola destructor*	13		*MDe*
	*Hydra magnipapillata*	1		*HMa*
Choanoflagellida	*Salpingoeca sp*. (*ATCC 50818*)		2	*Sal*
Fungi	*Spizellomyces punctatus*	3		*SPu*
	*Rhizopus oryzae RA 99*-*880*	4		*ROr*
	*Allomyces macrogynus ATCC 38327*	3		*AMa*
	*Phycomyces blakesleeanus NRRL1555*	3		*PBl*
	*Mucor circinelloides*	10		*MCi*
	*Ashbya gossypii ATCC 10895*		1	*Ago*
	*Eremothecium cymbalariae DBVPG*#*7215*		1	*ECy*
	*Saccharomyces cerevisiae EC1118*, *Lalvin QA23*		1	*SCe*
	*Torulaspora delbrueckii*		1	*TDe*
Amoebozoa	*Dictyostelium fasciculatum*	4		*DFa*
	*Polysphondylium pallidum PN500*	7		*PPa*
	*Acanthamoeba castellanii strain Neff*		2	*ACa*
Chlorophyta	*Volvox carteri*	2		*VCa*
	*Chlamydomonas reinhardtii*	5		*CRe*
	*Chlorella vulgaris strain NJ*-*7*	1		*CVu*
Rhodophyta	*Cyanidioschyzon merolae*	1		*CMe*
Stramenopiles	*Pythium ultimum*	6		*PUl*
	*Nannochloropsis oceanic*	1		*NOc*
	*Phytophthora sojae*	4	1	*PSo*
	*Phytophthora capsici*	2	1	*PCa*
	*Phytophthora ramorum*	1	1	*PRa*
	*Albugo laibachii Nc14*	2		*Ala*
	*Ectocarpus siliculosus*	1		*ESvi*
dsDNA virus	*Ectocarpus siliculosus virus* ([GeneBank:AF204951], 335-kb)	2		*ESv*
	*Shrimp white spot syndrome virus* ([GenBank:AF332093], 305-kb)	1		*SWv*
	*Helicoverpa armigera granulovirus* ([GenBank:EU255577], 169-kb)	1		*HAgv*
	*Helicoverpa armigera multiple nucleopolyhedrovirus* ([GenBank:EU730893], 154-kb)	1		*HAmn*
	*Pseudaletia unipuncta granulovirus* ([GenBank:EU678671], 176-kb)	1		*PUgv*
	*Spodoptera frugiperda ascovirus 1a* ([GenBank:AM398843], 157-kb)	1		*SFav*
	*Heliothis virescens ascovirus 3e* ([GenBank:EF133465], 186-kb)	1		*HVav*
	*Mamestra configurata nucleopolyhedrovirus B* ([GenBank:AY126275], 158-kb)	1		*MCnv*
	*Phaeocystis globosa virus 12T* ([GenBank:HQ634147], 460-kb)	1		*PGv*
	*Emiliania huxleyi virus 88* ([GenBank:JF974310], 397-kb)	1		*EHv88*
	*Emiliania huxleyi virus 99B1* ([GenBank:FN429076], 377-kb)		1	*EHv99B1*
	*Acanthamoeba polyphaga mimivirus* ([GenBank:AY653733], 1181-kb)		3	*APmv* (*ISvMimi*)
	*Acanthamoeba castellanii mamavirus* ([GenBank:JF801956], 1192-kb)		3	*ACmv*
	*Megavirus chiliensis* ([GenBank:JN258408], 1259-kb)		2	*MGvc*
	*Paramecium bursaria Chlorella virus AR158* ([GenBank:DQ491003], 345-kb)		2	*ISvAR158*
	*Paramecium bursaria Chlorella virus NY2A* ([GenBank:DQ491002], 369-kb)		2	*ISvNY2A*
	*Cafeteria roenbergensis virus BV*-*PW1* ([GenBank:GU244497], 617-kb)		1	*CRv*-*1*
	*Feldmannia species virus* ([GenBank:NC_011183], 155-kb)		1	*FEsv*-*1*

### Sequence feature and phylogeny of Fanzor proteins

The N-terminal halves of the Fanzor and TnpB proteins are highly diverged, but their C-terminal halves are relatively conserved and include strictly conserved amino acid motif D-X(125, 275)-[TS]-[TS]-X-X-[C4 zinc finger]-X(5, 50)-RD (Figure 1, see Additional file 4). To date, this long motif was found only in TnpB and Fanzor proteins, and it includes a short, previously characterized OrfB_Zn_ribbon domain ([CDD:pfam07282]). Given that Fanzor and TnpB are both associated with TEs, the shared motif strongly suggests that they are functional homologues, rather than unrelated proteins accidentally carrying the same domain.

**Figure 2:**
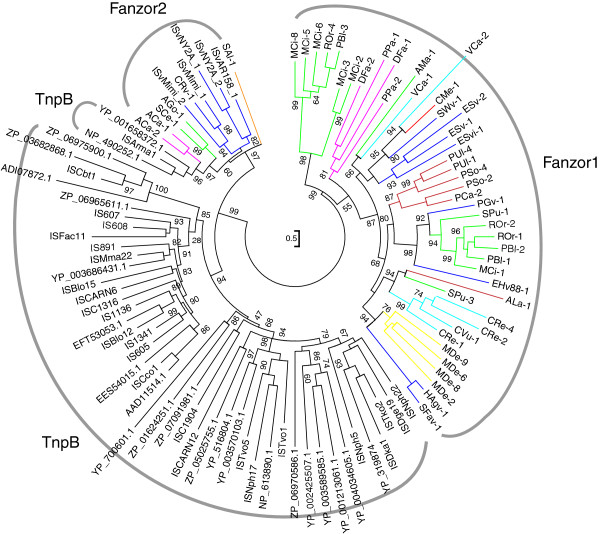
**Phylogeny of Fanzor/TnpB proteins.** Eukaryotes and eukaryotic viruses are colored as follows: dsDNA viruses (blue), metazoa (yellow), fungi (green), chlorophyta (cyan), rhodophyta (red), stramenopiles (dark red), choanoflagellida (orange), and amoebozoa (pink). TnpB proteins are from the ISfinder database (ISXXX) or GenBank (with accession number). The tree is based on the alignment of a longer region including most of the N-terminal and the C-terminal portions (see Additional file 4).

Fanzor proteins are divided into two distinct clades, Fanzor1 and Fanzor2 (Figure 2), as indicated by the phylogenetic tree based on the nearly entire sequence lengths (see Additional file 4). The major Fanzor1 clade consists exclusively of eukaryotic proteins. In contrast, the minor Fanzor2 clade co-clusters with several TnpB proteins from the prokaryotic *IS607* family, such as the *ISArma1* element. The co-clustering of Fanzor2 and TnpB is not caused by sequence contamination, because multiple proteins are found in each category. Apart from the few TnpB proteins co-clustered with Fanzor2 clade, all the other TnpB proteins are out-grouped together. Notably, virus-borne Fanzor proteins come from both Fanzor clades (Figure 2). For example, two different strains of one virus: *Emiliania huxleyi* virus 88 and *Emiliania huxleyi virus* 99B1, carry *EHv88*-*1* element from the Fanzor1 clade, and *EHv99B1*-*1* element from the Fanzor2 clade, respectively (see Additional file 1). On the other hand, highly similar Fanzor proteins can be found in viruses with completely different genomic sequences. For example, *HVav*-*1* element is 88% identical to *HAmn*-*1* over the entire length. However, the two hosting virus genomes ([GenBank:EF133465] and [GenBank:EU730893], respectively) share no detectable similarities at all.

Helix-turn-helix (HTH) domain ([CDD:pfam12323]: HTH_OrfB_IS605) is present in the N-terminal regions of some TnpB and Fanzor2 proteins (Figure 1), including those encoded by *IS607*, *IS891*, *ISArma1* and *ISvAR158*_*1*. Given that the alignment in this local area is relatively well conserved (see Additional file 4), this HTH domain is presumably present in other TnpB proteins, but due to the high sequence divergence whether or not a comparable HTH domain exists in Fanzor1 proteins could not be determined. Two additional amino acids are also extremely conserved in the Fanzor1 proteins (G500 and E536, Figure 1). However, this may reflect a smaller divergence of the Fanzor1 clade than that of the TnpB clade (Figure 2).

### Fanzor1 protein in *Tc*/*mariner* elements

**Figure 3:**
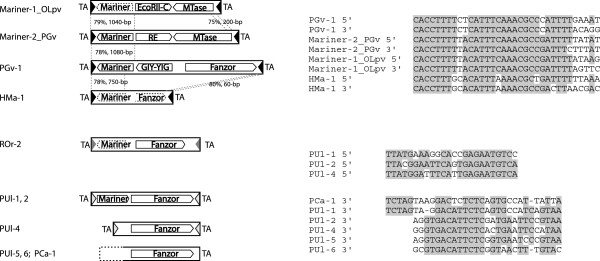
**Fanzor proteins in *Mariner *elements.** Transposable elements (TEs) are indicated by bars flanked by TA target site duplications (TSDs); the undetermined ends are indicated by dash lines (*PUl*-*5*, *6*; *PCa*-*1*). The triangles at the element ends represent the terminal inverted repeats (TIRs) sequences. The inner arrows indicate the protein coding regions (dashed lines indicate the degenerated coding sequences). The alignments of the 5^′^ and 3^′^ TIRs sequences are shown on the right.

Some *Fanzor1* elements, such as *PGv*-*1* and *PUl*-*1* (Figure 3, see Additional file 5), encode both the Fanzor1 protein and a *Mariner*-like Tpase. Other elements, such as *PUl*-*4*, encode Fanzor1 proteins only but carry TIRs identical to confirmed members of the *Mariner* family, and all are flanked by TA TSDs, a hallmark of the *Mariner* transposons (Figure 3). The most interesting examples are four related, single-copy *Mariner* elements, including *PGv*-*1*, *Mariner*-*2*_*PGv*, *Mariner*-*1*_*OLpv* and *HMa*-*1*. The four elements share significant sequence similarity in their TIRs and 5^′^-terminal regions (approximately 78% identical, 1 kb long) coding for the *Mariner* Tpases (Figure 3), but they differ in their 3^′^ portions. Nevertheless, two proteins encoded by the 3^′^ portions of the former three *Mariner* elements appear to be functionally comparable. For example, the first of the two 3^′^ proteins (suffixed ‘2p’) encoded by *PGv*-*1*, *Mariner*-*2*_*PGv* and *Mariner*-*1*_*OLpv* are endonucleases and the other proteins (suffixed ‘3p’) in *Mariner*-*2*_*PGv* and *Mariner*-*1*_*OLpv* are methyltransferases. Specifically, PGv-1-2p (291-aa) contains a GIY-YIG nuclease [27] domain ([CDD:cl15257]) at its N-terminus (E-value = 4.44e-09; see Additional file 6). Mariner-2_PGv-2p (256-aa) is annotated as a hypothetical restriction endonuclease in the REBASE database (The Restriction Enzyme Database) [28]. Mariner-1_OLpv-2p (198-aa, [GenBank:ADX06147.1]) contains the C-terminal catalytic domain of the restriction endonuclease EcoRII ([CDD:pfam09019]), which is well supported by the sequence alignment despite of the low score (E-value = 1.1) in CDD database (see Additional file 7). Mariner-2_PGv-3p (459-aa, [GenBank:AET72984.1]) contains the methyltransferase domain Methyltransf_26 ([CDD:pfam13659]; E-value: 9.61e-08), and Mariner-1_OLpv-3p (344-aa, [GenBank:ADX06148.1]) contains the Cyt_C5_DNA_methylase domain ([CDD:cd00315]; E-value: 8.99e-72). Based on this parellelism (Tpase, endonuclease and methyltransferase), one possibility is that the third protein encoded by *PGv*-*1* (that is, Fanzor protein) is also a methyltransferase. Notably, *HMa*-*1* also might have originated from an unknown virus despite the fact that it is found in the *Hydra magnipapillata* contig sequence ([GenBank:ABRM01000004.1], 154-kb), because the closest relatives of the multiple upstream and downstream proteins, flanking the *HMa*-*1* element, are also viral proteins.

### Fanzor1 protein in *Helitron* transposons

**Figure 4:**
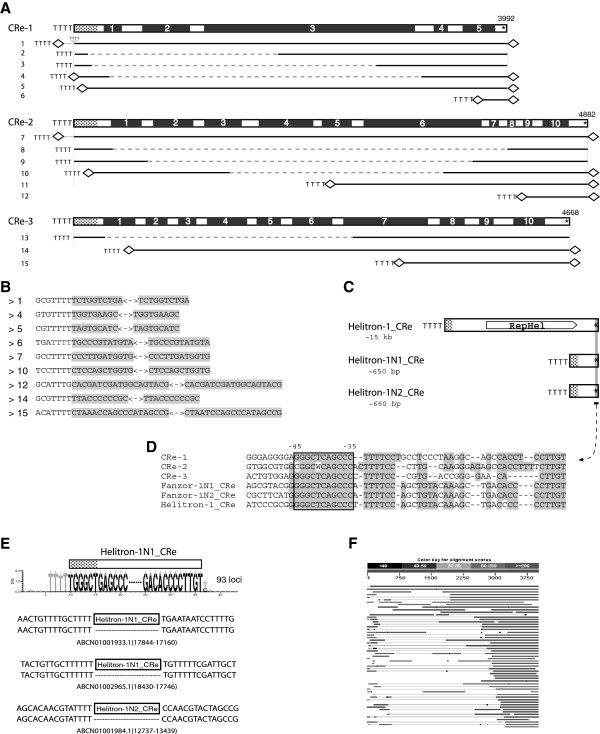
***CRe***-***1, 2, 3 *elements.** (**A**) *CRe*-*1*, *2*, *3* consensus sequences and the exons (black boxes). Dotted areas indicate the 5^′^-ends, approximately 200- bp long, which are 98% identical to those of confirmed *Helitrons*. Asterisks at the 3^′^-ends indicate the short homologous regions in *CRe*-*1*, *2*, *3* and *Helitron* elements (**C**, **D**). The corresponding sequences of the15 example loci (1 to 15) are indicated by solid lines below. Dashed lines mark the internal deletion regions. Nine of them are flanked by target site duplications (TSDs) indicated by small diamonds. Note that locus 1 and 7 include short segments of ‘non-Fanzor’ sequences (gray line) at the 5^′^-ends. The sequences of the 15 loci are shown in Additional file 8B. (**B**) Examples of the nine TSD sequences (shaded). Note that the 5^′^-TSDs are immediately downstream of TTTT tetra-nucleotides. (**C**) *Helitron*-*1*_*CRe and* non-autonomous *Helitrons*. (**D**) The alignment of the 3^′^-ends of *Helitrons* and *CRe*-*1*,*2*,*3*. The 3^′^ asymmetrical terminal inverted repeats (ATIRs) are boxed. (**E**) Target specificity of *Helitron*-*1N1*_*CRe* elements. They insert specifically between TTTT and T/C and produce no TSDs. *Helitron*-*1N2*_*CRe* elements also insert after TTTT. Three examples of the pre- and post-insertion sites are shown. (**F**) The illustration of the 5^′^-truncation or 3^′^-overabundance in *CRe*-*1* elements: graphical summary of a NCBI online BLASTN search of the *Chlamydomonas reinhardtii* genome with the consensus of *CRe*-*1*.

There are three *Fanzor1* elements (*CRe*-*1*, *2*, *3*) in the genome of single-celled green alga *Chlamydomonas reinhardtii*, which most likely represent non-autonomous *Helitron* transposons (specifically, *Helitron2* group of transposons described below). Their 5^′^-end 200-bp, and 3^′^-end 50-bp sequences, are highly similar (approximately 90% and 70% identity, respectively), to those of verified *Helitrons* (that is, *Helitron*-*1*_*CRe*, *Helitron*-*1N1*_*CRe* and *Helitron*-*1N2*_*CRe*; Figure 4D, see Additional file 8A). The Fanzor1 proteins are encoded by five exons in *CRe*-*1* element, and by ten exons in *CRe*-*2* and *CRe*-*3* elements (Figure 4A). These exons are supported by a number of expressed sequence tags (EST).

The three *Fanzor1* families (*CRe*-*1*, *2*, *3*) are frequently 5^′^-truncated, and coupled with internal deletions (Figure 4A, 4F, see Additional file 8B). However, almost all copies are intact at the 3^′^-terminal regions (Figure 4F). This biased 3^′^-overabundance implies that duplication process by the rolling cycle replication starts from the 3^′^-end, which is analogous to the previously reported one-ended transposition in bacterial *IS91* element [20]. Data from *Helitron*-*1N1*_*CRe* and *Helitron*-*1N2*_*CRe* indicate that these *Helitrons* insert specifically downstream from the 5^′^-TTTT-3^′^ tetranucleotide, producing no TSDs (Figure 4E). However, this non-TSD feature only appears in *CRe*-*1*, *2*, *3* insertions that terminate exactly at the consensus 5^′^-ends, such as the loci 2, 3, 8, 9, 13 in Figure 4A. Strikingly, most other insertions, especially 5^′^-truncated ones, are flanked by TSDs of variable length (approximately 7 to 19 bp; Figure 4B). In some cases much longer TSDs are observed (44, 50, 93, 242 and 443-bp long). Approximately 70% of *CRe*-*1* (150 loci), 57% of *CRe*-*2* (70 loci), and 10% of *CRe*-*3* (35 loci) are flanked by TSDs. This varying percentage probably reflects different family ages, since *CRe*-*1* is the youngest family with elements approximately 98% identical to the consensus. Interestingly, almost all of these 5^′^-TSDs are located downstream from the same tetranucleotide as observed in the *Helitron*-*1N1*_*CRe* or *Helitron*-*1N2*_*CRe* insertions (TTTT, or T-rich tetranucleotides: TTTG, TTTC, TCTT, TGTT), suggesting a common mechanism involved at least in the target recognition process, in the *Helitron* and the three non-autonomous *Fanzor1* families. In some individual *CRe*-*1*, *2*, *3* insertions, short extra sequences are present downstream the 5^′^-TSDs (locus 1 and 7, Figure 4A). The captured sequences can occur upstream from the normal consensus 5^′^-termini (locus 1, Figure 4A). Intriguingly, TSDs are extremely rare in the cases of the non-autonomous *Helitron*-*1N1*_*CRe* and *Helitron*-*1N2*_*CRe* elements. For example, only one out of 200 *Helitron*-*1N1*_*CRe* elements is flanked by TSDs. Elements of the two families are 95 to 98% identical to their consensus sequences. It is not clear whether the difference between the three *Fanzor1* elements and the two non-autonomous *Helitron* elements is caused by the Fanzor1 protein or by the relatively short length of the *Helitron*-*1N1*_*CRe* elements (657 bp) or *Helitron*-*1N2*_*CRe* elements (673 bp).

### Features of *Helitron2* elements

**Figure 5:**
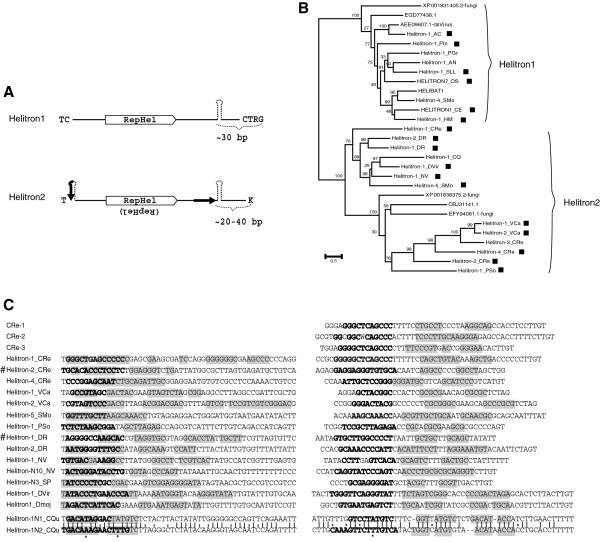
***Helitron2 *subgroup and its terminal structure.** (**A**) Structural features of *Helitron1* and *Helitron2* groups. *Helitron1* has only 3^′^-subterminal hairpin structure [2]. The dark arrows in *Helitron2* represent the asymmetric terminal inverted repeats (ATIRs). (**B**) Phylogeny of the RepHel proteins encoded by *Heliron1* and *Helitron2* groups. The black square indicates *Helitrons* in which both termini are known. The alignment of the RepHel proteins is shown in Additional file 9. (**C**) Terminal sequences from the selected examples of *Helitron2* elements. The bases in bold font represent the ATIRs. Pairing nucleotides in the hairpins are shaded in gray. The 5^′^-end sequences of *CRe*-*1*, *2*, *3* are similar to that of *Helitron*-*1*_*CRe* (not shown). Note that the RepHel protein is encoded in the opposite direction in *Helitron*-*2*_*CRe* and *Helitron*-*1*_*DR* (marked with #). The compensatory mutations in the complementary segments are highlighted by asterisks at the bottom in the alignment of *Helitron*-*1N1*_*CQu* and *Helitron*-*1N2*_*CQu*.

*CRe*-*1*, *2*, *3* and many other *Helitron* elements from different species, such as *Helitron*-*1*_*CRe*, *Helitron*-*2*_*CRe* and *Helitron*-*5*_*SMo*, display two distinct features at the terminal regions. The first one is called short asymmetrical terminal inverted repeats (ATIRs), located asymmetrically at the ends: the 5^′^-ATIR is 0 to 2 bp away from to the 5^′^-end, and the 3^′^-ATIR is approximately 20 to 30 bp apart from the 3^′^-end, upstream of the hairpin structure (Figure 5A, 5C). The second feature is the 5^′^-terminal hairpin structure, involving a part or the whole 5^′^-ATIR sequence (Figure 5A, 5C). The two structural features are assumed to be important for transposition. Particularly, compensatory base mutations were observed in two related elements (that is, *Helitron*-*1N1*_*CQu* and *Helitron*-*1N2*_*CQu*) to maintain such features (Figure 5C). Possibly, during the ending phase of the rolling cycle replication, the pairing between the 5^′^-ATIR and 3^′^-ATIR destroys the 5^′^-hairpin structure, and thus determines the replication endpoint. All *Helitrons* with such features are significantly clustered in one phylogenetic group, called *Helitron2* in this paper, whereas all *Helitrons* with the canonical structures constitute a separate group (*Helitron1*), with elements lacking the 5^′^-hairpin structure [2] (Figure 5A, 5B; see Additional file 9). Nevertheless, both *Helitron1* and *Helitron2* elements have 3^′^-terminal hairpin structures, and show similar 5^′^-end nucleotide preferences: TC in *Helitron1* and T in *Helitron2*. With this hindsight, the *CRe*-*1*, *2*, *3* elements are confirmed as *Helitron2* transposons (Figure 5C). It is worth noting that in some *Helitron2* elements, such as *Helitron*-*2*_*CRe* and *Helitron*-*1*_*DR*, the RepHel protein is in the opposite orientation relative to the majority (Figure 5A, 5C).

### Fanzor1 protein in *IS4*-type elements

**Figure 6:**
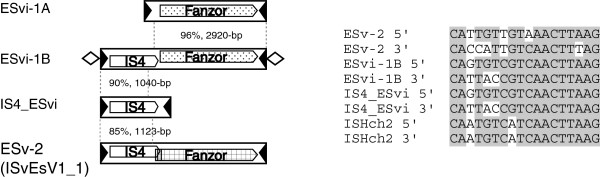
**Fanzor1 protein in *IS4 *elements.***IS4*-type elements in *Ectocarpus siliculosus* and *Ectocarpus siliculosus virus*-*1*. The alignment of terminal inverted repeats (TIRs) is shown on the right. The 8-bp perfect TSDs flanking *ESvi*-*1B* are indicated by diamonds. Note that the *ESv*-*2* element is named as *ISvEsV1*_*1* in the ISfinder database, where the encoded Fanzor1 protein is annotated as a passenger protein of unknown function.

*IS4*-type Tpase and *Fanzor1* proteins are present in two families, *ESvi*-*1B* and *ESv*-*2* (Figure 6). The two families are in the genome of brown algae *Ectocarpus siliculosus* and algae virus *Ectocarpus siliculosus virus*-*1* (ESV1, [GenBank:AF204951]), respectively. Other related elements, either encoding Fanzor1 or *IS4*-type Tpase, such as *ESvi*-*1A* and *IS4*_*ESvi*, are also found in the algae genome (Figure 6). All these elements are single-copy in the genomes, flanked by 18-bp terminal inverted repeats (TIRs) similar to those of *ISHch2* element, which is annotated as *IS4* family in the ISfinder database (Figure 6). *ESvi*-*1B* and *ESv*-*2* elements share approximate 1 kb long 5^′^-terminal sequences coding for *IS4*-type Tpase (78% sequence identity), but differ completely in the other regions, where *Fanzor1* proteins are encoded. This situation is analogous to that between *PGv*-*1* and *Mariner*-*2*_*PGv* elements described above (Figure 3). Notably, although *ESvi*-*1A*, *ESvi*-*1B* and *IS4*_*ESvi* elements were identified in the genome of brown algae *E*. *siliculosus*, they should be viewed as virus-borne elements (‘vi’ in each name stands for ‘virus integrated’). They are found in two contig sequences ([GenBank:CABU01010405.1] and [GenBank:CABU01010404.1]) that are approximately 84% identical to the ESV1 virus genome ([GenBank:AF204951]), likely representing large integrated virus fragments [29]. Besides, there is another *Fanzor* family in the ESV1 genome, *ESv*-*1*, probably associated with non-*IS4* families. Individual elements from the *ESv*-*1* family are flanked by 2-bp TSDs (TA) and variable TIRs.

### Fanzor1 protein in *Sola2* elements

**Figure 7:**
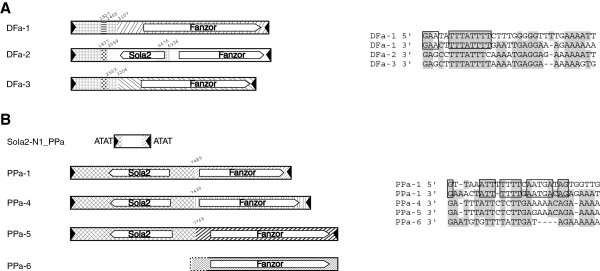
**Fanzor1 protein in *Sola2 *elements from (A) *Dictyostelium fasciculatum *and (B) *Polysphondylium pallidum*.** Similar regions are indicated by the same type of patterns. 5′ and 3′ terminal inverted repeats (TIRs) sequences are shown on the right.

In the amoebozoa *Dictyostelium fasciculatum*, there are three related *Fanzor1* families (*DFa*-*1*, *2*, *3*) classified as the *Sola2*-type elements [30]. A putative 888-aa *Sola2*-type Tpase is encoded by the *DFa*-*2* elements (Figure 7A, see Additional file 10). Moreover, the three families are flanked by 12 or 13-bp TIRs and AT-rich 4-bp TSDs (AWWT) (Figure 7A, see Additional file 11). The 4-bp TSDs feature is consistent with that of *Sola2* family [30]. In *DFa*-*1* and *DFa*-*3* elements most of the *Sola2*-Tpase coding region is deleted. The three families are nearly identical in the 5^′^ regions (approximately 2.5 to 3 kb from the 5^′^-end), but no sequence similarity was detected in most other regions, where Fanzor1 proteins are encoded (Figure 7A). Interestingly, such *Sola2*-Fanzor chimeric elements also appear in *PPa*-*1*, *4*, *5* families in amoebozoa species *Polysphondylium pallidum* (Figure 7B, see Additional file 10). Among them, the 5^′^-terminal 7-kb sequences are nearly identical (98% identity), coding for *Sola2* Tpases, but the 3^′^-terminal sequences are entirely different. These chimeric elements are flanked by short imperfect TIRs (21-bp), and 4-bp AT-rich TSDs (that is, ATAT, AAAT, ATTT; Figure 7B, see Additional file 11).

### Fanzor1 protein in other transposable elements

*Fanzor1* proteins were also found in DNA transposons from other superfamilies. For example, in the genomes of fungi *Rhizopus oryzae*, *Phycomyces blakesleeanus* and *Mucor circinelloides*, *ROr*-*4*, *PBl*-*3* and *MCi*-*4* elements, respectively, appear to belong to the *MuDr* superfamily (see Additional file 12). While these elements do not encode *MuDR* Tpase, all carry TIRs similar to those of confirmed *MuDR* elements (for example, *MuDr*-*2*_*PBl*) and are flanked by 9-bp TSDs.

In the genomes of five insect-infecting viruses, five closely related *Fanzor1* families, *HVav*-*1* (*Heliothis virescens ascovirus 3e*), *SFav*-*1* (*Spodoptera frugiperda ascovirus 1a*), *PUgv*-*1* (*Pseudaletia unipuncta granulovirus*), *HAgv*-*1* (*Helicoverpa armigera granulovirus*) and *HAmn*-*1* (*Helicoverpa armigera multiple nucleopolyhedrovirus*), are flanked by 4-bp TSDs (TTAN) and 13-bp TIRs (see Additional file 13). However, they could not be assigned to any particular superfamily due to the lack of Tpase information.

**Figure 8:**
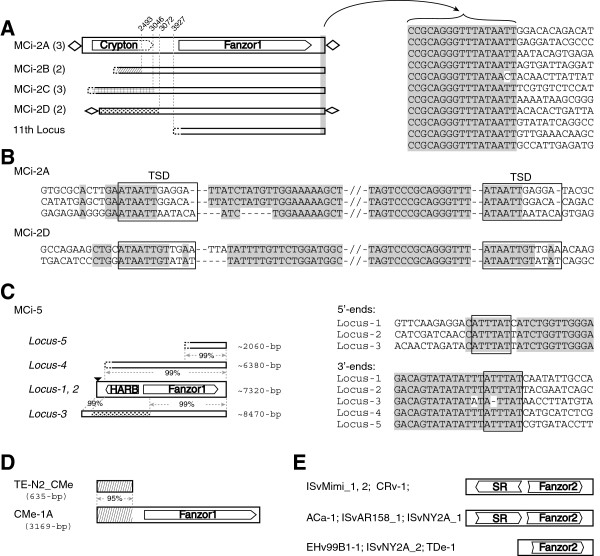
**Other diverse *Fanzor *elements.** (**A**) *MCi*-*2* family (left), and alignment of their 3^′^-ends (right). Copy numbers of subfamilies are indicated in parentheses. Different 5^′^ regions are indicated by different patterns. Undetermined termini are indicated by dashed lines. (**B**) Target site duplications (TSDs) sequences of the *MCi*-*2A* and *MCi*-*2D* subfamilies. (**C**) *MCi*-*5* elements and their lengths. Undetermined element ends are indicated by dashed lines. The alignments of the 5^′^ and 3^′^ ends are shown on the right. The black triangle above locus-1 and locus-2 indicates small insertions ( approximately 520 bp) relative to locus-3. (**D**) Relationship between *CMe*-*1A* and *TE*-*N2*_*CMe*. (**E**) Serine recombinases (SR) encoded by some *Fanzor2* elements. HARB; Harbinger Tpase.

In the genome of the fungus *Mucor circinelloides*, *MCi*-*2* family is unclassified due to its unusual features (Figure 8A). A total of 11 *MCi*-*2* copies (loci) are found in the genome. They differ in the 5^′^ regions (approximately 1 to 3 kb long), but are nearly identical in their 6-kb 3^′^ regions (99% identity), where Fanzor proteins are encoded. Based on their 5^′^ variable regions, four subfamilies were identified out of ten loci (*MCi*-*2A*, *2B*, *2C*, and *2D*), where each subfamily is represented by two or three copies. The 11th locus is probably incomplete, and it is represented by a single copy in the genome (Figure 8A). The *MCi*-*2A* and *MCi*-*2D* subfamilies are represented by three and two presumably complete copies, respectively. They are flanked by 11 or 12-bp TSDs (Figure 8B), but they lack recognizable TIRs. The TSDs show the same pattern, ATAATTNNNN(N), implying that the two subfamilies use the same mechanism of transposition, though they have different 5^′^-end sequences. Notably, although the *MCi*-*2A* subfamily contains a partial coding sequence for a *Crypton* Tpase (approximately 473-aa), it lacks approximately 200-aa at its C-terminus when compared to other fungal *Crypton* Tpases (see Additional file 14). It remains uncertain if the *MCi*-*2* elements belong to the *Crypton* superfamily, because *Cypton* elements have not been known to produce TSDs. Moreover, it is unclear whether the 5^′^ fuzzy ends of *MCi*-*2A* and *MCi*-*2D* result from incomplete transposition/duplication or if there are other reasons (Figure 8B).

As in the case of *MCi*-*2*, the classification of *MCi*-*5* family is also unknown. Five *MCi*-*5* elements (loci) were identified in *M*. *circinelloides* genome, three of which (*Locus*-*1*, *2*, *3*) appear to be complete elements, flanked by putative 6-bp TSDs (ATTTAT), while no significant TIRs were detected (Figure 8C). Interestingly, the *Harbinger*-type Tpase (2 exons) is encoded by three *MCi* elements (*Locus*-*1*, *2*, *4*; Figure 8C, see Additional file 15). It is unclear whether the *Harbinger* Tpases are involved in the transposition of *MCi*-*5* elements, because, in contrast to other typical *Harbinger* elements, *MCi*-*5* elements lack any obvious TIRs, and the potential TSDs (ATTTAT) are not 2 or 3-bp long as in other typical *Harbinger* elements [31].

In the red alga *Cyanidioschyzon merolae* genome, approximately 150 copies of *CMe*-*1A* elements are found, each approximately 80% identical to the consensus. Its complete consensus is shown to be around 3-kb long, but the TSDs could not be determined, probably due to high diversity. Interestingly, the 5^′^ 635-bp of *CMe*-*1A* is 95% identical to the entire sequence of another transposable element, *TE*-*N2*_*CMe*, which is represented approximately by 70 copies in the genome (Figure 8D). Both *CMe*-*1A* and *TE*-*N2*_*CMe* elements lack TIRs and their TE classification is unknown.

### Fanzor2 proteins in *IS607*-like elements

Except for the Fanzor2 proteins, the only TnpA_*IS607*-like serine recombinases (SR) could be found in some *Fanzor2* elements, such as *ACa*-*1*, -*2*, *CRv*-*1*, *ISvMimi*_*1*, *ISvMimi* _*2*, *ISvAR158*_*1*, and *ISvNY2A*_*1* (Figure 8E, see Additional file 1). In the bacterial IS elements that co-cluster with *Fanzor2* elements, only TnpA_*IS607*-like serine recombinases (SR) were found, such as in *ISArma1* (Figure 2). All these elements have no TIRs or TSDs, suggesting *Fanzor2* and these IS elements might have a common origin.

## Discussion

### The mysterious role of Fanzor/TnpB in transposition

Prokaryotic TnpB proteins are encoded by bacterial transposable elements of *IS200*/*605* or *IS607* family. Here we report two groups of TnpB homologues (Fanzor1 and Fanzor2) encoded by diverse transposable elements from different eukaryotic species, as well as from some large DNA viruses that infect eukaryotes. Fanzor and TnpB proteins are functionally uncharacterized, but they share the same set of extremely conserved motifs in their C-terminal halves: D-X(125,275)-[TS]-[TS]-X-X-[C4 zinc finger]-X(5,50)-RD (Figure 1). While Fanzor2 proteins are closer to prokaryotic TnpB, also encoded by *IS607*-like elements, Fanzor1 proteins are encoded by diverse TEs, and are more distantly related to TnpB than the Fanzor2 proteins (Figure 2).

TnpB/Fanzor proteins are not DDE-type Tpases. Why are they so frequently found in various transposons? Can Fanzor/TnpB represent a novel type of Tpase that could propagate DNA element alone? This possibility can be ruled out in *IS200*/*605* or *IS607* families, where tyrosine recombinase or serine recombinase (TnpA) is known to be the functional Tpase, and TnpB proteins appear to be dispensable for transposition [14-16,32]. Alternatively, could TnpB/Fanzor represent a captured passenger gene with functions irrelevant for the transposition process, such as antibiotic resistance genes? This is also unlikely because they would be present in many different types of IS elements, rather than only in *IS200*/*605* and *IS607* families from bacterial genomes.

In a third scenario, TnpB/Fanzor proteins may function as regulatory proteins in an unknown transposition processes *in vivo*. In fact, the complexity of the transposition process has been studied in *Tn7* transposon, which encodes five proteins and all are involved in transposition. The proteins are: TnsA (type II restriction endonuclease), TnsB (DDE Tpase), TnsC (a regulator between the TnsAB and TnsD or TnsE), TnsD (directing transposition to attTn7 sites) and TnsE (directing transposition to non-attTn7 sites) [33]. Other transposon-encoded non-Tpase proteins, potentially involved in transposition were also reported recently by Kapitonov *et al*. [34]. They include the SNF2 helicase in *Inton* and *Enton*, DEDDh nuclease in *P* and *piggyBac*, and RecQ Helicase in *Academ*. It is worth noting that Fanzor/TnpB proteins contain some DNA-binding domains: a zinc-finger-like domain near to the C-termini, and a N-terminal HTH domain in TnpB and Fanzor2 (probably, in the Fanzor1 proteins as well; Figure 1), suggesting their involvement in the transposition process.

The presumed function in transposition is also suggested by an example of an old *Fanzor1* family, *CMe*-*1A* (Figure 8D). *CMe*-*1A* elements are approximately 80% identical to the family consensus, but some individual *CMe*-*1A* elements still encode intact Fanzor1 proteins. This long lasting coding capability would seems unusual for a “non-autonomous” family (*CMe*-*1A*) if no function is associated with the Fanzor1 protein. Analogous cases exist in the so-called *HAL1* “non-autonomous” families derived from the *L1* non-LTR retrotransposons, which encode the first open reading frame protein (ORF1p) only, instead of both ORF1p and ORF2p [35]. ORF1p is a “nucleic acid chaperone with RNA binding [36] and nucleic acid chaperone activity [37], but ORF2p codes for the major Tpase with its endonuclease (EN) and reverse transcriptase (RT) activity. In the guinea pig genome the coding capacity of the ORF1p in the *HAL1* retrotransposons has been maintained for a relatively long time (approximately 29 to 44 Myr) [35], implying that both the *cis*-encoded ORF1p and trans-encoded ORF2p are required for transposition of *HAL1* elements.

Comparison of three virus-integrated *Mariner* transposons, *PGv*-*1*, *Mariner*-*2*_*PGv* and *Mariner*-*1*_*OLpv* (Figure 3) may provide some clues regarding the potential function of the TnpB/Fanzor protein. Each *Mariner* element encodes three proteins showing some functional parallelisms: Tpase, endonuclease, and methyltransferase in *Mariner*-*2*_*PGv* and *Mariner*-*1*_*OLpv* or Tpase, endonuclease and Fanzor in *PGv*-*1*. In bacteria, methytransferases and restriction endonucleases constitute the restriction-and-modification system important in many cellular processes. Therefore, it is interesting to see that both endonuclease and methyltransferase are encoded by some transposons (*Mariner*-*2*_*PGv* and *Mariner1*-*1*_*OLpv*). To our knowledge, the presence of methyltransferase in transposons has not been reported before. The potential role of the transposon-encoded methyltransferases in transposition remains largely unknown. Normally, DNA methylation is essential for inhibiting the expression and transposition of TEs [38,39]. For example, methylation in the terminal sequence of transposons can prevent binding of transposase [40,41]. Theoretically, methylation may also protect the DNA in transposome from cutting by restriction enzymes, especially in bacterial cells. Moreover, it was reported that deoxycytosine methylase (Dcm) and EcoRII methylase could increase the *Tn3* transposition frequency in *E*.*coli*[42]. There are other circumstantial data consistent with this methyltransferase-hypothesis. First, while the vast majority of TnpB proteins are annotated as transposases in the NCBI database, a handful of them are indeed annotated as DNA (cytosine-5-)-methyltransferases (for example, [GenBank:YP_001645687.1]). However, the basis for this annotation is not documented. Second, GipA ([GenBank:AAF98319.1]) is a TnpB-like protein encoded by an IS element carried by the lambdoid phage Gifsy-1. GipA has been shown to be a virulence gene in *Salmonella enterica*[32]. Analogously, DNA adenine methylase (Dam) is known as an important factor in bacterial virulence [43-45]. The above observations are consistent with the possibility that Fanzor protein could be a methytransferase.

### *Fanzor* elements in viruses

In the current dataset, 18 different large dsDNA eukaryotic viruses were found carrying *Fanzor* elements (Table 1). In contrast, only 24 eukaryotic species are found carrying *Fanzor* elements. This is unexpected given the relatively small genomes of these viruses. However, this may be partly explained by a possibility that Fanzor protein assumes the same role both in the viral infection and TE transposition. In a sense, both viruses and DNA TEs are selfish or parasitic episomes.

In the phylogenetic tree, the viral Fanzor proteins are intermingled with non-viral eukaryotic Fanzor proteins (Figure 2). This suggests that these large-genome viruses may play an extensive role in spreading *Fanzor* genes (or other TEs) among eukaryotes. Among currently sequenced metazoan species, only one insect species, hessian fly (*M*. *destructor*), was found to carry *Fanzor* elements. The *HMa*-*1* element in *H*. *magnipapillata* probably originally also came from a virus genome. All the 13 *Fanzor* families in the *M*. *destructor* genome significantly co-cluster with 5 viral *Fanzor* families, including *HAgv*-*1*, *SFav*-*1*, *PUgv*-*1*, *HAmv*-*1* and *HVav*-*1* (*PUgv*-*1*, *HAmv*-*1* and *HVav*-*1* are not included in Figure 2). These viruses are all insect-infecting viruses suggesting that they may participate in spreading *Fanzor* elements. Interestingly, the genomes of *Heliothis virescens* ascovirus 3e (HVav, [GenBank:EF133465]) and *Helicoverpa armigera* multiple nucleopolyhedrovirus (HAmn, [GenBank:EU730893]), share no overall sequence similarity at all, but each of them contains one copy of a *Fanzor* element, *HVav*-*1* and *HAmn*-*1*, respectively, 88% identical to each other over the entire length. Notably, the two viruses infect insect species of the same *Noctuidae* family. Finally, the *Phaeocystis globosa* virus 12T (PGv, [GenBank:HQ634147]) and Organic Lake phycodnavirus 1 (OLpv-1, [GenBank:HQ704802.1]) genome share no overall sequence similarity at all, except for the *Mariner*-*2*_*PGv* and *Mariner1*_*OLpv* elements in their genomes, respectively, which are 79% identical in their 5^′^-terminal regions (Figure 3). Both viruses infect phototrophic marine algae: PGv infects *Phaeocystis globosa* and OLpv-1 probably infects prasinophyte *Pyramimonas*[46].

Fanzor proteins are often found in chimeric elements represented by the following 4 sets of TEs: (1) *PGv*-*1*, *Mariner*-*2*_*PGv*, *Mariner*-*1*_*OLpv* and *HMa*-*1* (Figure 3); (2) *ESvi*-*1B* and *ESv*-*2* (Figure 6); (3) *DFa*-*1*, *DFa*-*2* and *DFa*-*3* (Figure 7A); (4) *PPa*-*1*, *PPa*-*4* and *PPa*-*5* (Figure 7B). The first two sets are from the virus genomes. The latter two sets of elements are present in two related slime mold species: *D*. *fasciculatum* and *P*. *pallidum*. These chimeric *Fanzor* elements probably also originated with the involvement of viruses.

## Conclusions

Fanzor and TnpB are homologous proteins. Hypothetically, they may function as methytransferases. Eukaryotic Fanzor proteins are associated with many diverse eukaryotic viruses. The relatively small number of *Fanzor* elements in Eukaryotes probably reflects the fact that they were relatively recently transferred by viruses. A more frequent horizontal transfer in bacteria may account for the more common presence of the TnpB proteins in diverse bacteria and phages [47,48]. The two clades of *Fanzor* elements (*Fanzor1 and Fanzor2*), might have originated from two independent transfers from bacteria to eukaryotes.

## Methods

Transposons were automatically detected using custom-made scripts based on the methods described before [49]. Consensus sequences of each family were constructed whenever possible. Potentially new TE proteins encoded by long ORFs, were screened out by TblastN against Rebase database [50]. The PSI-Blast and TBLASTN screening for homologous proteins was done against all available sequence databases at the National Center for Biotechnology Information (NCBI) and at the Department of Energy Joint Genome Institute (JGI). To detect all distantly related eukaryotic proteins, multiple rounds of PSI-Blast were performed until no more new significant scores were detected. Each newly detected eukaryotic protein was used as query to repeat this procedure. In addition to NCBI databases, the following genome sequences were downloaded from the JGI:, *Phycomyces blakesleeanus* NRRL1555 and *Mucor circinelloides* (http://genome.jgi-psf.org/Phybl2/Phybl2.download.ftp.html, http://genome.jgi-psf.org/Mucci2/Mucci2.download.ftp.html). The TE-encoded multiple-exon genes were predicted by FGENESH program (http://linux1.softberry.com/berry.phtml?topic=fgenesh&group=programs& subgroup=gfind), and confirmed or refined with expressed sequence tag (EST) information whenever possible. Functional motifs in these proteins were identified by search against the Conserved Domain Database (CDD) (http://www.ncbi.nlm.nih.gov/cdd/). Multiple protein sequences were aligned by online MAFFT (v6.861b), using Web server (http://mafft.cbrc.jp/alignment/software/) [51]. Sequence phylogenies were obtained using PhyML (v3) [52] available at Phylogeny.fr web server (http://www.phylogeny.fr/) [53], and the phylogeny tree was rendered by MEGA4 [54]. The DNA and encoded protein sequences encoded by the TEs are listed in the Additional file 2 and Additional file 3.

## Abbreviations

ATIRs: Asymmetric terminal inverted repeats; bp: Base pairs; CDD: Conserved Domain Database; dsDNA: Double-stranded DNA; EN: Endonuclease; EST: Expressed sequence tag(s); HTH: Helix-turn-helix; IS: Insertion sequence; LTR: Long terminal repeat; ORF: Open reading frame; RT: Reverse transcriptase; SR: Serine recombinase; TEs: Transposable elements; TIRs: Terminal inverted repeats; Tpase: Transposase; TPRT: Target site-primed reverse transcription; TSDs: Target site duplications; VI: Virus integrated; YR: Tyrosine recombinase

## Competing interests

The authors declare that they have no competing interests.

## Authors’ contributions

Both authors contributed to the initial discovery of the Fanzor proteins in eukaryotes and wrote the manuscript. WB designed and performed the studies. All authors read and approved the final manuscript.

## Supplementary Material

Additional file 1 ***Fanzor *families in eukaryotic genomes.** Click here for file

Additional file 2 **DNA sequences of *Fanzor *families or other elements.** Click here for file

Additional file 3 Fanzor protein and other proteins sequences. Click here for file

Additional file 4 Alignment of Fanzor protein and TnpB proteins for the phylogeny. Click here for file

Additional file 5 **Alignment of Fanzor-encoded *Mariner*-Tpases.** Click here for file

Additional file 6 Domains contains in PGv-1-2p protein. Click here for file

Additional file 7 Mariner-1_OLpv-2p contains the C-terminal catalytic domain of the restriction endonuclease EcoRII. Click here for file

Additional file 8 **Alignment of the 5**^′^-ends of *CRe*-*1*, *2*, *3* and those of other *Helitrons* (A) and the sequences of 15 *CRe*-*1*, *2*, *3* insertions (B) Click here for file

Additional file 9 **Alignment of the RepHel proteins of *Helitron1 and Helitorn2 *groups.** Click here for file

Additional file 10 Alignment of the Sola2-Tpases. Click here for file

Additional file 11 **Alignments of the ends of *DFa*-*1*, *2*, *3 *and *PPa*-*1*, *4***, ***5 *families.** Click here for file

Additional file 12 Fanzor1 protien in MuDr superfamily. Click here for file

Additional file 13 TSDs of four viral Fanzor1 families. Click here for file

Additional file 14 Crypton Tpase alignment. Click here for file

Additional file 15 Alignment of Harbinger Tpase. Click here for file

## References

[B1] Chandler M Mahillon J Craig NL, Craigie R, Gellert M, Lambowitz AM Insertion sequences revisited Mobile DNA II 2002 Washington, DC: American Society for Microbiology Press 305 366

[B2] Kapitonov VV Jurka J Rolling-circle transposons in eukaryotes Proc Natl Acad Sci USA 2001 98 8714 8719 10.1073/pnas.151269298 11447285 PMC37501

[B3] Goodwin TJ Butler MI Poulter RT Cryptons: a group of tyrosine-recombinase-encoding DNA transposons from pathogenic fungi Microbiology 2003 149 3099 3109 10.1099/mic.0.26529-0 14600222

[B4] Cappello J Handelsman K Lodish HF Sequence of Dictyostelium DIRS-1: an apparent retrotransposon with inverted terminal repeats and an internal circle junction sequence Cell 1985 43 105 115 10.1016/0092-8674(85)90016-9 2416457

[B5] Goodwin TJ Poulter RT The DIRS1 group of retrotransposons Mol Biol Evol 2001 18 2067 2082 10.1093/oxfordjournals.molbev.a003748 11606703

[B6] Smith MC Thorpe HM Diversity in the serine recombinases Mol Microbiol 2002 44 299 307 10.1046/j.1365-2958.2002.02891.x 11972771

[B7] Grindley NDF Craig NL, Craigie R, Gellert M, Lambowitz AM The movement of Tn3-like elements: transposition and cointegrate resolution Mobile DNA II 2002 Washington, DC: American Society for Microbiology Press 272 302

[B8] Robertson DS Mutator activity in maize: timing of its activation in ontogeny Science 1981 213 1515 1517 10.1126/science.213.4515.1515 17780881

[B9] Robertson D Differential activity of the maize mutator Mol Genet Genomics 1985 200 9 13 10.1007/BF00383305

[B10] May EW Craig NL Switching from cut-and-paste to replicative Tn7 transposition Science 1996 272 401 404 10.1126/science.272.5260.401 8602527

[B11] Tavakoli NP Derbyshire KM Tipping the balance between replicative and simple transposition EMBO J 2001 20 2923 2930 10.1093/emboj/20.11.2923 11387225 PMC125483

[B12] Luan DD Korman MH Jakubczak JL Eickbush TH Reverse transcription of R2Bm RNA is primed by a nick at the chromosomal target site: a mechanism for non-LTR retrotransposition Cell 1993 72 595 605 10.1016/0092-8674(93)90078-5 7679954

[B13] Barabas O Ronning DR Guynet C Hickman AB Ton-Hoang B Chandler M Dyda F Mechanism of IS200/IS605 family DNA transposases: activation and transposon-directed target site selection Cell 2008 132 208 220 10.1016/j.cell.2007.12.029 18243097 PMC2680152

[B14] Kersulyte D Mukhopadhyay AK Shirai M Nakazawa T Berg DE Functional organization and insertion specificity of IS607, a chimeric element of Helicobacter pylori J Bacteriol 2000 182 5300 5308 10.1128/JB.182.19.5300-5308.2000 10986230 PMC110970

[B15] Kersulyte D Velapatino B Dailide G Mukhopadhyay AK Ito Y Cahuayme L Parkinson AJ Gilman RH Berg DE Transposable element ISHp608 of helicobacter pylori: nonrandom geographic distribution, functional organization, and insertion specificity J Bacteriol 2002 184 992 1002 10.1128/jb.184.4.992-1002.2002 11807059 PMC134827

[B16] Pasternak C Ton-Hoang B Coste G Bailone A Chandler M Sommer S Irradiation-induced deinococcus radiodurans genome fragmentation triggers transposition of a single resident insertion sequence PLoS Genet 2010 6 e1000799 10.1371/journal.pgen.1000799 20090938 PMC2806898

[B17] Siguier P Perochon J Lestrade L Mahillon J Chandler M ISfinder: the reference centre for bacterial insertion sequences Nucleic Acids Res 2006 34 D32 D36 10.1093/nar/gkj014 16381877 PMC1347377

[B18] Marchler-Bauer A Lu S Anderson JB Chitsaz F Derbyshire MK DeWeese-Scott C Fong JH Geer LY Geer RC Gonzales NR Gwadz M Hurwitz DI Jackson JD Ke Z Lanczycki CJ Lu F Marchler GH Mullokandov M Omelchenko MV Robertson CL Song JS Thanki N Yamashita RA Zhang D Zhang N Zheng C Bryant SH CDD: a conserved domain database for the functional annotation of proteins Nucleic Acids Res 2011 39 D225 D229 10.1093/nar/gkq1189 21109532 PMC3013737

[B19] Bernales I Mendiola MV de la Cruz F Intramolecular transposition of insertion sequence IS91 results in second-site simple insertions Mol Microbiol 1999 33 223 234 10.1046/j.1365-2958.1999.01432.x 10411740

[B20] Mendiola MV Bernales I de la Cruz F Differential roles of the transposon termini in IS91 transposition Proc Natl Acad Sci USA 1994 91 1922 1926 10.1073/pnas.91.5.1922 8127907 PMC43276

[B21] Kapitonov VV Jurka J Helitron-N1_SP, a family of autonomous helitrons in the sea urchin genome Repbase Reports 2005 5 394 394

[B22] Kapitonov VV Jurka J RPA70-Encoding helitrons in zebrafish Repbase Reports 2007 7 1179 1179

[B23] Yang HP Barbash DA Abundant and species-specific DINE-1 transposable elements in 12 drosophila genomes Genome Biol 2008 9 R39 10.1186/gb-2008-9-2-r39 18291035 PMC2374699

[B24] Coates BS Sumerford DV Hellmich RL Lewis LC A helitron-like transposon superfamily from lepidoptera disrupts (GAAA)(n) microsatellites and is responsible for flanking sequence similarity within a microsatellite family J Mol Evol 2010 70 275 288 10.1007/s00239-010-9330-6 20217059

[B25] Du C Caronna J He L Dooner HK Computational prediction and molecular confirmation of helitron transposons in the maize genome BMC Genomics 2008 9 51 10.1186/1471-2164-9-51 18226261 PMC2267711

[B26] Yang L Bennetzen JL Structure-based discovery and description of plant and animal helitrons Proc Natl Acad Sci USA 2009 106 12832 12837 10.1073/pnas.0905563106 19622734 PMC2722332

[B27] Dunin-Horkawicz S Feder M Bujnicki JM Phylogenomic analysis of the GIY-YIG nuclease superfamily BMC Genomics 2006 7 98 10.1186/1471-2164-7-98 16646971 PMC1564403

[B28] Roberts RJ Vincze T Posfai J Macelis D REBASE–a database for DNA restriction and modification: enzymes, genes and genomes Nucleic Acids Res 2010 38 D234 D236 10.1093/nar/gkp874 19846593 PMC2808884

[B29] Cock JM Sterck L Rouze P Scornet D Allen AE Amoutzias G Anthouard V Artiguenave F Aury JM Badger JH Beszteri B Billiau K Bonnet E Bothwell JH Bowler C Boyen C Brownlee C Carrano CJ Charrier B Cho GY Coelho SM Collén J Corre E Da Silva C Delage L Delaroque N Dittami SM Doulbeau S Elias M Farnham G Gachon CM Gschloessl B Heesch S Jabbari K Jubin C The Ectocarpus genome and the independent evolution of multicellularity in brown algae Nature 2010 465 617 621 10.1038/nature09016 20520714

[B30] Bao W Jurka MG Kapitonov VV Jurka J New superfamilies of eukaryotic DNA transposons and their internal divisions Mol Biol Evol 2009 26 983 993 10.1093/molbev/msp013 19174482 PMC2727372

[B31] Yuan YW Wessler SR The catalytic domain of all eukaryotic cut-and-paste transposase superfamilies Proc Natl Acad Sci USA 2011 108 7884 7889 10.1073/pnas.1104208108 21518873 PMC3093488

[B32] Stanley TL Ellermeier CD Slauch JM Tissue-specific gene expression identifies a gene in the lysogenic phage gifsy-1 that affects salmonella enterica serovar typhimurium survival in Peyer’s patches J Bacteriol 2000 182 4406 4413 10.1128/JB.182.16.4406-4413.2000 10913072 PMC94610

[B33] Peters JE Craig NL Tn7: smarter than we thought Nat Rev Mol Cell Biol 2001 2 806 814 10.1038/35099006 11715047

[B34] Arkhipova IR Batzer MA Brosius J Feschotte C Moran JV Schmitz J Jurka J Genomic impact of eukaryotic transposable elements MDNA 2012 3 19 10.1186/1759-8753-3-19 PMC3520738 23171443

[B35] Bao W Jurka J Origin and evolution of LINE-1 derived “half-L1” retrotransposons (HAL1) Gene 2010 465 9 16 10.1016/j.gene.2010.06.005 20600705 PMC2923044

[B36] Hohjoh H Singer MF Cytoplasmic ribonucleoprotein complexes containing human LINE-1 protein and RNA EMBO J 1996 15 630 639 8599946 PMC449981

[B37] Martin SL Bushman FD Nucleic acid chaperone activity of the ORF1 protein from the mouse LINE-1 retrotransposon Mol Cell Biol 2001 21 467 475 10.1128/MCB.21.2.467-475.2001 11134335 PMC86601

[B38] Bender J Cytosine methylation of repeated sequences in eukaryotes: the role of DNA pairing Trends Biochem Sci 1998 23 252 256 10.1016/S0968-0004(98)01225-0 9697415

[B39] Zhou Y Cambareri EB Kinsey JA DNA methylation inhibits expression and transposition of the neurospora Tad retrotransposon Mol Genet Genomics 2001 265 748 754 10.1007/s004380100472 11459196

[B40] Roberts D Hoopes BC McClure WR Kleckner N IS10 transposition is regulated by DNA adenine methylation Cell 1985 43 117 130 10.1016/0092-8674(85)90017-0 3000598

[B41] Reznikoff WS The Tn5 transposon Annu Rev Microbiol 1993 47 945 963 10.1146/annurev.mi.47.100193.004501 7504907

[B42] Yang MK Ser SC Lee CH Involvement of E. coli dcm methylase in Tn3 transposition Proc Natl Sci Counc Repub China B 1989 13 276 283 2561572

[B43] Low DA Weyand NJ Mahan MJ Roles of DNA adenine methylation in regulating bacterial gene expression and virulence Infect Immun 2001 69 7197 7204 10.1128/IAI.69.12.7197-7204.2001 11705888 PMC98802

[B44] Heusipp G Falker S Schmidt MA DNA adenine methylation and bacterial pathogenesis Int J Med Microbiol 2007 297 1 7 17126598 10.1016/j.ijmm.2006.10.002

[B45] Giacomodonato MN Sarnacki SH Llana MN Cerquetti MC Dam and its role in pathogenicity of salmonella enterica J Infect Dev Ctries 2009 3 484 490 19762965 10.3855/jidc.465

[B46] Yau S Lauro FM DeMaere MZ Brown MV Thomas T Raftery MJ Andrews-Pfannkoch C Lewis M Hoffman JM Gibson JA Cavicchioli R Virophage control of antarctic algal host-virus dynamics Proc Natl Acad Sci USA 2011 108 6163 6168 10.1073/pnas.1018221108 21444812 PMC3076838

[B47] Koonin EV Makarova KS Aravind L Horizontal gene transfer in prokaryotes: quantification and classification Annu Rev Microbiol 2001 55 709 742 10.1146/annurev.micro.55.1.709 11544372 PMC4781227

[B48] Keeling PJ Palmer JD Horizontal gene transfer in eukaryotic evolution Nat Rev Genet 2008 9 605 618 10.1038/nrg2386 18591983

[B49] Bao Z Eddy SR Automated de novo identification of repeat sequence families in sequenced genomes Genome Res 2002 12 1269 1276 10.1101/gr.88502 12176934 PMC186642

[B50] Jurka J Kapitonov VV Pavlicek A Klonowski P Kohany O Walichiewicz J Repbase update, a database of eukaryotic repetitive elements Cytogenet Genome Res 2005 110 462 467 10.1159/000084979 16093699

[B51] Katoh K Kuma K Toh H Miyata T MAFFT version 5: improvement in accuracy of multiple sequence alignment Nucleic Acids Res 2005 33 511 518 10.1093/nar/gki198 15661851 PMC548345

[B52] Guindon S Dufayard JF Lefort V Anisimova M Hordijk W Gascuel O New algorithms and methods to estimate maximum-likelihood phylogenies: assessing the performance of PhyML 3.0 Syst Biol 2010 59 307 321 10.1093/sysbio/syq010 20525638

[B53] Dereeper A Guignon V Blanc G Audic S Buffet S Chevenet F Dufayard JF Guindon S Lefort V Lescot M Claverie JM Gascuel O Phylogeny.fr: robust phylogenetic analysis for the non-specialist Nucleic Acids Res 2008 36 W465 W469 10.1093/nar/gkn180 18424797 PMC2447785

[B54] Tamura K Dudley J Nei M Kumar S MEGA4: molecular evolutionary genetics analysis (MEGA) software version 4.0 Mol Biol Evol 2007 24 1596 1599 10.1093/molbev/msm092 17488738

