# Supplementary material for: Homologues of bacterial TnpB_IS605 are widespread in diverse eukaryotic transposable elements
Source: Mob DNA. 2013 Apr 1;4:12. doi: 10.1186/1759-8753-4-12 (PMC3627910; doi:10.1186/1759-8753-4-12)
Supplement: Additional file 1 — Fanzor families in eukaryotic genomes. [file 1759-8753-4-12-S1.docx]

**Additonal file 1**

**Table 1. *Fanzor* families in eukaryotics genomes**

| Family (bp) | Copy No. | Termini | TIR  (bp) | TSD  (bp) | Fanzor protein (aa) &(No. Exons) | Tpase ^#^  (Superfamily) | Comments |
| --- | --- | --- | --- | --- | --- | --- | --- |
| *MDe-1* | 2 |  |  |  | 815 (3) |  |  |
| *MDe-2* | 2 |  |  |  | 698 (4) |  |  |
| *MDe-3* | 1 |  |  |  | 620 (4) |  |  |
| *MDe-4* | 4 | L.R. | N | n.a. | 731 (4) |  |  |
| *MDe-5* | 4 | L.R. | N | n.a. | 656 (4) |  |  |
| *MDe-6* (3852) | 10 | L.R. | N | n.a. | 661 (4) |  |  |
| *MDe-7* (3937) | 8 | L.R. | 24 | 2 (TA) | 772 (3) |  |  |
| *MDe-8* | 4 | R. |  |  | 745 (3) |  |  |
| *MDe-9* | 3 | R. |  |  | 764 (5) |  |  |
| *MDe-10* | 1 |  |  |  | 779 (3) |  |  |
| *MDe-11* | 3 | R. |  |  | 713 (4) |  |  |
| *MDe-12* (3875) | 5 | L.R. | N | n.a. | 677 (4) |  |  |
| *MDe-13* | 3 | R. |  |  | 680 (2) |  |  |
| *HMa-1* | 1 |  |  |  | i.c. | Mariner | Probably from virus |
| *SAl-1** | 3 | R. |  |  | 400 (1) |  |  |
| *SAl-2** | 3 | R. |  |  | 498 (4) |  |  |
| *SPu-1* (2149) | 25 | L.R. | 33 | 2 (TA) | 633 (1) |  |  |
| *SPu-2* | 2 |  |  |  | 663 (1) |  |  |
| *SPu-3* (2288) | 2 | L.R. | 25 | 2 (TA) | 626 (1) |  |  |
| *ROr-1* (5190) | 10 | L.R. | 90 | 2 (TA) | 928 (3) | Mariner |  |
| *ROr-2* (4073) | 18 | L.R. | 46 | 2 (TA) | 690 (2) | Mariner |  |
| *ROr-3* (2862) | 16 | L.R. | 133 | 2 (TA) | 720 (2) |  |  |
| *ROr-4* (5244) | 9 | L.R. | 38 | 9 | 1165 (3) | (MuDr) |  |
| *AMa-1* | 1 |  |  |  | 871 (4) |  |  |
| *AMa-2* | 1 |  |  |  | 645 (3) |  |  |
| *AMa-3* | 1 |  |  |  | 789 (7) |  |  |
| *PBl-1* (3938) | 4 | L.R. | 12 | 3 (TAN) | 683 (4) |  |  |
| *PBl-2* | 3 |  |  |  | 677 (2) |  |  |
| *PBl-3* (4614) | 6 | L.R. | 42 | 9 | 1186 (3) | (MuDr) |  |
| *MCi-1* (4036) | 4 | L.R. | 20 | 2 (TA) | 686 (2) | Mariner |  |
| *MCi-2A* (10235) | 3 | L.R. | N | 11 | 1375 (4) | Crypton |  |
| *MCi-2B* | 2 | R. | - | - | 1375 (4) |  |  |
| *MCi-2C* | 3 | R. | - | - | 1375 (4) |  |  |
| *MCi-2D* (9295) | 2 | L.R. | N | 12 | 1375 (4) |  |  |
| *MCi-3* (5305) | 2 | L.R. | 39 | 4? (TTAA) | 1304 (2) |  |  |
| *MCi-4* (4508) | 6 | L.R. | 31 | 9 | 1245 (3) | (MuDr) |  |
| *MCi-5* (7323) | 5 | L.R. | N | n.a. | 1212 (3) | Harbinger |  |
| *MCi-6* | 2 |  |  |  | 1231 (2) |  |  |
| *MCi-7* | 1 | R. |  |  | 1153 (3) |  |  |
| *MCi-8* | 1 |  |  |  | 1067 (2) |  |  |
| *MCi-9* | 1 |  |  |  | 1149 (3) |  |  |
| *MCi-10* | 1 |  |  |  | 1135 (4) |  |  |
| *AGo-1** | 1 |  |  |  | 457 (1) |  |  |
| *ECy-1** | 1 |  |  |  | 455 (1) |  |  |
| *SCe-1** | 1 |  |  |  | 350 (1) |  |  |
| *TDe-1** (1785) | 7 | L.R. |  |  | 486 (1) |  |  |
| *DFa-1* (11949) | 12 | L.R. | 12 | 4 | 1241 (10) | (Sola2) |  |
| *DFa-2* (12887) | 7 | L.R. | 12 | 4 | 1010 (9) | Sola2 |  |
| *DFa-3* (10254) | 2 | L.R. | 13 | 4 | 1084 (10) | (Sola2) |  |
| *DFa-4* | 1 |  |  |  | 1020 (13) | - |  |
| *PPa-1* (13566) | 3 | L.R. | 22 | 4 | 1699 (7) | Sola2 |  |
| *PPa-2* | 1 |  |  |  | 945 (8) |  |  |
| *PPa-3* | 1 |  |  |  | 970 (9) |  |  |
| *PPa-4* (14423) | 3 | L.R. | 16 | 4 | 1827 (14) | Sola2 |  |
| *PPa-5* (15292) | 3 | L.R. | 16 | 4 | 1388 (12) | Sola2 |  |
| *PPa-6* | 2 | R. | 16 | 4 | 1218 (13) |  |  |
| *PPa-7* | 1 |  |  |  | 1756 (16) |  |  |
| *ACa-1** (2675) | 2 | L.R. | N | 0 | 603 (1) | TnpA_IS607 |  |
| *ACa-2** | 1 |  |  |  | 653 (1) | TnpA_IS607 |  |
| *VCa-1* | 1 |  |  |  | 768 (1) |  |  |
| *VCa-2* | 1 |  |  |  | i.c. |  |  |
| *CRe-1* (3992) | >100 | L.R. | N | 0 or n | 830 (5) | (Helitron) | Expressed |
| *CRe-2* (4882) | >100 | L.R. | N | 0 or n | 906 (10) | (Helitron) | Expressed |
| *CRe-3* (4688) | >100 | L.R. | N | 0 or n | 967 (10) | (Helitron) | Expressed |
| *CRe-4* | 3 | R. |  |  | 944 (6) |  |  |
| *CRe-5* | 3 | R. |  |  | i.c. |  |  |
| *CVu-1* | n.a |  |  |  | i.c. |  |  |
| *CMe-1A* (3169) | 150 | L.R. | N | n.a. | 734 (1) |  |  |
| *PUl-1* (3620) | 8 | L.R. | 24 | 2 (TA) | 802 (1) | Mariner |  |
| *PUl-2* (3820) | 1 | L.R. | 33 | 2 (TA) | 643 (3) | Mariner |  |
| *PUl-3* | 1 |  |  |  | 799 (1) |  |  |
| *PUl-4* (3356) | 3 | L.R. | 26 | 2 (TA) | 809 (1) |  |  |
| *PUl-5* | 1 | R. |  |  | 617 (1) |  |  |
| *PUl-6* | 5 | R. |  |  | 642 (1) |  |  |
| *NOc-1* | 4 |  |  |  | i.c. |  |  |
| *PSo-1* | 2 | R. |  |  | 660 (1) |  |  |
| *PSo-2* | 4 | R |  |  | 726 (1) |  |  |
| *PSo-3* | 3 |  |  |  | 716 (1) |  |  |
| *PSo-4* | 3 |  |  |  | 785 |  |  |
| *PSo-5** | 1 |  |  |  | i.c. |  |  |
| *PCa-1,* | 2 | R. |  |  | 788 (1) |  |  |
| *PCa-2* (2107) | 2 | L.R. | N | N | 611 (1) |  |  |
| *PCa-3** | 2 | R. |  |  | 483 |  |  |
| *PRa-1* | 1 |  |  |  | i.c. |  |  |
| *PRa-2** | 2 | R. |  |  | i.c. |  |  |
| *ALa-1* | 1 |  |  |  | i.c. |  |  |
| *ALa-2* | 1 |  |  |  | i.c. |  |  |
| *ESvi-1A* (3180) | 1 | L.R. | 59 |  | 890 (1) |  |  |
| *ESvi-1B* (4052) | 1 | L.R. | 25 | 8 | 890 (1) | IS4 |  |
| *ESv-1* (2639) | 2 | L.R. | 40 | 2 (TA) | 693 (1) |  |  |
| *ESv-2* (3603) | 2 | L.R. | 18 |  | 757 (1) | IS4 |  |
| *SWv-1* (2633) | 1 | L.R. | 21 | 6 | 779 (1) |  |  |
| *HAgv-1* (1963) | 2 | L.R. | 13 | 4 (TTAT) | 572 (1) |  |  |
| *HAmv-1* (1925) | 1 | L.R. | 13 | 4 (TTAA) | 592 (1) |  |  |
| *PUgv-1* (1961) | 2 | L.R. | 13 | 4 (TTAT) | 571 (1) |  |  |
| *SFav-1* (1954) | 2 | L.R. | 13 | 4 (TTAN) | 606 (1) |  |  |
| *HVav-1* (1955) | 5 | L.R. | 13 | 4 (TTAN) | 608 (1) |  |  |
| *MCnv-1* | 1 | R. |  |  | i.c. |  |  |
| *PGv-1* (4442) | 1 | L.R. | 29 | 2 (TA) | 625 (1) | Mariner |  |
| *EHv88-1* | 1 |  |  |  | 650 (1) |  |  |
| *EHv99B1-1** (2126) | 1 | L.R. |  |  | 640 (1) |  |  |
| *ISvMimi_1** (2549) | 3 | L.R. |  |  | 520 (1) | TnpA_IS607 | *=APmv-2, =ACmv-2* |
| *ISvMimi_2** | 1 |  |  |  | 545 (1) | TnpA_IS607 | *=APmv-1, =ACmv-1* |
| *APmv-3** | 1 |  |  |  | 482 (1) |  | =*ACmv-3* |
| *MGvc-1*,* | 1 |  |  |  | 526 (1) |  |  |
| *MGvc-2** | 1 |  |  |  | 493 (1) |  |  |
| *ISvAR158_1** | 1 |  |  |  | 351 (1) | TnpA_IS607 |  |
| *ISvNY2A_1** (2164) | 3 | L.R. |  |  | 395 (1) | TnpA_IS607 |  |
| *ISvNY2A_2** (1443) | 2 | L.R. |  |  | 432 (1) |  |  |
| *CRv-1** | 1 |  |  |  | 416 (1) | TnpA_IS607 |  |
| *FEsv-1** | 1 |  |  |  | 408 (1) |  |  |

*Fanzor* elements are named after the host species (see Table 1). *Fanzor2* elements are indicated by *. The left and right termini are indicated by L. and R. respectively, in the orientation of the encoded *Fanzor* protein. N: none; n.a.: not available; i.c.: incomplete. ^#^: The encoded Tpase (or coding sequences). If a given *Fanzor* element does not encode Tpase, but the superfamily it belongs can be determined, the superfamily name is parenthesized.
