# Supplementary material for: Homologues of bacterial TnpB_IS605 are widespread in diverse eukaryotic transposable elements
Source: Mob DNA. 2013 Apr 1;4:12. doi: 10.1186/1759-8753-4-12 (PMC3627910; doi:10.1186/1759-8753-4-12)
Supplement: Additional file 5 — Alignment of Fanzor-encoded Mariner-Tpases. [file 1759-8753-4-12-S5.pdf]

Additional file 5

```
                          10        20        30        40        50        60        70        80        90       100       110
PUl-1          M--------------------------------------------------------------------------------RAQADRNACFRLRSKGFT----LAQIAHA
PUl-2          M--------------------------------------------------------------------------------HSPEERKICCRLRTQDIP----LTVIATA
PGv-1          M----------------------------------------------------------------------------SIRKSNDYKLTAVNHYLVEDKS----QEEICKI
HMa-1          --------------------------------------------------------------------------------------------------------------
ROr-1          MTKNTFFYEDGHDNVYNKDGERVFDPMEGELTEISDPNVVLETITSQKSYLSLKPAEKETTAKDSTKKAIEVVKKSSVYKDYNDQTREIFINRMIEGPTERGKVTLHAKD
ROr-2          --------------------------------------------------------------------------------------------------------------
MCi-1          M--------------------------------------------EKMFHKCHLINSPGLYFSQYLIHFQLKSTEISNMKHLTCCEKAYIHGLNDAGMQ----VPEIAIR
SMAR30         MKIDENISLDKSENQLNNTTSNEQNFANQQIRSIFQQQAELDL-----------PNPLTAETNSDTAVIQIRRYNRSAYNTTSLDKKKIFIKCYEEGKT----IVEAAEI
Mariner-2_MLP  M-----------------------------------------------------CEGISPLPSKFLEQLLIIVSQKMKTRCLEPVERARIIGMHKAGLS----GTKISKK
Mariner-1_PGr  M----------------------------------------------------------------------VRTKPDRKPHLSTETKAMIVGMARAGKS----LAYLSQE
IS630          M---------------------------------------------------------PIIAPISRDERRLMQKAIHKTHDKNYARRLTAMLMLHRGDR----VSDVART
ISSoc15        M-----------------------------------------------------------------------------PTHSLDLRQRVVAAYQAGNTS----IRQVAKR

                         120       130       140       150       160       170       180       190       200       210       220
PUl-1          LDICARTVQRWLKGES-----AAAR-PLNRNHTRKLTSLQEERLIEFFLD-------ENTSRLRDGVAFVAQSF-NLCVSERTLGRILHRH-RLSFKRAVSAYHEA----
PUl-2          LNVCKRTVQRWLKSTP-----IVSR-PLNRSHLRKLNETQREKVIEYFLN-------NNTSRIRDGVEFVRNAF-AIFVSRSTIQRVLHES-QLTFKKASTAYKEA----
PGv-1          FKCSRRSLMRWVDRYEKDG--NVDI-HYRKPIAYKVKKEHVAFLLQEMKK-------NKTITIEDLLYLLKNKYPDVDLNKSHINRIIKDN-NITLKITRIRHEPVKRFG
HMa-1          --------------------------------------------------------------------------------------------------------------
ROr-1          LGINPRTAMRWWKHYQETGKVAYKKLQRNPGRPNSLTPEHEQHIQQIVEK-------DSQLCADDIIDSLKSQFEDLKILKSQMNHHLRNNILISIKKPTYDPMTR----
ROr-2          --------------------------------------------------------------------------------------------------------------
MCi-1          MGRSESAIYALFKRENDDS--SEEN-KNPVGRPQEYTERDKRLAVRYAKK-------NRRASLSEIANAGYKKV-GKGVVRRALYEAGLHK-RVAIKKPYLRIEQV----
SMAR30         SGINKYTAASLLKRYKNDGCLILQK-KRGGKRTAKITSEIMSAIEDIVEQ-------NPAITLKSISKKIMDEK-HINLTTTSINNALKRL-RITLKTATLNLDRL----
Mariner-2_MLP  LHHPRPTVYTVISKFKRTN--TFSS-SKASGRPKKLTDQDRRSLVRQAKK-------DRRAPLANIASNLPTQV-GCTTVRSELHKLGFHS-CVAVAKPYLTPSHI----
Mariner-1_PGr  FGTARSTLSGIINRWKDRG--TNES-KPPTGRPPLTTERDERRLRQVLDD-------QRRAPMREIVEQLATPI-SIRTARRRAHKLGYQN-RRAVKKPFVNDKQA----
IS630          LCCARSSVGRWINWFTQSGVEGLKS-LPAGRARRWPFEHICTLLRELVKHSPGDFGYQRSRWSTELLAIKINEITGCQLNAGTVRRWLPSA-GIVWRRAAPTLRIR----
ISSoc15        FMVTKRTVHRWVRQYQQTQ--DLAP-KKAGTKRVGILEQHRQEVMAIITE-------HPDFYLWQYQELLRERL-GINVSIVTIHNFLKKQ-GMTLKKKTYRSAKV----

                         230       240       250       260       270       280       290       300       310       320       330
PUl-1          ----DRARQQVFLANLPRDS---TKSWIAL-DEAAFFLNHSRKYAWSKRGTPAVVD----RPSNR-GRMHS-LILTTSPDGVVDWRLIQGS-VDSLR-------------
PUl-2          ----DLARQKEFLSTLSQHA---NKHWIAL-DEAAFFLNHSRKYAWSERGKPAVVR----KPGRR-GKMYS-LILATSPEGVIKWRLVQGS-INSTR-------------
PGv-1          KDIDINANIKKFYEELKKYK---LEDIICI-DETSIKSLQKRHHCYSEKGKRCVIK----TQSQEVFKKYT-GIFAISVNGVEGWDLYEKSGINTDR-------------
HMa-1          ----------------------------------KYKWSSKRHQCYSELGKRCVIK----TQSQEVFKKYT-GIFAISYNGVLGWDLYEKDGINSDR-------------
ROr-1          NSDNNLQTRYEWFMKWKGSDLGYTKNCVFI-DEAAKQKKKESLQESKGRAGDIMIE----EPTIE-------YVDVEESTAIENNKSVAKG-TTTAH-------------
ROr-2          --------------------------------------------------------------------------------------------------------------
MCi-1          ------RSRRAFAEECKGWTMEQWKKVIWT-DEASFELRKPYTRITVWRTCDKAYKKECHSYHEPFTLRMHGNFMLFSMQHVLGKTLHTRSLQDQIRKSTQRHXXRGIFR
SMAR30         NSPSTILQRKEYALNFSLNAPIIREKIVFI-DESGFNCHLRRTKARSKINTGAHVI----IPTLR-GRNVS-LIAAMNLHGIVHKQIIANSTVNANV-------------
Mariner-2_MLP  ------TKRLKFAKKHKNWTVDDWKKVIWT-DESSFEIGKNSRQVQVWRKAGEKYNKDCLTPTFKSGRSSTMIWGAFTANSRLDMIFMEPGKQTGSD-------------
Mariner-1_PGr  ------ARRLAFATAHLNWTVDDWRSVLWT-DESSFELGKNSKAISVWRQTDEKYRPECLEPTFRSGRSSTMIWGGFFDKTKAPVLFMPPGQRNAAS-------------
IS630          ----DPHKDEKMAAIHKALDECSAEHPVFYEDEVDIHLNPKIGADWQLRGQQKRVV----TP----GQNEKYYLAGALHSGTGKVSCVGGNSKSSAL-------------
ISSoc15        KEEEVQRERLAYSQEVRNIP---AEDLIAI-DQTGVWEGMERRVSRSLRGQRAYHY----RQRYK-GEKYT-VIGAISLRGVVGCRVIKGG-MKKGD-------------

                         340       350       360       370       380       390       400       410       420       430       440
PUl-1          --FHAFL--SGL---------PGDTNVLLD------------------------------------------------NCATHHASHVLVKQGKTTVSQVARSK-QMTLN
PUl-2          --FHSFL--SEL---------PNDTNVLLD------------------------------------------------NVSTHKATHSLRKQDLSTIPEVAESK-RINML
PGv-1          --LVEFL--EKHIT-----ENYKNKLIILD------------------------------------------------NASSHRNQRI--K-------DLV-NK-HNNIL
HMa-1          --LYEFL--EKHITKK-IQKKYKNKLIFLD------------------------------------------------NASSHRNEKI--K-------ELL-NK-HNTLL
ROr-1          --FVRFI--SELLDIMNMDESLMGSYLVMG------------------------------------------------YCNIHKSHPM--I-------RKIESR-GYRVM
ROr-2          --YYNFI--ANALDILYQXEXLKSSYIVMD------------------------------------------------NAPIHKNADIRKQ-------KIIEQR-GYGCV
MCi-1          LIFHAIFTINXILGRLLTLGQHNTKFIFKDRXTTEPRWPFVCHHYHILINQXQDDLARSHSIICLFDFCQKKKKKKGINTMVFHSIFFKSN-------TLFYTN-ISRIL
SMAR30         --FINFL--DGLFQTL-REANITCAWLVLD------------------------------------------------NCTIHKTQEV--R-------DKVGTT-SHTLV
Mariner-2_MLP  --FVKQVYEGSLGPFLNSPTTPSGLILMED------------------------------------------------GAPVHRCKVA--Q-------SWRDMH-PISKL
Mariner-1_PGr  --FIENVYEKGLQPFMDGIDEAHRPTLMED------------------------------------------------NAPVHTAILS--R-------NYRASH-GIKKI
IS630          --FISLL--KRLKA---TYRRAKTITLIVD------------------------------------------------NYIIHKSRET--Q-------SWLKENPKFRVI
ISSoc15        --FLEFL--RSEL----CPKLDARKVVIMD------------------------------------------------NLNIHKSREV--E-------ELIRGT-GARIL

                         450       460       470       480       490       500       510       520
PUl-1          -YLPPYSPQLNPVELCFNLIRTHVNRT--APRTLGSLHKCIQTAIRGLTPAVCRSMFRKCLLIG------------------
PUl-2          -YLPPYSPQLNPVELCFNFIRTSVNGS--APRTLESLRSSIQTAIEALTPSVCRSMFARTLKICRL----------------
PGv-1          -YAVPYQHFTNSIENYFSMLKSRLQKL--DGLTHTKLKENIENVISKIPKDKYKNIFKGAYERPEKYIPK-NKTRKVKKDYL
HMa-1          -YSVPYQHFTNSIENYFSMMKSRLQKL--DGLTHKELKTNIKKVIKDIPKEKYENIIKGTYNRTEKYHKKLSNRRKTLKNYF
ROr-1          -YLPPCSPELNPIEQ-------------------------------------------------------------------
ROr-2          -YLPAYSPELNPIGQFXSVCKSKLKRK--ELLQEETLSSRSEDACNRILLSDLQGFCRYSAARFEDCLNR---------KPM
MCi-1          -LLP--SPILXGTYKHCCNLRST-----------------------------------------------------------
SMAR30         -YLPPYSPMLNPIEKVFSKTKLCARNLLAESRTDLNLENVIQESLATVTQSDCNNYYVDMTMKLPLA--------TGGQPLH
Mariner-2_MLP  -EWPANSPDLNPIENIRRDMKMAVSTK-HQPRTVSEMKTVLNLEWNSIPDSKLESLVASMPARMQAVIEA-----KGGHTKW
Mariner-1_PGr  DNWPAQSPDLNPIENVWKDLKVAIQTL-YNPRSIPELKEAISQAWDDYPTQTFDHLLESMPHRMKAVVDA-----DGGPTRW
IS630          -YQPVYSPWVNHVERLWQALHDTITRN-HQCSSMWQLLKKVRHFMETVSP---------------------FPGGKHGLAKV
ISSoc15        -YLPVYAPELNPIEMMWSVLKHFIRQL--CRIGKYSMEQIVKTSLLLINPSSFRSWFAKCCYCTP-----------------
```
