# Supplementary material for: Homologues of bacterial TnpB_IS605 are widespread in diverse eukaryotic transposable elements
Source: Mob DNA. 2013 Apr 1;4:12. doi: 10.1186/1759-8753-4-12 (PMC3627910; doi:10.1186/1759-8753-4-12)
Supplement: Additional file 6 — Domains contains in PGv-1-2p protein. [file 1759-8753-4-12-S6.pdf]

Additional file 6

PGv-1-2p
(291-aa)

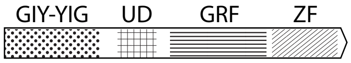

GIY-YIG

```
                           *               * *        *   *
PGv-1-2p               MRWIYILKC-----ED-NYFYVGETAR-LYRRFWEHTAGIGGLNTQIFKPEEIVAIY--KVDTIGKFLEYN
AACY021487243.1      MPCTWLYVLTS-----NDGDYVYVGMTHR-LITRLNEHMEGKGAVATSRRIYNTVQCVY--KIDTVEQHNHAL
AACY022610711.1        MHWVYILKC-----NN-NNLYVGETKT-LYKRLNQHVNKKGSKHTCDGTLESLNALY--KVQSLYRYLMYC
AACY023304359.1       MVHWIYVLEC-----SD-RNIYVGETTR-LYSRFSEHIGGRGSVNTSSHSPRKLIGLY--KVDDNHSFFKYR
1YWL              MENKKSHYFYVLLC-----QD-GSFYGGYTTE-PERRLTEHNSGTGAKYTRLAKRRPVIMIHTEKFETRSEATKAE
YP_003786767.1     MENEFYYVYILLC-----ED-GSYYTGVTNN-LKERFIKHSKGKGAKYTKTHKPIKFLSAW--RVENISIALKIE
Penelope-5_NV_1p  -TCNTKNVIYMIQC-----NRCNLQYIGETKRKLKERFNDHRRTVDSQSRSIPT---HAAEH--FLKPNHSASDIE
Penelope-1_OL_1p  -SYKQSNVVYSIHC----KENCNEQYIGETKQPLHKRLYQHRRANPSG------PESAVHLH--LKATNHSFEDSE
Neptune2_Ren_2p   -TCTSSNVIYLITC-----LKCQKQYVGKTSKTLRERICHHRSSINNNEPR------YISKH--FNLPGHQLSHLK
Penelope2_Dw_1p   -TMDKSNVVYKIPCNGTNEESCDKMYIGTTKSRLKTRLSQHRSDYKLRQHSNIQ-KTALMAH--CAASGHSPNFDE
```

UD

```
PGv-1-2p          CAKYKDYFDKLEAENNIAECM---------------MIHNKDNWEKIRGGKYTKFNC
AACY023304359.1   GEGSGN----LDIENHITELYFHLRNKEDDIEGVSSFRYNDGMTNKVNGGKYTKMFN
AACY022610711.1   VWNNKD--WAIKVENFITEYL----------------LEE---NISVNGGKYVNDNK
AACY022686916.1   IWNNKQ--YALEIENFITECI----------------LST---NENTFGGKYVNSNR
AACY020070058.1   MEGSKD--KAEEIENYVTEFI----------------MSSVDDNKMVRGGKYMKGEK
AACY021487243.1   ---------NHALENELTLKIM---------------RSRGGAWWKVRGGKWCGMTK
```

GRF

```
PGv-1-2p                    PINNNIKELP-MCKCGVPCD--IKKNDDKNY--LFFRCAKKNM-WENFKDDFDI--EDEPCNFFMEYITDIEFRIDE
AACY023293758.1             PNNQLAKELP-NCNCGFPCD--VKQNEENNY--LYFRCAKKNM-WDKMREEFGI--ADEPCKFFMKYTNDNKYKIEY
AACY023304359.1             VDNDYILDRP-CCDCMFPCE--VKISKDKKT--IYFVCSVKNI-WDDFDSKGLQ--IEGPCDFYKVYTEDTYIKKAY
AACY023344224.1             IPETSIVDRP-LCKCLLPSE--VKLSKDKTK--IYFVCALKNV-WGDFFAD-LQ--IDEPCDFWQMYTEDNAAKAEY
AACY022610711.1             LKKV-----P-LCKCGLPAE--IKCVKNSKYYKLYFTCCIKNV-WSKMSSEFKKINIHNPCNYYFECMDGLEFRMIM
AACY022686916.1             NKKKSINNRP-ICKCGLPCE--VKIIKPGKYYKIYYVCSKKNI-WEHMRERFNHLNIPKCCNFYCEYLDDLEYRVKL
AACY020070058.1             GEKKEDDGRP-MCECGIPCE--IVKRQTKRKINFYYVCPLKNV-WDEMRKEFKMIEIEKPCKYFEEYMDDVEKRVMY
AACY021487243.1             PELRAIQGFPEMCLCHYPVD--VMVA---KNGRKFATCACKNTDWLRGKVNVGYEIAGSTCNYFRWTDGND------
XP_002428087.1-1GRF finger  SSSNNDLSIP-KCFCQLPAISRVVTKLGQNHGKLFYTCNNQNI--------------SNKCNFFQWALPDGQTGNHA
XP_002428087.1-2GRF finger  VSKTKDTNVV-KCHCSVSTKKCVVNKEGPNKGKSFFVCGNKNI--------------DGKCNFFKWA----------
XP_394050.4, 1GRF finger    WGDIDENSVI-LCNCNENAIQLIVRKEGPNHGRAFYKCA-KPM--------------NNRCDFFIWAPDNMSGHIQN
XP_394050.4, 2GRF finger    -NNYNVATIV-KCNCNQLATTRIVKKDGPNKGRSFYTCP-KNM--------------NESCKFFQWADENVEATCNN
EFN81542.1 1GRF finger      WGNIDNNAVI-MCNCHETAIQLTVRKEGPNIGRLFYKCA-KPQ--------------GSGCHFFLWASDSDESQANT
EFN81542.1 2GRF finger      WGSDASSNDV-ICQCNQPARKLTVHKEGPNKGRQFYGCP-KGI--------------NSSCNFFQWADENTDQSSFS
```

ZF

```
PGv-1-2p         KKSFWLNNVECEIDDECPE-CVSCNKYVWCDKNGEFKNNGIEYGNERRLLCRDCFVNKNEELSQKYSL---TGKCLIKL
AACY023293758.1  SKSHWLKELSGGQYEHCVGGC----------GKEYDQNNTIRYLRKAINLCFDCFIDKNEELAKKYNQNNPVGKCLIQLKHLM
AACY023304359.1  LKESWLSNIPVSLYKIHPEPCVMCKKTNYL---------AIFAFTKVRRICQECMINKYDELKKDYSSS--PGRCLMVD
AACY023344224.1  SRENWVLNIPSSRYKIHPDPCISCSKTDYL---------AIYN-NGIRRLCQSCIIKKYNDLKEKYDKS-----CLIKDT
```
