# Supplementary material for: Homologues of bacterial TnpB_IS605 are widespread in diverse eukaryotic transposable elements
Source: Mob DNA. 2013 Apr 1;4:12. doi: 10.1186/1759-8753-4-12 (PMC3627910; doi:10.1186/1759-8753-4-12)
Supplement: Additional file 7 — Mariner-1_OLpv-2p contains the C-terminal catalytic domain of the restriction endonuclease EcoRII. [file 1759-8753-4-12-S7.pdf]

Additional file 7

```
Mariner-1_OLpv-2p               M-----------------------------------------------------------------------------------------------------------------------
Q8G4B6_BIFLO Type II restricti  MSDYGR---------------IGDYIGPVA----------AKKLSAVD---IDANSSNQHEFGGNDALRRLLGTGEDRR----ASQGHGIPTAL--MYLSD-DDAPAVADLETTWYDARR
Q0AK57_MARMM Type II restricti  M------------------------------------------LAVVD---CVGHGSNQHEVTATGDLFEMLG--DQPRKLVRGSSDERFDAIY--VHVTE-TDDVIADTGKLSWYDSRA
Q727L4_DESVH Type II restricti  M--------------------RLSDLFCVA----------AKRLRAVE---AEPARSNQHEFNGVAALRNMLG--DDRR---------TLAARF--ILLGD-DESPLACDGSLTWYDARE
YP_001950543 Restriction endon  M----------------NHGHLSQYFSAVA----------CKPLSAVE---ADLCRSHQHELNGVEALIKMFGRATEKH---------TFQASF--MYLCDHDDEPVVADGYVTWYDSRL
YP_001045254 type II restricti  M-RYGS---------------LSDHFTGIV----------AKRLSTVE---ADTARSNQHEFNGTNELRRLLG--GERI--------ERRPSRF--IWLGG-ENEGITDDAPVTWYDARE
Q64Q06_BACFR Type II restricti  ---------------------MGEILNSAIVKIQNAKYAFCRFITAND---TGKNGSHQAGFYIPKCAAALLFDTPG-V------KGENKDKMV--KVKWQ-DDFTTDS--RFIYYGQ--
YP_001454985 hypothetical prot  M--------------------MSGLHSWLLDKSTSDTYMFIKRLSAND---TGATGGHQVGIYIPSGIVEHLFPSINHT------RDLNPSVML--NAHTS-SHNCPDSEARAIYYNGRF
EGE66164.1                      M---------------------SGLQDWLLDKVGGNNYLYIKRLSAND---TGATGGHQVGVYIPSGIVGELFPSIDHT------SEKNPSVLL--RATYS-SHECVDSEVRAIYYNNKY
ZP_08701146.1                   ------------------------------------------------------------------------------------------------------------------------
YP_001670968.1                  MAKMKHSTAFVPDLFTEPEAEASDFLTSLL---DKSDSLYVKKLSVNDRDWARLSNKRQAGIYVPST--------------------ERDGNFFPPLATKE-RDKAEAAEIRETYFEIEW
Q71SG8_KLEPN Restriction endon  M----------------------------------------QRLSPGE---------------------------------------------FKTLISKE-RKSHFIT-----------
EGT83115.1                      MNLTDV----------------------------------LIKIPDNE------------------------------------------IETFSAFIKQK-RDELISS-----------
AACY020325231                   M-----------------------------------------------------------------------------------------------------------------------

Mariner-1_OLpv-2p               ------------------------------------------------------------------------------------------------------------------------
Q8G4B6_BIFLO Type II restricti  ------------NNPNRSAEWRLYYKD---CEPIRMARPGDLMCFGMLRDNRLLIIIAQHDSTAEAQAKWLFGIDDEQEVAFRF-----HDNTERELDAFGAQIFEALGINVEVRDDT-Y
Q0AK57_MARMM Type II restricti  ------------NQPHRGPEWRLYYQA---NDITRAMKPGDQLFIVVLPDRTIGFLACDRDSSFQGLVEAIFRVQHSKSEQMVIIPV--SPDTSSPADYFTFQLLDALGIEPDIPPDF-D
Q727L4_DESVH Type II restricti  ------------RHATR-TEYRLYFPP---CEVMTEAREGDMIVFGLREDGSMLVIIGAAGTTGESQLSWLFGLSPAADGGYAVASP---ELLEQDIGFAGSYILEELGVEIAAAGDDGQ
YP_001950543 Restriction endon  ------------KNTTR-TEHRLYFPT---TAVSMCAAEGDLLIIGKRTDNSVMIIIAEAESTIASQVSWLFGFGVDVHPGFSVRSE--LECEQNRIEFASRIILEHIGVQVEEVAEN-Y
YP_001045254 type II restricti  ------------RHPTR-SEWRLYFQA---NAVTEVAQAGDLLVVARRPSGDLMFIVAPQSSTLENQIAWLFGLDHGLGAGFRYEGF--EGAGDRGLDFVSNYVLEEIGIEPEVPEAD-R
Q64Q06_BACFR Type II restricti  --------------GTR-NEYRITRFGKG-FPFFEEDNVGDLLIIAQQSEDYYYGFVLQTDQDIDDFFAY-FNLSPEKTNQLIDVTQ--ASTPEKQLEIGIQELVALYA-----------
YP_001454985 hypothetical prot  ------------FGKTR-NEKRITRWGGGKNPLQDPENTGALALLAFDHH---------QGSDSQFVDIW-VCHNAEEEDIVESSLG--EIVPGSLVYGPANEILGGLALKEPVSSG-YR
EGE66164.1                      ------------FGQTR-NEKRITRWGAG-SPLQDPENTGALSVLAFEHE---------PGRDSKSVDVW-VCVSPEEEDVLESAVG--EIIPGTLMFGPASEILGVLASRPKISQKEYV
ZP_08701146.1                   ------------------------------------------------------------------------------------------------------------------------
YP_001670968.1                  PTVGQVKRARLVHYTSKGEETHLTGLP---KPPFRDAPPASLLVVGK-SGGRYKALVVDSESDEYEVVHDVLSLPPDFHSEIFITTVVRREREEKTVSFIEEVVRAFF---------DGT
Q71SG8_KLEPN Restriction endon  ------------------------------------------------------------------------------------------------------------------------
EGT83115.1                      ------------------------------------------------------------------------------------------------------------------------
AACY020325231                   ------------------------------------------------------------------------------------------------------------------------

Mariner-1_OLpv-2p               ---------------------------------------------TTEI---KFNNQYVLSRMEENKLIKI----KLIIQELKQIAPL--------------------------------
Q8G4B6_BIFLO Type II restricti  LPEMIGRWGYRFPSNEEFAAFS------QSSLTDVDPTHD-DP--DDVVIE-YYDRSYLLFKLYE---RAV------IQHDYDAAPFVS----------------------DG-------
Q0AK57_MARMM Type II restricti  AASVVAQFKGTFPPTATFSTLA------RETLPGLDP-RD-DP--DKVLLA-WIDREEAMFRALE---RGI------VGDRLMLGFLDS----------------------RG-------
Q727L4_DESVH Type II restricti  LEHLLSTFGSAFPPTAVFSTYA------RDSLPHVSS-LD-DP--DGALVA-WFEQEERLFRMFE---RHL------VAQRLREGF----------------------------------
YP_001950543 Restriction endon  LDEMLRLFGGKFPNTRDFSEYA------RASLIDIDP-RD-GA--DAALMA-FVEREEILFRTLE---RHI------IGDRLQVGF----------------------------------
YP_001045254 type II restricti  LDEIVGRFGAQFPSSRTFSALA------RQNLPEVDP-RD-DA--DAALLA-WIEFEEALFRRLE---RHI------VAARLEVGFLT-----------------------DG-------
Q64Q06_BACFR Type II restricti  ----------DFPETRQMAQFARDIY-NKVHHITDVHICK-SP--DEQLLK-WIDTEYGLFRSFE---EKV------YAPIYSVPF----------------------------------
YP_001454985 hypothetical prot  LPE---EWRTNFPSGKEIIQYA------AAHYSPNV---I-GP--DKQLIE-RRRVEYEIFLLVE---EMH------VLGIVQSGF----------------------------------
EGE66164.1                      IPE---SWKHSFPSGLDIIQYA------AGYYPTGV---T-SP--DDQLLE-RRRIEYDLFLMVE---EIH------VLDIIRQGF----------------------------------
ZP_08701146.1                   MPT---DWLAKFPSGLEIVEKA------VALRPDNG---L-SP--DNRMLR-RRDCEFEIFRSVE---QAI------ELPNITAGF----------------------------------
YP_001670968.1                  FPELASRYG-AMPTTSELAAAARNRYLGENRLRSLDPYAMCKP--GDALRAISRGLEYEIFKDYQIKARSI------DLARMILGDDPS----------------------KASLERVLR
Q71SG8_KLEPN Restriction endon  ------------PFALVYKTFCDL----GYDQKNSDYFLN-NP--SEYIIA-MRKNCWKEFEPFEKEFTTRMLSYLIDEERIK----------------------------DMSPYDAIR
EGT83115.1                      ------------PKDTAKKA---T----EVNQKEISTLLN-KKQFSKAIIL-LREYSYQEFLKEEAAFNGV------ILKELSKKFKTPHNLIEKLSNHFFQLNIQEKTKFQEQLINTLG
AACY020325231                   ---------------------------------------------NKAL--------------EEWKLEQVNERNELIIKELRSRLPSCN------------------------------

Mariner-1_OLpv-2p               --LSDVELLQLYNKSISIHQGKIQGNGNFLENDILVGMLELNNI----PYKKQVTINKSGIIVGFNEKKGKCYHIIDFVIGENIEV---GKSITEYKVISCKTTCRERWTQDDWSYTF--
Q8G4B6_BIFLO Type II restricti  --VIDVDSF-T-SFYTSVRNRRMSRAGKVLEIHI-AHILDARGI----EYEAQAKTEN-----------G---KKPDFLFPSQAAYEDPAFPEEQLRMLASKTSIKDRFRQVADEANR--
Q0AK57_MARMM Type II restricti  --EPDVDAF-L-KFSLSVQNRRKSRAGHALMNHF-SKILRAFEI----PFTPEATTEK-----------K---GAADFLFPSEAAYADPGFPSANLRMVAAKTSCKDRWRQVLAEADR--
Q727L4_DESVH Type II restricti  --GEDVDAF-T-GYALSVLNRRKARAGKALENHV-EQVLKDWGV----PYTRECRTEN-----------N---SKPDFIFPGCSEYHQTNFPQDQLRMLAVKSTCKDRWRQVLAEAAR--
YP_001950543 Restriction endon  --VDDVDGF-V-TYSLSVQNRRKSRAGSSLENHL-EVIFNAQGI----QYTRTPVTEN-----------N---EKPDFIFPDIIHYRDPDFDVSKLTMLGVKSTCKDRWRQILSEADR--
YP_001045254 type II restricti  --TADVDGF-L-QFSLSVQNRRKSRMGLSLENHV-EEMLTVLGL----RYARGARTEG-----------N---SKPDFLFPGVAEYADPGYSADRLSMLGVKSTLKDRWRQVLAEAAR--
Q64Q06_BACFR Type II restricti  ---PNCQEL-V-KFSNIILNRRKSRAGKSLEHHL-ATIFTAAQL----EYEEQAVTED-----------N---KKPDFLFPNGEAYHNMLFPANKLVFLGAKTTCKDRWRQVLNEADR--
YP_001454985 hypothetical prot  ---GSVNEF-I-ALANSVSNRRKSRAGKSLELHL-EQLFNEHGL---KTFETQAVTEG-----------N---KKPDFLFPSAQAYHDEAFPEQKLRMLAVKTTCKDRWRQILNEADR--
EGE66164.1                      ---GSVDDF-I-AMANSVSNRRKSRAGKSLELHL-EQIFIEHGL---HHFATQCITEG-----------K---KKPDFLFPSAAAYHDDHFPREKLRMLAVKTTCKDRWRQALNEANK--
ZP_08701146.1                   ---SSVDEF-I-VRAQTILQRRKARSGRSLELHA-RAIFIEEQLVENTHFSHQPVSEP-----------G---KSPDFLFPSQEAYQDNSFPADQLRMLAVKTTCRDRWRQILNEADR--
YP_001670968.1                  AVVLDFPKI-D-ALLLSAAQQRKSRAGYSFEHHI-DAMLADAGV----PFEKQVVLEA-----------R---KRPDFILPSKAMYESSTRQHCEALVLSAKTTLRERWKQVKGEM-R--
Q71SG8_KLEPN Restriction endon  DFTMEYPTH-IYDLALSNTQSRRSRAGKEFESIL-ELLMMGAGI----PVDVQGAIGKSFFQ---KNQIG---KLVDLVMPGVVQYTSN---KRNTMLISAKTTLRERWQEVPEEVNRTG
EGT83115.1                      EYTGSISPY-IYQLCLSNTQSRRSRAGKIFEGII-YYLYEYLAF----SFDSQAQVGKKTFT---DLGLG---KLVDSVLPGIAEFNSR---RDKTIIGTMKTTLRERWQEVVEEVSRSN
AACY020325231                   --LLDQEHLEHFNTSVSIFQSMKCKIGKSFETAI-ENQLRLNGV----PFVSQVAIDKCGKIVGTGTTIKNHAHTLDILVGADSIEHIKGEHISNYVVISCKTSVRERWNQDEWTFEH--

Mariner-1_OLpv-2p               IPKLYILLTISDDYPPSTRFREDIQRKIITCIPKRKD--------DRIYKLKFEN-LIEQ--------------------------------------------------L
Q8G4B6_BIFLO Type II restricti  IRDKH-LFTLTPGDVTHPKLAQLDELHIHLVMPKVVKESYDDL--IQGETMTFSR-FIEE----IQGLQAD--------------------------------RPQSLTLL
Q0AK57_MARMM Type II restricti  IKPKH-LLTLQPK-ISLAQTTEMTDQGIILTLPSPIHSSFQDD--QRASLLTVAD-LLQE----LASL-------------------------------------------
Q727L4_DESVH Type II restricti  VSRKH-LLTLEPG-ISVQQTTEMVDHAVQLVIPRGIQSSFQDA--QREELISLGE-FVGM----AQANLWS---------------------------------------S
YP_001950543 Restriction endon  IKSKH-LLTLEPG-ISENQTNQMKRSNLQLVVPSALQRTYKTE--QQTWLLTFDD-FIRL----LRIRQS-----------------------------------------
YP_001045254 type II restricti  IDRKH-LLTLEPG-ISTHQTNEMIRHSLQLVVPRGLHTTYTPE--QAGWLMTVRG-FLNL----VAAREAA--------------------------------RPYR---E
Q64Q06_BACFR Type II restricti  IETKY-LFTLQQG-ISKNQLREMKHEHLKLVVPASYRTSFDKE--FQPEIETLAS-FIEM----VKLKQSS--------------------------------KPIYS--V
YP_001454985 hypothetical prot  IQDIY-LFTLQEG-VSVAQFQEMQQERVRLVVPSSLHDKYPKA--IREYLISLET-FIDE----TK--------------------------------------SLYVD--
EGE66164.1                      INKIY-LFTLQEG-VSVAQFNEMRAEGVTLVVPSPLHKKYPEE--IREEILTLDG-FITQ----LI--------------------------------------TLYEDFV
ZP_08701146.1                   IPRKH-LLTLQEG-VSENQFREMTSAGVTLVVPEKLREKFPKP--VRPHLQNLES-FIGD----VR--------------------------------------SLAAR-T
YP_001670968.1                  DCDLY-LATVDES-IAVNAIKDMASQGIRLVVPESLKSSDTTEYKAQDSVLSFKD-FFENDIAARRKLWIA--------------------------------NGIFATGV
Q71SG8_KLEPN Restriction endon  IREMY-LATLDDS-FSEETINILYEANV-VVVTTIENKNFKYK--NNNRVLTFED-MLQSAMELSRKWNNVSYTDSEKEEIQRSILKQIEKYSDFPYVVNYYRNRLSALFD
EGT83115.1                      IPNIY-LLTVDND-ISDNKAVQMGTHNIVLVVLNEVKNQKHLK--DKRSVIDFESYFLDEIPNIMKYWKK-----------------------------------------
AACY020325231                   KPKLYMLCTLSRDYPNSEKFQEGPTRKIATDDPKQKD--------DRKYKLDLED-LINE---------------------------------VVVNYVVENVLIKSLSAV
```
