# Supplementary material for: Homologues of bacterial TnpB_IS605 are widespread in diverse eukaryotic transposable elements
Source: Mob DNA. 2013 Apr 1;4:12. doi: 10.1186/1759-8753-4-12 (PMC3627910; doi:10.1186/1759-8753-4-12)
Supplement: Additional file 8 — Alignment of the 5′-ends of CRe-1, 2, 3 and those of other Helitrons (A) and the sequences of 15 CRe-1, 2, 3 insertions (B) [file 1759-8753-4-12-S8.pdf]

Additional file 8A

```
                           10        20        30        40        50        60        70        80        90
                   ....|....|....|....|....|....|....|....|....|....|....|....|....|....|....|....|....|....|
CRe-1              TGGGCTGAGCCCCCCCAGCGAAGCGTTCCAGGGGGGGGGCGAAGCCCCCCAGGAACACCCCTGTCCGTGGC------TGGTGGCTGG---
Helitron-1N1_CRe   TGGGCTGAGCCCCCCCAGCGAAGCGTTCCAGGGGGGGGGCGAAGCCCCCCAGGAACACCCCTGTCCGTGGC------TGCGTGC--G---
CRe-2              TGGGCTGAGCCCCCTGAGCGAAGCGATCCAGGGGGGG--CGAAGCCCCCCAGGATTGCCCCTGTCCGTGCC------TGCCTGCCTGCCT
Helitron-1N2_CRe   TGGGCTGAGCCCCCCGAGCGAAGCGATCCAGGGGGGGG-CGAAGCCCCCCAGGATTGCCCCTGTCCGTGCC------TGCCTGCCTGCCT
CRe-3              TGGGCTGAGCCCCCCGAGCGAAGCGATCCAGGGGGGGG-CGAAGCCCCCCAGGATTGCCCCTGTCCGTGCCTGCCGGTGCCTGCCTGCCT
Helitron-1_CR      TGGGCTGAGCCCCCCGAGCGAAGCGATCCAGGGGGGGG-CGAAGCCCCCCAGGATTGCCCCTGTCCGTGCCTGCC--TGCCTGCCTGCCT

                          100       110       120       130       140       150       160       170       180
                   ....|....|....|....|....|....|....|....|....|....|....|....|....|....|....|....|....|....|
CRe-1              -------------------TGCGTCGGCAAAACATAGCATACTGGCGCAAACCGCGGCTGCTACGTATTAGTATGTGTTAATGCCTATTG
Helitron-1N1_CRe   -------------------TGCGTCGGCAAAACATAGCATACTGGCGCAAACCGCGACTGCTACGTATTAGTATTTGTTAATGCCTATTG
CRe-2              GCCTGCC----TGCCTGCCTGCGTTGACAAAAAGTGCCAAACTGGTGCAAACCGCGAGCGCCACGTATTATAAATTGCTATTCCCTATTG
Helitron-1N2_CRe   GCGTGCC--------TGCCTGCGTCGACAAAAAGTGCCAAACTGGCGCAAACCGCGAGTGCCACGTATTATTAATTGCTATTCCCTATTG
CRe-3              GCCTGCCGG--TGCCTGCCTGCGTCGACAAAAAGTGCCAAACTGGCACAAACCGCGAGCGCCACGTAATATTACTTGCTATTCCCTATTG
Helitron-1_CR      GCCTGCCTGCGTGCCTGCCTGCGTCGACAAAAAGTGCCAAACTGGCACAAACCGCGAGCGCCACGTATTATAAATTGCTATTCCCTATTG

                          190       200       210       220       230       240       250       260       270
                   ....|....|....|....|....|....|....|....|....|....|....|....|....|....|....|....|....|....|
CRe-1              CATGTAAATAGGTGGCAGGGAAAATTCGGGCGGAGCGAGAAGTGCCTTCGCACGTCCATGGACAAT---TGACTTTCG-CGAGGTTGCCC
Helitron-1N1_CRe   CATGTAAATAGGTGGCAGGGAAAATTCGGGCGGAGCGAGAAGTGCCTTCGCGCGTCCATGGACAAT---TGACTTTCG-CGAGGTCGCGA
CRe-2              TAGAAAAATAGGCGGCAGGGAAAACTCGGCTGGAGCGAGAAGCGACCTCGCGAGTCCATGGACATC--TTGACTTTCG-CGAGGTCGCGA
Helitron-1N2_CRe   TAGAAAAATAGGCGGCAGGGAAAACTCGGCCGGAGCGAGAAGCGACCTCGTGAGTCCATGGACATC--TTGACTTTCTTC-----AGT--
CRe-3              TAGAAAAATAGGCAGCAGGTAAAACTCGGCCGGAGCGAGAAGCGACCTCGCGCGTCCATGGACTTTAATTGACTGGCGACTGTGGCGACT
Helitron-1_CR      TATAAAAATAGGCGGCAGGGCAAATTCGGCCGGAGCGAGAAGCGACCTCGCGAGTCCATGGACATC--TTGACTTTCGTCTTGCGAGCGA

                          280       290       300       310       320
                   ....|....|....|....|....|....|....|....|....|....|....
CRe-1              GAAAAGCAAGC--CATGGCTTTGCGCAGTATGACTTCTGTACGTCG--------
Helitron-1N1_CRe   GGAAAGCATCC--GGTGCCCGC---TGATATGCAGTCTCTAAGGCA--------
CRe-2              GGT-CGCAAGGAAAGCAGCCCT--GTCACGCGGATTCCACAAAGGA--------
Helitron-1N2_CRe   --C-CGCGAG---TATAGCTCTC-GGCCCCTAAATATCTTACATCCATGTATCA
CRe-3              CGT-TGCGAGGTAAAGGGCCC---GAGGCGCAGAATT---AAGAGG--------
Helitron-1_CR      GAG-TGCAGGCTTGATAGTTTCT-TCCGCACAAAACTATACCGACCGCATCCAA
```

Additional File 8B

The following sequences represent the 15 loci shown in Figure 4A. TSDs are colored in pink.

> Chlamydomonas reinhardtii Locus-1, ABCN01002739.1 6698-2666, gray region correspond to 1-3992 of CRe-1

TTTTTCTGGTCTGAGCCAGCGCAGGAGCAGCCCTCCAGCCCCCAACACGCCAATCC
ACATTGTTCTCTGCTATCGAAATGGTTATTTTTGGGCTGAGCCCCCCCAGCGAAGCGTTCCAGGGGGTGG
GCGAAGCCCCCCAGGAACACCCCTGTCCGTGGCTGGTGGCTGGTGCGTCGGCAAAACATAGCATACTGGC
GCAAACCGCGGCTGCTACGTATTAGTATGTGTTAATGCCTATTGCATGTAAATAGGTGGCAGGGAAAATT
CGGGCGGAGCGAGAAGTGCCTTCGCACGTCCATGGACAATTGACTTTCGCGAGGTTGCCCGAAAAGCAAG
CCATGGCTTTGCGCAGTATGACTTCTGTACGTCGTAAATGTGCTACAAAGACACAGCAGACGGCGAGCCG
TCATAAAGAGCCTGTCACCGTTGTCCATTTCTGTAGCGGCTCGCTCTCACGCCTCTTTCTTCCTGGTCCG
AGCGCAACCCCCAGTGGACGCGTCCGGACCAGCCATGGCACCGAAGCGGCGCCGAGACGAAGCTGAGAAG
GCGGAGGATGAGAAAGGTGGGGGAGCGATGACCCGCCAGCGCCCGTCCGCTCATCACAGCCCCTCCGCAG
CCGCTCGCAATCTGCATGCTGCCAAGCTGTCGCAAGACTGGTAGAGGGGCGCACAACGTGTCGAGGCTTT
GCGTTGCTTGGCATCTGGTCACGCATCTGCTTTCCTTTACTATTCCCCGCAGACCACACAACGTCGACCA
AATGCGGTCTAGCTGGACTTCTGTCTGAGAAGATAGAAGCCGATGGCGTGGCGGTCACGCGCGAGGAAAG
CCTCGCTGCCGTTGACTTCTTGGTCGCGGCCCTCACCAGGCTGCGCTTTGAGGCGCTGTGCCTGCTAGGG
CTGGTAGCGGTCCGCATGTGCGAGGACGCACGGCGAGAAGGTCAAGGCCTGCAGCCGCACTGCGCAACGT
GTCGCCGGCTTCGGAAGACGGAGCTGGTGGAGGACGACACGACACGACACGCGGCGATCTGCGCTGTGTC
GGTGTGTGACCTCACCGAGCAGGGCCGGAAAAGGGGCCGGCCGTCCAAGCGGGACCAGCATCCTGAGGAC
GACCTGTTCCGGCACGTCTGCGAGGAGCACTTCCCGCGCGACGAGGAGGCCGCGGGTGCTCGCGTCAACA
GGTCGGGCTTGACTCCCTTCCTGCCGCCGTTGTCGAAGGGCGTGTTTACCAACGTCAAGAACCACTACGC
CGCAAACTTCGCAGCCTGGCTGGCGCGCAGCTTTCGCTGCCGCATCGACGACGAGCTTCGCGAGGTGGGG
CAGGCGGAGGTGGGGCAGGCGGAGGTGGGGCAGGCGGAGGTGGGGCAGGCGGAGGTGGGGCAGGCGGAGG
CCGAAGGAAGCGGCGCCTTTACTAACAAAAGTTTTTCTCCTTTCCCACGTTCCATGTCCCGCAGCTCCGT
ACTCCAGCCACCAAAAAGCTGGACAAGCTGGCATGGAGCATGGCGCATGCCGTGCTCTACGACGGCGAGT
TAGAGCAGCCGCGCTGGTGGGTGGGTTGGGCCCAGGGCGCCGCCGCGCAAGCCGCGGGCGCGGCGGCTGC
GGCGGCGGCACAGGGCGCCGGCCCTGCGGGCGGGGCCGCCGCCGCGCAAGCCGCGGGCGCGGCGGCTGCG
GCGGCGGCACATGGCGCCGGCCCTGCGGGCGGGGCCGCCGCCGCGCAAGCCTGGACCGCCCTCGTTGACT
ACGTCAACGCCCAGCGTGCGTCGAAAAGGGCCGCAGAGCTGTTGTTGCGGGAGGTCAAGGGGACGCAGGC
CACGTACAAGAAGGCCAGCACGCGGCACATGGAATGGGCCGCGGAGATTCTCGCAGGCCTTGAGGCACGC
CGCGATCAGCTGGGGGCGCAGGTGCAGCAACTGACGCAGGCGCAGCCGTTGACGCGAGAGGACACGCAGC
GGCTTGCGTCCTTGCGCCGGGAACTGCATCGCGCACGTCCGTTCACGCTGACCCCCAGCCCCTCCTTCGC
GCCGATCTATGTGCCCCTTGACAACACGTCCATGGCCCGCCTTCCTGGGCTGCTGCCCACCCTGGCAAGG
CGCCATGGCGAGGTCTTCGCCGGTGCAGGCGCGGGCGCCGTGGCACCCTCCTCGTTTGTCCAGGCCGCGT
TTGGCGGCGGGGGCATGCAGTCGTCTGCCACGCTGAACGCCGTCGGCTGGGGCCTGTTCCAGCTCGGGGG
TGTGACCTCCCGCAACGCCCCCTTCGCCAATTACATCACCACGGACGGGGTGGCCTGCAGCGTGGCGCGA
GAAGCGCACAACAAGCCGCTGGCCAACTTGAAGCCGGCGACCGCGCCCGCCGATGCGGGGGAGCTGTGCA
CCCTGGAGGAGATGAAGGCCACGCAGATCATCGGTGTGGACCCGTGCGGGGGAGGGAATTGGTTCATGGC
AGCACGGTCTCCGCTGTACCAGCCAGGGCCCTGGGCTTGGGAGGGTGTCGGGGAGGGTGTCGGCCCTGCC
CAGCGCTACCTGCTCGAGCTGCACGACAAGCAGCTGGATGAGGAGTTGTTCCCTGGGCAGCTGCCGCCGG
AGCCGCGTCGCAGGCGCAAGGGCGTCAGGCGCAAGGGGGGCGGGCGTAAGGGGGGCCACCGCCGGAAGCA
ATCTAAGCACTGGCAGCCGCGAGCCAGGACAGCGCGGCGGCGGCGCCAGAAGCGGGGCCGATTCCATATG
AGCATGGGCCACTGGCGGCACATGTCAGGCCTGGAACGACTCCAGCCAAACCGCCCACAGCTTGCGCCAG
CCCTGCAGGCGTACATCGGCGGCATCCCCACGGCGGCGACCGCGTCGGCGGCCCGGTTTGAAGAGCGCCT
GCGCTACCTCTTCGCCAGTGGGGCGGCAGGGCAGGCGGCGGGGCAAGCGGCGGGGCAGGCGGCGGGGCAA
GCGGCGGGGCAGGCGGCGGGGCAGGCGGCGGGGCAGGCAGAGGCGGGGCAGGCAGAGGCGGGGCAGGCAG
AGGCGGGGCCCCGGGGCGCGGTGCACGTGCTGTGGCACTACCACTTCTCCGCCTTCCGCCGAAAGCGCTG
GGCTGCGTTCATCCAGCGCGACCGCGCGCTCCACCGCGTCGCCAAGCAGCTCACGGGCGGCAGGCCCAAG
GAAGAGGTGGTGGTGGGGTGGGGTTCGTGGGCCTTCCAGGGAGGGAAGGGCGGCTCCCCCATCTCCGTCA
GGGGCGGGCGCGCGCCGACGGGGCGGCTCATCAAGCTGCTCCGTAAGCGCTACGCCAAGCATGTGTTCAT
CATCGATGAATACAAGACCTCCAAGGTGGGTGGGTTGAGTGGGATTGCAATTGCTGGTGGGTGGGTGGGC
CGCTGGGTTTATGCCTGGCTTTAGCGGAAGCCCTTGTGTGGCCCCCCTCCCTCCCACCCTCCCGACCATG
CAGACCTGCTACAACTGCGGGTGTCAGGAGATGGCCATCAAGCGGCTTGGGGGGCTGAAGGAGGGGCAGC
GGCCCTGGTCGGTCAAAGTCTGCAACGACTGCTTGACGACCTGGGTGAGGACCGTGCATGTGCGGGGACG
GCTGGGCACTGGGGACGGATTGATTGACAGGTTGACGGCTGAGACAGCTCATGTGCGCTCTCTTCTTCCT
CCCCGTTCCCCATCCCCAGAACCGCGACGTCTCCGCCGCCAACGTGATCCGTGTGCTCCTCCTGCTGAAG
CTGATGGGCTTCGAGCGGCCGACCAAGCTGCAGCGGCCGCCATGGCCGCCGGCGGCGGCGGGGCCGGGCT
GAGAGCCTGAACGGCGCTAGCAGGGCGTGGGGCTGAGGGTGCACGTGTTGATTGGCGGCGAGTGACGTGA
CTAGTTTGTTAGCTGCGGGTTAGCACGGACTGTGCACCCCACCCCACCGGCCACGTTCCGGATTTGCGGG

GATGCAAAGGCCCCCAACATAGAGGCGTGTGCTTAGTAGGCGCCCGCGTCAAGGTGGCTGGGTTGATAAT
GACCCGGGATCAGCCCTTGTCCTGCCATAAGGCAGCCACCTTCTTGTTCTGGTCTGA

> CRe_Locus-2, Chr_2 9231256-9228981, correspond to 1-138; 1941-3992 of CRe-1

TTTTTGGGCTGAGCCCCCCCCAGCGAAGCGTTCCAGGGGGGG
GCAAAGCCCCCCAGGAACACCCCTGTCCGTGCGTGCGTGCGTGGCTGCGTGCGTCGGCAAAACATAGCAT
ACTGGCGCAAACCGCGACTGCTACGTATAAGGAACTGCATCGCGCACGTCCGTTCACGCTGACCCCCAGC
CCCTCCTTCGCGCCGATCTATGTGCCCCTTGACAACACGTCCATGGCCCGCCTTCCTGGGCTGCTGCCCA
CCCTGGCAAGGCGCCATGGCGAGGTCTTCGCCGGTGCAGGCGCGGGCGCCGTGGCACCCTCCTCGTTTGT
CCAGGCCGCGTTTGGCGGCGGGGGCATGCAGTCGTCTGCCACGCTGAACGCCGTCGGCTGGGGCCTGTTC
CAGCTCGGGGGTGTGACCTCCCGCAACGCCCCCTTCGCCAATTACATCACCACGGACGGGGTGGCCTGCA
GCGTGGCGCGAGAAGCGCACAACAAGCCGCTGGCCAACTTGAAGCCGGCGACCGCGCCCGCCGATGCGGA
GGAGCTGTGCACCCTGGAGGAGATGAAGGCCACGCAGATCATCGGTGTGGACCCGTGCGGGGGAGGGAAT
TGGTTCATGGCAGCACGGTCTCCGCTGTACCAGCCAGGGCCCTGGGCTTGGGAGGGTGTCGGGGAGGGTG
TCAGCCCTGCCCAGCGCTACCTGCTCGAGCTGCACGACAAGCAGCTGGATGAGGAGTTGTTCCCTGGGCA
GCTGCCGCCGAAGCCGCGTCGCAGGCGCAAGGGCGTCAGGCGCAAGGGTGGCGGGCGCAAGGGGGGCCAC
CGCCGGAAGCAATCGAAGCACTGGCAGCCGCGAGCCAGGACAGCGCGGCGGCGGCGCCAGAAGCGGGGCC
GATTCCTTATGAGCATGGGCCACTGGCGGCACATGTCAGGCCTGGAACGACTCCAGCCAAACCGCCCACA
GCTTGCGCCAGCCCTGCAGGCGTACGTCGGCGGCATCCCCACGGCGGCGACCGCGTCGGCGGCCCGGTTT
GAAGAGCGCCTGCGCTACCTCTTCGCCGGTGGGGCGGCAGGGCAGGCGGCGGGGCAGGCGGCGGGGCAGG
CGGCGGGGCAGGCGGCGGGGCAGGCGGCGGGGCAGGCGGCGGGGCAGGCGGCGGGGCAGGCGGCGGGGCA
GGCGGCGGGGCAGGCGGCGGGGCAGGCGGCGGGGCAGGCGGCGGGGCAGGCGGCGGGGCAGGCGGAGGCG
GCGGGCGGGGCCGGCAGAGGCGGGGCCCCGGGGCGCGGTGCACGTGCTGTGGCACTACCACTTCTCCGCC
TTCCGCCGAAAGCGCTGGGCTGCGTTCATCCAGCGCGACCGCGCGCTCCACCGCGTCGCCAAGCAGCTCA
CAGGCGGCAGGCCCAAGGAGGAGGTGGTGGTGGGGTGGGGTTCGTGGGCCTTCCAGGGAGGGAAGGGCGG
CTCCCCCATCTCCGTCAGGGGCGGGCGCGCGCCGACGGGGCGGCTCATCAAGCTGCTCCGTGAGCGCTAC
GCCAAGCATGTGTTCATCATCGATGAATACAAGACCTCCAAGGTGGGTGGGTTGAGTGGGATTGCAATTG
CCGGTGGGTGGCTTGGCCGCTGGGTTTATGCCTGGCTTTAGCGGAAGCCCTTGCGTGGCCCCCCTCCCTC
CCACCCTCCCGACCATGCAGACCTGCTACAACTGCGGGTGTCAGGAGATGGCCATCAAGCGGCTTGGGGG
GCTGAAGGAGGGGCAGCGGCCCTGGTCAGTCAAAGTCTGCAACGACTGCTTGACGACCTGGGTGAGGACC
GTGCATGTGCGGGGACGGCTGGGCACTGGGGACGGATTGATTGACAGGTTGACGGTTGGGACAGCTCATG
TGCGCTCTGCTCTTCCTCCCCGTTCCCCATCCCCAGAACCGCGACGTCTCCGCCGCCAACGTGATCCGTG
TGCTCCTCCTGCTGAAGCTGATGGGCTTCGAGCGGCCGACCAAGCTGCAGCGGCCGCCATGGCCGCCGGC
GGCGGCGGGGCCAGGCTGAGAGCCTGAACGGCGCTAGCAGGGGGTGGGGCTGAGGGTGCACGTGTTGATT
GGCGGCGAGTGACGTGACTAGTTTGTTAGCTGCGGGTTAGCACGGACTGTGCACCCCACCCAACCGGCCA
CGTCCGGATTTGCGGGGATGCCAAAGGCCCCCAACATAGAGGCGTGTGCTTAGTAGGCGCCCGCGTCAAG
GTGGCTGGGTTGATCACGACCCGGGATCAGCCCTTTTCCTGCCATAAGGCAGCCACCTCCTTGT

> CRe_Locus-3, Chr_2 4215026-4216380, correspond to 1-164, 2742-3992 of CRe-1
TTTTTGGGCTGAGCCCCCCCAGCGCAGCGTTCCAGGGGGGGGCCGAAGCCCCCCAGGAACCCCCC
TGTCCGTGCTGCGTTCGTGCGTCGGCAAAACATAGCATACTGGCACAAATCGCGACTGCCACGTATTAGT
ATTTGTTAATGCCTATTGCATGTAAATAGGCCGCCCACACCTTGCGCCAGCCCTGCAGGCGTACGTCGGC
GGCATCCCCACGGCGGCGACCGCGTCGGCGGCCCGGTTTGAAGAGCGCCTGCGCTACCTCTTCGCCAGTG
GGGCGGCAGGGCAGGCGGCGGGGCAGGAGGCGGGGCAGGCGGAGGCGGCGGGGCAGGCGGAGGCGGCGGG
GCCGGCAGAGGCGGGGCCCCGGGGCGCGGTGCACGTGCTGTGGCACTACCACTTCTCCGCCTTCCGCCGA
AAGCGCTGGGCTGCGTTCATCCAGCGCGACCGCGCGCTCCACCGCGTCGCCAAGCAGCTCACGGGCGGCA
GGCCCAAGGAGGAGGTGGTGGTGGGGTGGGGTTCGTGGGCCTTCCAGGGAGGGAAGGGCGGCTCCCCCAT
CTCCGTCAGGGGCGGGCGCGCCGCCAAGCATGTGTTCATCATCGATGAATACAAGACCTCCAAGGTGGGT
GGGTTGAGTGGGATTGCAATTGCTGGTGGATGGGTGGGCCGCTGGGTTTATGCCTGGCTTTAGCGGAAGC
CCTTGTGTGGCCCCCCGATCCCTCCTACCCTCCCGACCATGCAGACCTGCTACAACTGCGGGTGTCATGA
GATGGCCATCAAGCGGCTTGGGGGGCTGAAGGAGGGGCAGCGGCCCGGGTCGGTCAAAGTCTGCAACGTC
TGCTTGACGACCTGGGTGAGGACCGTGCATGTGCGGGGACGGCTGGGCACTGGGGACGGATTGATTGACA
GGTTGACGGTTGGGACAGCTCATGTGCGCTCTCTTCTTCCTCCCCGTTCCCCGTCCCCAGAACCGCGACG
TCTCCGCCGCCAACGTGATCCGTGTGCTCCTCCTGCTGAAGCTGATGGGCTTCGAGCAGCCGACCAAGCT
GCAGCGGCCGCCATGGCCGCCGGCGGCGGCGGGGCCGGGCTGAGAGCCTGAACGGCGCTAGCAGGGCGTG
GGGCTGAGGGTGCACGTGTCGATTGGCGGCGAGTGACGTGACTAGTTTGTTAGCTGCGGGTTAGCACGGA
CTGTGCACCCCACCCAACCGGCCACGTCCGGATTTGCGGGGATGCCAAAGGCCCCCAACATAGAGGCGTG
TGCTTAGTAGGCGCCCGCGTCAAGGTGGCTGGGTTGATAACGACCCGGGAGGGGAGGGCTCAGCCCTTTT
CCTGCCTCCCTAAGGCAGCCACCTCCTTGT

> CRe_Locus-4, Chr_6 1673311-1672251, correspond to 6-328, 3270-3992 of CRe-1
TTTTTGGTGAAGCTGAGCCCCCCCAGCGAAG

CGTTCCAGGGGGGGGCGAAGCCCCCCAGGAACACCCCTGTCCGTGGCTGCGTGGCTGCGTGGCTGGTGCG
TCGGCAAAACATAGCATACTGGCACAAACTGCGACTGCTACGTATTAGTATTTGTTAATGCCTATTGCAT
GTAAATAGGTGGCAGGGAAAATTCGGGCGGAGCGAGAAGTGCCTTCGCACGTCCATGGACAATTGACTTT
CGCGAGGTTGCCCGAAAAGCAAGCCATGGCTTTGCGCAGTATGACTTCTGTACGTCGTAAATGTGCTTCA
AAGACACAGCAGACGGCGAGCCGTCATAAAGAGTGGGATTGCAATTGCTGGTGGGTGGGTGGGCCGCTGG
GTTTATGCCTGGCTTTAGCGGAAGCCCTATTGTGGCCCCCCTCCCTCCTACCCTCCCGACCATGCAGACC
TGCTACAACTGCGGGTGTCAGGAGATGGCCATCAAGCGGCTTGGGGGGCTGAAGGAGGGGCAGCGGCCCT
GGTCGGTCAAAGTCTGCAACGACTGCTTGACGACCTGGGTGAGGACCGTGCATGTGCGGGGACGGCTGGG
CACTGGGGACGGATTGATTGACAGGTTGACGGCTGAGACAGCTCATGTGCGCTCTCCTCTTCCTCCCCGT
TCCCCATCCCCAGAACCGCGACGTCTCCGCCGCCAACGTGATCCGTGTGCTCCTCCTGCTGAAGCTGATG
GGCTTCGAGCGGCCGACCAAGCTGCAGCGGCCGCCATGGCCGCCGGCGGCGGCGGGGCCGGGCTGAGAGC
CTGAACGGCGCTAGCAGGGCGTGGGGCTGAGGGTGCACGTGTCGATTGGCGGCGAGTGACGTGACTAGTT
TGTTAGCTGCGGGTTAGCACGGACTGTGCACCCCACCCAACCGGCCATGTCCGGATTTGCGGGGATGCCA
AAGGCCCCCAACATAGAGGCGTGTGCTTAGTAGGCGCCCGCGTCAAGGTGGCGGGGTTGATAACGACCCG
GGATCAGCCCTTTTCCTGCCATAAGGCAGCCACCTCCTTGTTGGTGAAGC

> CRe_Locus-5, Chr_14 772703-776621, correspond to 132-3992 of CRe-1
TTTTTAGTGCATCAAAGTAATGTGTTAATGCCTATTGCATGTAAATAGGTGGCAGGGAA
AATTCGGGCGGAGCGAGAAGTGCCTTCGCACGTCCATGGACAATTGACTTTCGCGAGGTTGCCCGAAAAG
CAAGCCATGGCTTTGCGCAGTATGACTTCTGTACGTCGTTAATGTGCTTCAAAGACACAGCAGACGGCGA
GCCGTCATAAAGAGCCTGTCACCGTTGTCCATTTCTGTAGCGGCTCGCTCTCACGCCTCTTTCTTCCTGG
TCCGGGCGCAACCCCCAGTGGACGCGTTCGGACCAGCCATGGCACCGAAGCGGCGCCGAGACGAAGCTGA
GAAGGCGGAGGAGGAGAAAGGTGGGGGAGCGATGACCCGCCAGCGCCCGTCCGCTCATCACAGCCCCTCC
GCAGCCGCTCGCAATCTGCATGCTGCCAAGCTGTCGCAAGACTGGTAGAGGGGCGCACAACGTGTCGAGG
CTTTGCGTTGCTTGGCATCTGGTCACGCATCTGCTTTCCTTTACTATTCCCCGCAGACCACACAACGTCG
ACCAAATGCGGTCTAGCTGGACTTCTGCCTGAGAAGATAGAAGCCGATGGCGAGGCGGTCACGCGCGAGG
AAAGCCTCGCTGCCTTTGATTTCCTGGTCGCGGCCCTCACCAGGCTGCGCTTTGAGGCGCTGTGCCTGCT
AGGGCTGGTAGCGGTCCGCATGTGCGAGGACGCACGGCGAGAAGGTCAAGGCCTGCAGCCGCACTGCGCA
ACGTGTCGCCGGCTTCGGAAGACGGAGCTGGTGGAGGACGACATGTACGCGGCGATCTGCGCTGTGTCGG
TGTGTGACCTCACCGAGCAGGGCCGGAAAAGGGGCCGGCCGTCCAAGCGGGACCAGCATCCTGAGGACGA
CCTGTTCCGGCACGTCTGCGAGGAGCACTTCCCGCGCGACGAGGAGGCCGCGGGTGCTCGCGTCAACAGG
TCGGGCTTGACTCCCTTCCTGCCGCCGTTGTCGAAGGGCGTGTTTACCAACGTCAAGAACCACTACGCCG
CAAACTTCGCAGCCTGGCTGGCGCGCAGCTTTCGCTGCCGCATCGACGACGAGCTTCGCGAGGTGGGGCA
GGCGGAGGTGGGGCAGGCGGCGGTGGGGCAGGCGGAGGTGGGGCAGGCGGAGGCCGAAGGAAGCGGCGCC
TTTACTAACAAAAGTTTTTCTCCTTTCCCACGTTCCATGTCCCGCAGCTCCGTACTCCAGCCACCAAAAA
GCTGGACAAGCTGGCATGGAGCATGGCGCATGCCGTGCTCTACGACGGCGAGTTAGAGCAGCCGCGCTGG
TGGGTGGGTTGGGCCCAGGGCGCCGCCGCGCAAGCCGCGGGCGCGGCGGCTGCAGCGGCGGCACATGGCG
CCGGCCCTGCGGGCGGGGCCGCCGCCGCGCAAGCCGCGGGCGCGGCGGCTGCGGCGGCGGCACATGGCGC
CGGCCCTGCGGGCGGGGCCGCCGCCGCGCAAGCCGCGGGCGCGGCGGCTGCGGCGGCGGCACATGGCGCC
GGCCCTGCGGGCGGGGCCGCCGCCGCGCAAGCCTGGACCGCCCTCGTTGACTACGTCAACGCCCAGCGTG
CGTCGAAAAGGGCCGCAGAGCTGTTGCTGCGGGAGGTCAAGGGGACGCAGGCCACGTACAAGAAGGCCAG
CACGCGGCACATGGAATGGGCCGCGGAGATTCTCGCAGGCCTTGAGGCACGCCGCGATCAGCTGGGGGCG
CAGGTGCAGCAACTGACGCAGGCGCAGCCGTTGACGCGAGAGGACACGCAGCGGCTTGCATCCTTGCGCC
GGGAACTGCATCGCGCACGTCCGTTCACGCTGACCCCCAGCCCCTCCTTCGCGCCGATCTATGTGCCCCT
TGACAACACGTCCATGGCCCGCCTTCCTGGGCTGCTGCCCACCCTGGCAAGGCGCCATGGCGAGGTCTTC
GCCGGTGCAGGCGCGGGCGCCGTGGCACCCTCCTCGTTTGTCCAGGCCGCGTTTGGCGGCGGGGGCATGC
AGTCGTCTGCCACGCTGAACGCCGTCGGCTGGGGCCTGTTCCAGCTCGGGGGTGTGACCTCCCGCAACGC
CCCCTTCGCCAATTACATCACCACGGACGGGGTGGCCTGCAGCGTGGCGCGAGAAGCACACAACAAGCCG
CTGGCCAACTTGAAGCCGGCGACCGCGCCCGCCGATGCGGGGGAGCTGTGCACCCTGGAGGAGATGAAGG
CCACGCAGATCATCGGTGTGGACCCGTGCGGGGGACGGAATTGGTTCATGGCAGCACGGTCTCCGCTGTA
CCAGCCAGGGCCCTGGGCTTGGGAGGGTGTCGGCCCTGCCCAGCGCTACCTGCTCGAGCTGCACGACAAG
CAGCTGGATGAGGAGTTGTTCCCTGGGCAGCTGCCGCCGGAGCCGCGTCGCAGGCGCAAGGGTGGCGGGC
GCAAGGGGGGCGGGCGTACGGGGGGCCACCGCCGGAAGCAATCTAAGCACTGGCAGCCGCGAGCCAGGAC
AGCGCGGCGGCGGCGCCAGAAGCGGGGCCGATTCCATATGAGCATGGGCCACTGGCGGCACATGTCAGGC
CTGGAACGACTCCAGCCAAACCGCCCACAGCTTGCGCCAGCCCTGCAGGCGTACATCGGCGGCATCCCCA
CGGCGGCGACCGCGTCGGCGGCCCGGTTTGAAGAGCGCCTGCGCTACCTCTTCGCCAGTGGGGTGGCAGG
GCAGGCGGCGGGGCAGGCGGCGGGGCAAGCGGCGGGGCAGGCGGCGGGGCAGGCGGCGGGGCAAGCTGCG
GGGCAGGCGGCGGGGCAGGCGGCGGGGCAAGCGGCGGGGCAGGCGGCGGGGCAGGCGGCGGGGCAGGCGG
CGGGGCAGGCGGCGGGGCAGGCAGAGGCGGGGCCCCGGGGCGCGGTGCACGTGCTGTGGCACTACCACTT
CTCCGCCTTCCGCCGAAAGCGCTGGGCTGCGTTCATCCAGCGCGACCGCGCGCTCCACCGCGTCGCCAAG
CAGCTCACAGGCGGCAGGCCCAAGGAGGAGGTGGTGGTGGGGTGGGGTTCGTGGGCCTTCCAGGGAGGGA
AGGGCGGCTCCCCCATCTCCGTCAGGGGCGGGCGCGCGCCGACGGGGCGGCTCATCAAGCTGCTCCGTGA
GCGCTACGCCAAGCATGTGTTCATCATCGATGAATACAAGACCTCCAAGGTGGGTGGGTTGAGTGGGATT

GCAATTGCTGGTGGGTGGGTGGGCCGCTGGGTTCATGCCTGGCTTTAGCGGAAGCCCTATTGTGGCCCCC
CTCCCTCCCACCCTCCCGACCATGCAGACCTGCTACAACTGCGGGTGTCAGGAGATGGCCATCAAGCGGC
TTGGGGGGCTGAAGGAGGGGCAGCGGCCCTGGTCGGTCAAAGTCTGCAACGACTGCTTGACGACCTGGGT
GAGGACCGTGCATGTGCGGGGACGGCTGGGCACTGGGGACGGATTGATTGACAGGTTGACGGCTGAGACA
GCTCATGTGCGCTCTCTTCTTCCTCCCCGTTCCCCATCCACAGAATCGCGACGTCTCCGCCGCCAACGTG
ATCCGTGTGCTCCTCCTGCTGAAGCTGATGGGCTTCGAGCGGCCAATCAAGCTGCAGCGGCCGCCATGGC
CGCCGGCGGCGGCGGGGCCGGGCTGAGAGCCTGAACGGCGCTAGCAGGGCGTGGGGCTGAGGGTGCACGT
GTTGATTGGCGGCGAGTGACGTGACTAGTTTGTTAGCTGCGGGTTAGCACGGACTGTGCACCCCACCCAA
CCGGCCATGTCCGGATTTGCGGGGATGCCAAAGGCCCCCAACATAGAGGCGTGTGCTTAGTAGGCGCCCG
CGTCAAGGTGGCGGGGTTAATAACGACCCGGGATCAGCCCTTTTCCTGCCATAAGGCAGCCACCTCCTTG
TTAGTGCATC

> CRe_Locus-6, Chr_1 6899776-6899979, correspond to 3805-3992 of CRe-1
TTTTTGCCCGTATGTAGCTGCGGGTTAGCACGGACTGTGCACCCCACCCAACCG
GCCACGTCCGGATTTGCGGGGATGCCAAAGGCCCCCAACATAGAGGCGTGTGCTTAGTAGGCGCCCGCGT
CAAGGTGGCTGGGTTGATAACGACCCGGGATCAGCCCTTTTCCTGCCATAAGGCAGCCACCTCCTTGTTG
CCCGTATGTA

> CRe_Locus-7, Chr_7 619707-624771, corresponding to 2-4882 of CRe-2
TTTTCCCTTGATGGTGTGTGGAGGTGCAAGGGTGCTGTTTATGTCGCCGGTACCCATCGTCACATCC
CTTGCTCTAGGGGCTGAGCCCCCTGAGCGAAGCGATCCAGGGGGGGCGAAGCCCCCCCAGGATTGCCCCT
GTCCGTGCCTGCCTGCCTGCGTTGACAAAAAGTGCCAAACTGGTGCAAACCGCGAGCGCCACGTATTATA
AATTGCTATTCCCTATTGTAGAAAAATAGGCGGCAGGGAAAACTCGGCTGGAGCGAGAAGCGACCTCGCG
AGTCCATGGACATCTTGACTTTCGCGAGGTCGCGAGGTCGCAAGGAAAGCAGCCCTGTCACGCGGATTCC
ACAAAGGAAGAGTTGCCAACTTCAAACTTTCCCCTTCGCACCCTTGTAGCCGTAGCACAGGCCAAACAAT
TCTGAAGTCCGCCCAACCTTGCCCGTGTTAGGGGTGTGGGCCGGCTCCCACACCCCTAACGTTCATAATG
GTTCAGAACAATAGCACCCGTAAGGTGTCGCTGCGAAGCGTTTGCAAGGACGAGGCAACGCTCGCAGCAA
TTAGGTTCAAGGTAGATTTTGTGGCCAGGCCCCTTCGGTACCGCCTGCTGGACTTCCTGCGGCACTTCGT
CGTCTGCCGAACGGACCCCGCCTTCCACTGCGCGGTCTGCGACACCAAGTGGCGGGAGCAGTGCGGACTG
GCTGCGCGGCGTCAAGGACGTAAGCATTAAGTCTAGAGCGCTTTATGCTAGAGCGCATTATGCGCAGGTA
TCATTGACCTGCTGGTTGCTTCCCAATGCCCGCAGTGCGCGAGCGGGCTGACGTCTCGCCACAACAGCAC
GTCTTCCACCGGCGTGTGCCAACCATCGACATCAACCTGCTGCAGGAGTCCGCGGCGCTCATCACAACGT
CCACGCATCGTCGCCAACGCCAAAGCCTCCTGTCGCATTTCTACGATGCGCACTTTGCACCGCTGCTGAG
CGAGGACACGGGTGGCCCGGCCATCGACATCACGGCGCACCACCAGGTACGGGTCACTCCCTGGGACCGC
GGTCGTGAATGCCTCATGTCTTTGACATATGCCCTGTCGTTTCCTGTCGCAGCTGGTGCACCACCTGCTT
CAAGACGTCCTCACCAACCTGGAGCAGCACTACAGGGCGAACTTTCCCAAATGGGTTCGCAAGTACTGTC
GTGCGAAGCTGGAGGTGAACGACGATGAGGTGCCGCTGGGGGGACTAGCGGGCATGGCGCTTGGTCAGCC
GCAGGCGGGCAACGAGGGCGGAGCAGAGACGGACGAGGAGGACGAGGAGGACCAGGTGGGCATTGGGGTG
TGCTCAGCTTCTTTGTAGATGCAGTCTATCTGATTGTATCCTGGGTCAGGCTGGGTTGTGCTCTGAGGCT
GCCGTTGTTTGCCCCCCTGCTAATTAATGATGTCTGTAACTCCCCGTCGGCAACAGGTGCACGTTACAGC
CAAGGCAAGGAAGGCCTTAGCCGGCCTAATGACTGCGGCGCTGATGGGCTGGCGGCAGCTACCACTGACA
GAGGCAGACCTCGGCGGCCTGGCGGTGCCAGATGCGCTGCTGGCTCGTGCCAATGCCACTGTGGCGCCCT
TGGTGGCTGCGTGGACGGCCCGTGGCATCTGGCCTATCCAGCGCGGCAGCCGGGGCCTCCTGCAGCGTGA
GCCGCTCTGCCTGGTCAGTGCTGCTGCCGACCTCCTGTGTGGCCTGCAGCCCATCCGCAGGTGTGCAGTC
CTGGGTGTACATGCGTATCCCTTTGCATGCTGGTATGTGTGCAGAGGCCCAGAGGTCGTGTGTGCGCAAT
CGTGCAGGTGGTTGGCCACTGTCTACGAGCGGCTAAAGACGGCCAATCCACCTGCGCCACCGGGTGAGGT
GCAATCAGCCAACTTCGCCATGAAGCGGGCGCAGACGTACGCGCTGATCATCGAGGCCAGCAGCATGCCG
CTGCACATCCCGCTGACCAACACCATCATCCAGGTGTGTTTTGTGCTTAGTGGCTTGCTTGCTTGATACC
ACCCTCCGAGTTAGTTCGTACAGCAATCCCGTCCAAACAGTAGCCCGTAACCATTGTGCTCATGCCACTG
ACACAGGAACTCCTCGTCAACCACAAACAGCTTGCAGCAGCCGAGTACGCCGCCGCCGGAGCTGAGCTGC
TGGGGTCGAACGCCACGGCTGTTTGGCAGGCGGCCACTGCCGCTGCAAAACACGCTGGTGGTTGGAAAGC
GTTCGCCAATGTGCAGCAAGCAGCAGGCGCGCCGGCGCATCACGACGCGTGGCGGGATTGTGGCTTCGAC
TTCGACTGGCTGAAGCGCCTAGAGCGCGGTGGTGGCACCTTCGCGTACTTCATGCGCACGGATGGCGCGG
CCGCCTGTGTGACGCTTACCGACGCTGCTGGCAGCAGCAGCGACGGCAGCAGCAGCGACGGCAGCAGCAG
CAGCGACGGCAACAGCAGCGACGGCAGCAGCAGCGACGGCAGCAGCAGCAGCGACGGCAGCAGCAGCAGC
GACGGCAACAGCAGCGACGGCAGCAGCAGCGACGGCAGCAGCAGCAGCGACGGCAGCAGCAGCAGCGACG
GCAGCAGCAGCGACGGCAGCAGCAGCAGCGACGGCAGCAGCAGCGACGGCAGCAGCGACAGCGACGGCAG
CAGCGACAGCGACGGCAGCAGCGACAGCGACGGCAGCAGCAGCAGCGACGGCAGCAGCAGCAGCGACGGC
AGCAGCAGCAGCGACGGCAACAGCAGCGACGGCAACAGCAGCGACGGCAGCAGCGACAGCGACGGCAGCA
GCTACCTTGACGGACCGGGAAGAGCGCCAGGGAACAATCCGGTTCCTGGCGTGGTTGCTGCTGGCCAGGC
GCTTGCGCAATACACACCAGATGCCCTCAAGGCCATGCGCGTTGTAGCCGTGGACCCGGGCTGCGTCAAT
TACCTGGTGGCCTGCGTCGACTACCTGGCCGACTTCAGCCCGCACAGCCACCAGCACCCTGCGCACGCCG
TACGGCTGTGGGCCGGCCCGAGAGACATCCCAGAGGCCGAGCGTCAGCAATTGGCGCAAGGCCCTACCCG

GCCTCTGCGAGAGCGGCGCAGGGAGCGGGCAAAGCGCCGGCGGCGGCAGCGGCGGCTTCGTGGGGTCGGG
CGCGCTAATGTGTGGCGGAAGCGCCAGCGGGGGCAGTCGAGGGGGCGCGGGAGGCGAGGGTGTGCCCGTG
GGCGGTGGCGGCGAAAGCCGCGCGGTTGGCGCGGCCGCCGTTCGCGGAAGCAGAGGCATGCGGCGACCAA
GCGGCGCAGGGAGCAGCGCAAGTGCTACGCGCGTGTGTCTGGTGCTGGTTACCGCAAGCGCACTGGCCAA
TCAGAGCGCCAGCGTTGGCAGGAGGCGCGTCTGCAGGAGCAGCCAGCGCTCCAGCGGTGGCAGGCCGGGA
TCCCAAGTGCGCGGGTGGCGTATGCAGCTGACCACACACGCCGCATTCAGTACCTATACGGCGGCAGCGG
TGGGATAGGGCTGTGGCAGCTCCTGCGCCATTACCGGCAGTGGGGCCAGCGGCGGTGGCGCTTGACTGTT
TACATTCGCAGCCAGAAGGTGCGCGTTGGTTGCTAACTCCGTCTGTCCCAGGTTGCAAGCGTGCCCTGTA
CAGCTCATGCAAACAAGTCACCGCACAACGTCATGCCCGTCCAGGTCCTCGAGCAAACGGCGCAGCAGCT
GGCTGGGGGAAGGCCCAAGGAGGAGGTCATCGTCGGCTGGGGCAACGCCAACACTGGCCACGGCGGCTGC
GTCAGCAGGTCAGGCAGCGGCTCTGGTCCTTGGCTAACGCAGCCACTCACTCACCCGGCCACTCGCTCAG
CAACGCACTCACTCACCCACCTTCACTCACCCACTCACCTTCACCCACTCACTCACTTACTTCGCAATTC
AGGTCAGGCAGGGGCCCAAACCGGGCGCTGCTGCGTCTGCTGGTGGACAAGTACGCCCACCTGGTGGTCT
ACGTGGACGAATACTACACCAGCCAGGTACGCTGTGATCTTCACAAAGCAATGCGCACAACGCTCAATGC
ACACGTACCTTACCTGGCCCAAACCTATCTCTGTGTCTGAGCAGGTGTGTGCGAAGTGCGGACGGCGCCT
GCTTGGTAACGGGCAGCGCTGCCTGGAAGTTGTGATTCCGTTTGGTGGCTCGCGTGCCCGCGACGTCCAA
GTGTGCCAACACTGCGGGACTGTGTGGGTAAGTGGGATGGCAGGTTTGGGGCAGGTTTGGGGCGGCAGGT
CGGTTTGGAGATGATGACACGGGATGACACGGGATGACACAGGGCGGTTTGGAGATGATACGGGGTGACA
CACGGGATGACACATGGGATGACACACGCCCTGTTTGCATGTTGAACTGCAGGGCCGCGATGCCAACTCG
GCTACAAACATGCGGCACGCGCTGATGGAGATGCTGCTTGGCAAACCGCGGCCTGCAGCCCTGCGACCTG
CTGGCGGCGGTGGCGGTGCAGGGCCTGGCGGCGGCGGTGGCGGTGGCGGTGGCGGTGGCGGCGGTGCAGG
GCCTGGAGACGGCGGTGGCGGCGGCGGTGGCACCGCGCCTACCGGCGGCAACAGCAGCGGCCACGGCGTT
GGGCCCGGTGGTGGTGGCGGCACGGGCGGCCCTGGGCCCAGCGGCAGTGGCGGCGCGCATGTTCGGAGCA
GAGGGGGCGGGCAAGGCCACGTGGAGGAGGACAGCGCGGCGCCGCCTTCAAAGCGGCGCCGGCGCGCAGG
TTGAGACCTGCCGGTGTTGTCTGGTTAGCGACCTGTGCTGACGAGGATGGCGTAGCGGGCAGTCAGCTGC
AGCGGCAGGGGTGTTTTCCTTGCTGTCTGGTTGGCCTGCTGCCTGCTGCACTAGTGCTTGATAGCTGGGC
GCGACTGGGCACATATGGCGGGCGGTGCCTGTACAAACCGACCCCGGCTATGTCCGGGAGATGAGGCTAG
TATCGGAACCTTCGGCCTCAGACGGAGGACGTGGCGTGGCGGCACAGCCCACTTTTCCCTTGCAAGGGAG
AGCCACCTTTTCTTGTCCCTTGATGGTG

> CRe_Locus-8, Chr_2 4965784 4966955, correspond to 1-321, 3957-4882 of CRe-2

TTTTTGGGCTGAGCCCCCTGAGCGAAGCGATCCAGGGG
GGGCGAAGCCCCCCAGGATTGCCCCTGTCCGTGCCTGCCTGCCTGCCTGCCTGCCTGCCTGCCTGCCTGC
GTTGACAAAAAGTGCCAAACTGGTGCAAACCGCGAGCGCCACGTATTATAAATTGCTATTCCCTATTGTA
AAAAAATAGGCGGCAGGGAAAACTCGGCTGGAGCGAGAAGCGACCTCGCGAGTCCATGGACTTCTTAACT
TTCGCGAGGTCGCGAGGTCGCAAGGAAAGCAGCCCTGTCACGCGGATTCCACAAAGGAAGAGCTGCCAAC
TTCAAACTTTCTGCCTGGAAGTTGTCATTCCGTTTGGTGGCTCGCGTGCCCGCGACGTCCAAGTGTGCCA
ACACTGCGGGACTGTGTGGGTAAGTGGGATGGCAGGTTTGGGGCAGGTTTGGGTCGGCAGGTCGGTTTGG
AGATGATGACACGGGATGACACGGGATGACACGGGATGACACAGGGCGGTTTGGAGATGATACGGGGTGA
CACACGGGATGACACATGGGATGACACACGCCCTGTTTGCATGTTGAACTGCAGGGCCGCGACGCCAACT
CGGCTACAAACATGCGGCATGCGGCGGTGGCGGTGGCGGTGGCGGTGGCGGCGGTGCAGGGCCTGGAGAC
GGCGGTGGCGGCGGCGGTGGCACCGCGCCTACCGGCGGCAACAGCAGCGGCCACGGCGTTGGGCCCGGTG
GTGGTGGCGGCACGGGCGGCCCTGGGCCCAGCGGCAGTGGCGGCGCGCATGTTCGGAGCAGAGGGGGCGG
GCAAGGCCACGTGGAGGAGGACAGCGCGGCGCCGCCTTCAAAGCGGCGCCGGCGCGCAGGTTGAGACCTG
CCGGTGTTGTCTGGTTAGCGACCTGTGCTGACGAGGATGGCGTAGCAGGCAATCGGCTGCAGCGGCAGGG
GTGTTGAGAGGTTTTCCTTGCTGTCTGGTTGGCCTGCTACCTGCTGCACTAGTGCTTGATAGCTGGGCGC
GACTGGGCACATATGGCGGGCGGTGCCTGTACAAACCGACCCCGGCTATGTCCGGGAGATGAGGCTAGTA
TCGGAACCTTCGGCCTCAGACGGAGGACGTGGCGTGGCGGCACAGCCCACTTTTCCCTTGCAAGGGAGAG
CCACCTTCTCTTGT

> Locus-9, Chr_14 2605921-2603969, correspond to 1-699, 3632-4882 of CRe-2

TTTTTGGGCTGAGCCCCCTGAGCGAAGCGATCCAGGGGGGGCGAAGCCCCCCAGGATTGCCC
CTGTCCGTGCCTGCCTGCCTGCGTTGACAAAAAGTGCCAAACTGGTGCAAACCGCGAGCGCCACGTATTA
TAAATTGCTATTCCCTGTTGTAGAAAAATAGGCGGCAGGGAAAACTCGGCTGGAGCGAGAAGCGACCTCG
CGAGTCCATGGACATCTTGACTTTCGCGAGGTCGCGAGGTCGCAAGGAAAGCAGCCCTGTCACGCGGATT
CCACAAAGGAAGAGTTGCCAACTTCAAACTTTCCCCTTCGCACCCTTGTAGCCGTAGCACAGGCCAAACA
ATTCTGAAGTCCGCCCAACCTTGCCCGTGTTAGGGGTGTGGGCCGGCTCCCACACCCCTAACGTTCATAA
TGGTTCAGAAAAATAGCACCCGTAAGGTGTCGCTGCGAAGCGTTTGCAAGGACGAGGCAACGCTCGCAGC
AATTAGGTTCAAGGTAGATTTTGTGGCCACGCCCCTTCGGTACCGCCTGCTGGACTTCCTGCGGCACTTC
GTCGTCTGCCGAACGGACCCCGCCTTCCACTGCGCGGTCTGCGACACCAAGTGGCGGGAGCAGTGCGGAC
TGGCTGCGCGGCGTCAAGGACGTAAGCATTAAGTCTAGAGCGCTTTATGCTAGAGCGCATTGGCTAACGC

AGCCACTCACGTCCTTGGCTAACGCAGCCACTCACTCACCCGGCCACTCGCTCAGCAACGCACTCACTCA
CCCACCTTCACTCACCCACTCACCTTCACCCACTCACTCACTTACTTCGCAATTCAGGTCAGGCAGGGGC
CCAAACCGGGCGCTGCTGCGTCTGCTGGTGGACAAGTACGCCCACCTGGTGGTCTACGTGGACGAATACT
ACACCAGCCAGGTACGCTGTGATCTTCACAAAGCAATGCGCACAACGCTCAATGCACACGTACCTTACCT
GGCGCAAACCTATCTCTGTGTCTGAGCAGGTGTGTGCGAAGTGCGGACGGCGCCTGCTTGGTAACGGGCA
GCGCTGCCTGGAAGTTGTGATTCCGTTTGGTGGCTCGCGTGCCCGCGACGTCCAAGTGTGCCAACACTGC
GGGACTGTGTGGGTAAGTGGGATGGCAGGTTTGGGGCAGGTTTGGGGCGGCAGGTCGGTTTGGAGATGAT
GACACGGGATGACACGGGATGACACAGGGCGGTTTGGAGATGATACGGGGTGACACACGGGATGACACAT
GGGATGACACACGCCCTGTTTGCATGTTGAACTGCAGGGCCGCGATGCCAACTCGGCTACAAACATGCGG
CACGCGCTGATGGAGATGCTGCTTGGCAAACCGCGGCCTGCAGCCCTGCGACCTGCTGGCGGCGGTGGCG
GTGCAGGGCCTGGCGGCGGCGGTGGCGGTGGCGGTGGCGGTGGCGGCGGTGCAGGGCCTGGAGACGGCGG
TGGCGGCGGCGGTGGCACCGCGCCTACCGGCGGCAACAGCAGCGGCCACGGCGTTGGGCCCGGTGGTGGT
GGCGGCACGGGCGGCCCTGGGCCCAGCGGCAGTGGCGGCGCGCATGTTCGGAGCAGAGGGGGCGGGCAAG
GCCACGTGGAGGAGGACAGCGCGGCGCCGCCTTCAAAGCGGCGCCGGCGCGCAGGTTGAGACCTGCCGGT
GTTGTCTGGTTAGCGACCTGTGCTGACGAGGATGGCGTAGCGGGCAGTCAGCTGCAGCGGCAGGGGTGTT
TTCCTTGCTGTCTGGTTGGCCTGCTGCCTGCTGCACTAGTGCTTGATAGCTGGGCGCGACTGGGCACATA
TGGCGGGCGGTGCCTGTACAAACCGACCCCGGCTATGTCCGGGAGATGAGGCTAGTATCGGAACCTTCGG
CCTCAGACGGAGGACGTGGCGTGGCGGCACAGCCCACTTTTCCCTTGCAAGGGAGAGCCACCTTTTCTTG
T

> Locus-10, Chr_1 2796467-2793837, correspond to 171-1385, 3498-4882 of CRe-2

TTTTCTCCAGCTGGTGCAGCGAAAAATAGGCGGC
AGGGAAAACTCGGCTGGAGCGAGAAGCGACCTCGCGAGTCCATGGACATCTTGACTTTCGCGAGGTCGCG
AGGTCGCAAGGAAAGCAGCCCTGTCACGCGGATTCCACAAAGGAAGAGTTGCCAACTTCAAACTTTCCCC
TTCGCACCCTTGTAGCCGTAGCACAGGCCAAACAATTCTGACGTCCGCCCAACCTTGCCCGTGTTAGGGG
TGTGGGCCGGCTCCCACACCCCTAACGTTCATAATGGTTCAGAAAAATAGCACCCGTAAGGTGTCGCTGC
GAAGCGTTTGCAAGGACGAGGCAACGCTCGCAGCAATTAGGTTCAAGGTAGATTTTGTGGCCACGCCCCT
TCGGTACCGCCTGCTGGACTTCCTGCGGCACTTCGTCGTCTGCCGAACGGACCCCGCCTTCCACTGCGCG
GTCTGCGACACCAAGTGGCGGGAGCAGTGCGGACTGGCTGCGCGGCGTCAAGGACGTAAGCATTAAGTCT
AGAGCGCTTTATGCTAGAGCGCATTATGCGCAGGTATGATTGACCTGCTGGTTGCTTCCCAATGCCCGCA
GTGCGCGAGCGGGCTGACGTCTCGCCACAACAGCACGTCTTCCACCGGCGTGTGCCAACCATCGACATCA
ACCTGCTGCAGGAGTCCGCGGCGCTCATCACAACGTCCACGCATCGTCGCCAACGCCAAAGCCTCCTGTC
GCATTTCTACGATGCGCACTTTGCACCGCTGCTGAGCGAGGACACGGGTGGCCCGGCCATCGACATCACG
GCGCACCACCAGGTACGGGTCACTCCCTGGGACCGCGGTCGTGAATGCCTCATGTCTTTGACATATGCCC
TGTCGTTTCCTGTCGCAGCTGGTGCACCACCTGCTTCAAGACGTCCTCACCAACCTGGAGCAGCACTACA
GGGCGAACTTTCCCAAATGGGTTCGCAAGTACTGTCGTGCGAAGCTGGAGGTGAACGACGATGAGGTGCC
GCTGGGGGGACTAGCGGGCATGGCGCTTGGTCAGCCGCAGGCGGGCAACGAGGGCGGAGCAGAGACGGAC
GAGGAGGACGAGGAGGACCAGGTGGGCATTGGGGTGTGCTCAGCTTCTTTGTAGATGCAGTCTATCTGAT
TGTATCCTGGGTCAGGCTGGGTTGTGCTCTGAGGCTGCCGTTGTTTGCCCCCCTGCTAATTAATGATGTC
TGTAACTCCCCATGCCCGTCCAGGTCCTCGAGCAAACGGCGCAGCAGCTGGCTGGGGGAAGGCCCAAGGA
GGAGGTCATCGTCGGCTGGGGCAACGCCAACACTGGCCACGGCGGCTGCGTCAGCAGGTCAGGCAGCGGC
TCTGGTCCTTGGCTAACGCAGCCACTCACTCACCCGGCCACTCGCTCAGCAACGCACTCACTCACCCACC
TTCACTCACCCACTCACCTTCACCCACTCACTCACTTACTTCGCAATTCAGGTCAGGCAGGGGCCCAAAC
CGGGCGCTGCTGCGTCTGCTGGTGGACAAGTACGCCCACCTGGTGGTCTACGTGGACGAATACTACACCA
GCCAGGTACGCTGTGATCTTCACAAAGCAATGCGCACAACGCTCAATGCACACGTACCTTACCTGGCCCA
AACCTATCTCTGTGTCTGAGCAGGTGTGTGCGAAGTGCGGACGGCGCCTGCTTGGTAACGGGCAGCGCTG
CCTGGAAGTTGTGATTCCGTTTGGTGGCTCGCGTGCCCGCGACGTCCAAGTGTGCCAACACTGCGGGACT
GTGTGGGTAAGTGGGATGGCAGGTTTGGGGCAGGTTTGGGGCGGCAGGTCGGTTTGGAGATGATGACACG
GGATGACACGGGATGACACAGGGCGGTTTGGAGATGATACGGGGTGACACACGGGATGACACATGGGATG
ACACACGCCCTGTTTGCATGTTGAACTGCAGGGCCGCGATGCCAACTCGGCTACAAACATGCGGCACGCG
CTGATGGAGATGCTGCTTGGCAAACCGCGGCCTGCAGCCCTGCGACCTGCTGGCGGCGGTGGCGGTGCAG
GGCCTGGCGGCGGCGGTGGCGGTGGCGGTGGCGGTGGCGGCGGTGCAGGGCCTGGAGACGGCGGTGGCGG
CGGCGGTGGCACCGCGCCTACCGGCGGCAACAGCAGCGGCCACGGCGTTGGGCCCGGTGGTGGTGGCGGC
ACGGGCGGCCCTGGGCCCAGCGGCAGTGGCGGCGCGCATGTTCGGAGCAGAGGGGGCAGGCAAGGCCACG
TGGAGGAGGACAGCGCGGTGCCGCCTTCAAAGCGGCGCCGGCGCGCAGGTTGAGACCTGCCGGTGTTGTC
TGGTTAGCGACCTGTGCTGACGAGGATGGCGTAGCGGGCAGTCGGCTGCAGCGGCAGGGGTGTTTTCCTT
GCTGTCTGGTTGGCCTGCTGCCTGCTGCACTAGTGCTTGATAGCTGGGCGCGACTGGGCACATATGGCGG
GCGGTGCCTGTACAAACCGACCCCGGCTATGTCCGGGAGATGAGGCTAGTATCGGAACCTTCGGCCTCAG
ACGGAGGATGTGGCGTGGCGGCACAGCCCACTTTTCCCTTGCAAGGGAGAGCCACCTTTTCTTGTCTCCA
GCTGGTG

> CRe_Locus-11, Chr_5 1801311-1798869, correspond to 2449-4882 of CRe-2

TTTTCGCAGCGACGGCAGCAGCAGCAGCGACGGCAGCAGCAGCGACGGCAACAGCAGCGACGGCAG
CAGCGACAGCGACGGCAGCAGCAGCAGCGACGGCAGCAGCAGCAGCGACGGCAACAGCAGCGACGGCAAC
AGCAGCGACGGCAACAGCAGCGACGGCAGCAGCGACAGCGACGGCAGCAGCTACCTTGACGGACCGGGAA
GAGCGCCAGGGAACAATCCGGTTCCTGGCGTGGTTGCTGCTGGCCAGGCGCTTGCGCAATACACACCGGA
TGCCCTCAAGGCCATGCGCGTTGTAGCCGTGGACCCGGGCTGCGTCAATTACCTGGTGGCCTGCGTCGAC
TACCTGGCCGACTTCAGCTCGCACAGCCACCAGCACCCTGCGCACGCCGTACGGCTGTGGGCCGGCCCGA
GAGACATCCCAGAGGCCGAGCGTCAGCAATTGATGGCGCAAGGCCCTACCCGGCCTCTGCGAGAGCGGCG
CAGGGAGCGGGCAAAGCGCCGGCGGCGGCAGCGGCGGCTTCGTGGGGTCGGCGGGCGCGCTAATGTGTGG
CGGAAGCGCCAGCGGGGGCAGTCGAGGGGGCGCGGGAGGCGAGGGTGTGCCCGTGGGCGGTGGCGGCGAA
AGCCGCGCGGTTGGCGCGGCCGCCGTTCGCGGAAGCAGAGGCATGCATGCGGCGACCAAGCGGCGCAGGG
AGCAGCGCAAGTGCTACGCGCGTGTGTCTGGTGCTGGTTACCGCAAGCGCACTGGCCAATCAGAGCGCCA
GCGTTGGCAGGAGGCGCGTCTGCAGGAGCAGCCAGCGCTCCAGCGGTGGCAGGCCGGGATCCCAAGTGCG
CGGGTGGCGTATGCAGCTGACCACACACGCCGCATTCAGTACCTATACGGCGGCAGCGGTGGGATAGGGC
TGTGGCAGCTCCTGCGCCATTACCGGCAGTGGGGCCAGCGGCGGTGGCGCTTGACTGTTTACATTCGCAG
CCAGAAGGTGCGCGTTGGTTGCTAACTCCGTCTGTCCCAGGTTGCATGCGTGCCCTGTACAGCTCATGCA
AACAACTCACCGCACAACGTCATGCCCGTCCAGGTCCTCGAGCAAACGGCGCAGCAGCTGGCTGGGGGAA
GGCCCAAGGAGGAGGTCATCGTCGGCTGGGGCAACGCCAACACTGGCCACGGCGGCTGCGTCAGCAGGTC
AGGCAGGGGCTCTGGTCCTTGGCTCACTCAGCCACTCACTCACCCGGCCACTCGCTCAGCAACGCACTCA
CTCACCCACCTTCACTCACCCACTCACCTTCACCCACTCACTCACTTACTTCGCAATTCAGGTCAGGCAG
GGGCCCAAACCGGGCGCTGCTGCGTCTGCTGGTGGACAAGTACGCCCACCTGGTGGTCTACGTGGACGAG
TACTACACCAGCCAGGTACGCTGTGATCTTCACAAAGCAATGCGCACAACGCTCAATGCACACGTACCTT
ACCTGGCGCAAACCTATCTCTGTGTCTGAGCAGGTGTGTGCGAAGTGCGGACGGCGCCTGCTTGGTAACG
GGCAGCGCTGCCTGGAAGTTGTGATTCCGTTTGGTGGCTCGCGTGCCCGCGACGTCCAAGTGTGCCAGCA
CTGCGGGACTGTGTGGGTAAGTGGGATGGCAGGTTTGGGGCGGCAGGTCGGTTTGGAGATGATGACACGG
GATGACACGGGATGACACAGGGCGGTTTGGAGATGATACGGGGTGACACACGGGATGACACACGCCCTGT
TTGCATGTTGAACTGCAGGGCCGCGACGCCAACTCGGCTACAAACATGCGGCACGCGCTGATGGAGACGC
TGCTTGGCAAACCGCGGCCTGCAGCCCTGCGACCTGCTGGCGGCGGTGGCGGTGCAGGGCCTGGCGGCGG
CGGTGGCGGTGGCGGTGGCGGTGGCGGCGGTGCAGGGCCTGGAGACGGCGGTGGCGGCGGCGGTGGCACC
GCGCCTACCGGCGGCAACAGCAGCGGCCACGGCGTTAGTTGGGCCCGGTGGTGGTGGCGGCACGGGCGGC
CCTGGGCCCAGCAGCAGTGGCGGCGCGCATGTTCGGAGCAGAGGGGGCAGGCAAGGCCACGTGGAGGAGG
ACAGCGCGGCGCCGCCTTCAAAGCGGCGCCGGCGCGCAGGTTGAGACCTGCCGGTGTTGTCTGGTTAGCG
ACCTGTGCTGACGAGGATGGCGTAGCGGGCAGTCAGCTGCAGCGGCAAGGGTGTTGAGGGGTTTTCCTTG
CTGTCTGGTTGGCCTGCTGCCTGCTGCACTAGTGCTTGATAGCTGGGCGCGACTGGGCACATATGGCGGG
CGGTGCCTGTACAAACCGACCCCGGCTATGTCCGGGAGATGAGGCTAGTATCGGAACCTTCGGCCTCAGA
CGGAGGACGTGGCGTGGCGGCACAGCCCACTTTTCCCTTGCGAGGGAGAGCCACCTTTTCTTGTCGC

> CRe_Locus-12, Chr_2 1847996-1847305, correspond to 4232-4882 of CRe-2

TTTGCACGATCGATGGCAGTACGGCAGTACTCGGCTACAAACATGCGGCACGCGCTGAT
GGAGATGCTGCTTGGCAAACCGCGGCCTG
CAGCCCTGCGACCTGCTGGCGGCGGTGGCGGTGCAGGGCCTGGCGGCGGCGGTGGCGGTGGCGGTGGCGG
CGGTGCAGGGCCTGGAGACGGCGGTGGCGGCGGCGGTGGCACCGCGCCTACCGGCGGCAACAGCAGCGGC
CACGGCGTTGGGCCCGGTGGTGGTGGCGGCACGGGCGGCCCTGGGCCCAGCGGCAGTGGCGGCGCGCATG
TTCGGAGCAGAGGGGGCGGGCAAGGCCACGTGGAGGAGGACAGCGCGGCGCCGCCTTCAAAGCGGCGCCG
GCGCGCAGGTTGAGACCTGCCGGTGTTGTCTGGTTAGCGACCTGTGCTGACGAGGATGGCGTAGCGGGCA
GTCAGCTGCAGCGGCAGGGGTGTTTTCCTTGCTGTCTGGTTGGCCTGCTGCCTGCTGCACTAGTGCTTGA
TAGCTGGGCGCGACTGGGCACATATGGCGGGCGGTGCCTGTACCAACCGACCCCGGCTATGTCCGGGAGA
TGAGGCTAGTATCGGAACCTTCGGCCTCAGACGGAGGACGTGGCGTGGCGGCACAGCCCACTTTTCCCTT
GCAAGGGAGAGCCACCTTTTCTTGTCACGATCGATGGCAGTACG

> Locus-13, Chr_6 6961269-6963621, correspond to 1-375, 2517-4688 of CRe-3

TTTT
TGGGCTGAGGCCCCCGAGCGAAGCGAACCAGGGGGGGGCGAAGCCCCCCAGGATTGCCCCTGTCCGTGCC
TGCCGGTGCGTGCCTGCGTGCCTGCGTGCCATACTGGCACAAACCGCGAGCGCCACGTATTATTACTTGC
TATTCCCTATTGTAGAAAAATAGGCAGCAGGTAAAACTCGGCCGGAGCGAGAAGCGACCTCGCACGTCCA
TGGACTTTAATTGACTGGCGACTGTGGCGACTCGTTGCGAGGTAAAGGGCCCGAGGCGCAGAATTAAGAG
GAAAGTCATACTGGACTACACCAGGCAAAAGACCACCGCGCAAACTAGGTACCAAGTCTACTGAACCAGC
GGGCGCGCGGGCAGCCGAGCGCGCGGGCGTGGCGGCACGCGGGCGCACGGCAGCGGAGGGAGCGGCAGTG
AAGACGATGACCAGCGTGGGCCCCGTGGCGGGCCGGGCCGGGCACCCGGGGACAACCCAGTGCCGGGCGT
GGTCGCGGCTGGCGATCCGCTGGCTGTCTTTGAGCAGGCAACCCTTAGCCCCATCGTGGGCGCATCGTGG
GCGTTGATCCCGGCGTAGGCGACTACCTGGTGGCCTGTGTCGGGTCAATGCCAGGCTTCTCACCGCACAG
CTACAGTGGCCGGCCACGAGCCGCGCACGTCTGGCGTGGCCCAGATGGCCAGGCTCCTGGGCCTGCCCAG

CCCCCACTGCAGCGACGGCGGCGACGGCAACAGCGGCGATGGCAGCGGCAGCAGCGGCGGCAGCGGCGAC
AGCAGCGACGGCGTCGGCGGGGGCAGAAACGGAGGTGGCGGCGCAAGGCGGGCGGGAGGTGGAAGGGCAA
GAGGCGAGGGTGGCGGGGCACGCGCCAGCACACGCATCAGCGTGCCGCAACAGAACGGCGCCGCCAGCAG
CGCGCCTGCTTTACCCGGCTGTCTGGCGCTGGCTACCGCCACTGCACAGGCCAGACAGGCCGTCGGCTGT
GGCTGCAGGCACGGCTAAAGGAGCAGCCGGCGCTCAAGCGCTGTCAGGCGGGGATACCCAGCGCGCGGGT
GGCGTCGGCAGCTGACCATGAGCGGCGAATACGCTACCTCTACTCTGGATGAGGTGGCGTGGACGCGGCG
GCGGCGGCCGGGTCTGCGCCTGCGCCGGTGCAACAGTACGGGCTGTGGCATCTGCTCCGTTTCTACCGGC
AGTGGGGCCAGCGGCGGTGGCGCCTGACTGTCCATGTGCGGACGCAGAAGGTGGGGGTTGAGGGGAGGGA
CTAGCAGATGCATCCACACCAACTGCACTGCGCTGACGCTACACCAATGCCACGGGTACAGGTGCTGGAG
CACACGGCGCAGCAGCTGGCTGGTGGAAGGCCCAAGGAGGAGGTCATTGTTGGCTGGGGCAACGCCAACA
CTGGCTATGGCGGTTGCATCAGCAGGTTGGGGCGGGGCCCAAACCGGGCACTGCTCCGCCTGCTGGTGGA
CAAGTACGCGCACCTGGTGGTGTACGTGGACGAGTTCTACACATCGCAGGCAAGATGAGAGCAGACGGGT
TGGAATGAGTAGACAGGTGGTCGGGTGGACAGGATGGGGCTCGTGTACGGTACAGGTAGCCGGGTAGCCA
GCAAGGTTCACACTGTACTCCTGCCCCCTCCCTCCCCAGGTGTGTGCGAAGTGCGGGCGGCGCTTACTCG
GCAACGGGCAGCGCTGCCTGCAGGCGGTCGTACCATGGGGGGGCTTACGTGCTCACCAAGTCCAAGTGTG
CAACCACTGCGGGACGGTGTGGGTGAGTCCACTTTGTGGGCGGGCGGCTGGCTGGGCGATGGATGGGTGG
CTGGGTGGCGGCTCAGTGGGGCTCAGTGGGTGATGGCTGGCTGGGTGACGGCTCAGTGGGGCTCAGTGGG
GCTCAGTGGGTGATGGCTGGGTGACGGCTGGGTGACGGCTCAGTCGTGGGCTCAGTGGGACTCACTCACT
CTACCCTCCCGACGGTGCAGGGACGTGACGTCAACTCCGCAACCAACATCCGGCACGCGCTGGTGGAGAT
GCTGCTGGGGCACAAGCGACCTGCATCGCTGCAGACTGGCGGCGGCGGCGGCGGTGGCAGCGGCGGCGGC
GGCAGCGGCGGCGGCGGCAGCGGCGGCGGCGGCAGCGGCGGCGGCGGCATGACAAGTAGTGCGGCTGTTG
AGAACTGTGCTGTGCCTGGCTGCGGTTGGCTCTGCTGTGGCATCGCGACTACTAGATAGGCACCTGGTTT
GGCTATGTGCCTGTGGAAACCGACCCCGGCTATGTCCGGGCGATGAGGTTGTTCGGCCTCAGACTGTGGA
GGAGCTCAGCCCCTTTTCCCGTGACCGGGGAACACTTGT

> Locus-14, Chr_1 2931009-2926905, correspond to 615-4688 of CRe-3

TTTTTTACCCCCCGCAGCGTTGGCGCCGGGAGAGTGGCTTGGCACCTGAAGGGCACCAGCGTGAGTATCT
AGCGAAATCAGCGTGGATGGAAACAAAGCTGGTTCAAATCTTCTAAGCTTCTTTGACCTTTGCCAAACGC
AGTGCGCGACCGTTCCGGTGTCCAGATGCTTCCGCGGAAACAGCGGCACAATCGCGGCCACCCGGACATC
AGCCTTGACATGCTGCAGGAGGCATGTGCGCTGGTGTCTACATCCACGCGGCCACAGCAGCGCGAGAGTG
TCATGGCGCACTTTTACGCCATGCACTTTGCGCCGCTCCTGGTCGAGGCTGCGTGCGGCCCGCCCGTGGA
CATCACGGCGCACCACCAGGTATGTTTGCGGGTGGGCGAGCGAAGTGGTGTGAGTGATGCTCACGGCGCA
CCACCAGGTATGTTTGCGGGTGGGCGAGCGAAGTGGCGTGAGTGATGCTTTCTGGTGAGTGATGCTGGCT
GTGACGTTTATTTTGATGCAGCTGGCGCATCATCTTCTCCAGGACGTGCTCACCAACCTGGAGCAGCATT
ATTGCGCCAATTTTATGCGGTGGGTGCGCAAGTGGAGCCGGGCCGTGCTGGAGGTCCCCGATGAGCAGCT
GCCGCCTGCACCGGCTGCCGACGACGATGATGAGGAGGAGGAGGACGACGATAACGAGGAGGAGGAGGTG
CGAAGCGGGACGGGCATGGGCAGTAAGGTATAGCGGATATTGTGCACGTTGTATCTCTTGCGGTCTGTGA
TCTTAATGCGTAAAGTGTACAAAAAGCCTTGCGCCCTCCCGTGCCAGGCTGACGCTGGCGCCAGGGTGTT
CAAAGCCCTGGCGAGCCTGATGACAGCGGCACTGATGGGCTGGCGGGAGCTGCCACTCACCGAGGGTGAC
ATGGGCGGCCTGGCTGTGCCCGCCGGCTTGCTGGATCGCGCCAACGTGAAGGCAACCGCCCTGGTGCAGA
CGTGGAAGGCTCGGGGGTTACATCCTGTTCAAGCGGGCCAGCAGCGTCTACTGGACAGGGACCCCCTGCT
GCTGGTGAGCGCGGCGGCGGACCTGCTGCGCGATATTCTGCCCAGGCACAGGTGCGTCAGGGGCCGCTAC
CTTGGACTGCTGCCATGGGGTGAATGGGTGAGTCTGGATGAGTAGTTGAGTCATGCCCTCCTAAAGCTGC
ATTGCTTTGTGCGCAGGTGGCTCGTCAGCGAGTACCGCCGACTTCGGACGGCCGCTCTGCCACCTCCGCG
AGGCGAGCTGGTGAAGGCCAAGTTTGCTGCGAGGCGGACGAAGCCGTTTTCACTGGTTGTGGAGGCGTCG
CTCATGCCCCTGCACGTGCCCATCACCAACACGGTTCTGCAGGTGGGCTGGGCGGGGGAGGGGAGGGCAT
TGAAGATGCCACACACGGCCTAACTTTGCACGATGCCGTTGTAGGAGGTGCTCATCAACCGCGAAGTGCT
CGTGGTTGCTTGGGAGGCCGCGGCTGCTGCAGAGCGGGATAACGGTGGGGCGGCAGAAGTGCACAAGGCG
GTGCAGAGCGCTGTGGAGAGTGGTTGGCGAGCATTTGCGAACTCAGCCGCGTCGTTTGAACCGTGGCTGC
AGCGGCAGGGGCTGGAGCCAGGGCATGACCCTTGGCTGGTCGCCGGGTTCCAAACCCAGCGCAATGCATG
GGACGGCTGGGAGTTTGCCGGCTTCCTGCGCACGGACGGTGCCGCTGCATGTGTGGCCCTGAAGAAGGCT
ACTGCGGCTGGCGGCAGCGGGACGGACAGCAGCCCGTCGCGCGGGCGCGCGGGCAGCCGAGCGCGCGGGC
GTGGCGGCACGCGGGCGCACAACAGCCCGTCGCGCGGGCGCGCGGGCAGCCGAGCGCGCGGGCGTGGCGG
CACGCGGGCGCACAACAGCCCGTCGCGCGGGCGCGCGGGCAGCCGAGCGCGCGGGCGTGGCGGCACGCGG
GCGCACGGCAGCGGCAGTGAAGACGGCAGTGAAGACGATGACCAGCGTGGGCCCCGTGGCGGGCCGGGCC
GGGCACCCGGGGACAACCCAGTGCCGGGCGTGGTCGCGGCTGGCGATCCGCTGGCTGTCTTTGAGCAGGC
AACCCTTAGCCCCATGCGCATCGTGGGCGTTGATCCCGGCGTAGGCAACTACCTGGTGGCCTGTGTCGGG
TCAATGCCAGGCTTCTCACCGCACAGCTACAGTGGCCGGCCACGAGCCGCGCACGCCTGGCGTGGCCCAG
ATGGCCAGGCTCCTGGGCCTGCCCAGCCCCCACTGCAGCGACGGCGGCGACGGCAACAGCGGCGATGGCA
GCGGCAGCAGCGGCGGCAGCGGCGACAGCAGCGACGGCGTCGGCGGGGGCAGAAACGGAGGTGGCGGCGC
AAGGGGGGCGGGAGGTGGAAGGGCAAGAGGCGAGGGTGGCGGGGCACGCGCCAGCACACGCATCAGCGTG
CCGCAACAGAACGGCGCCGCCAGCAGCGCGCCTGCTTTACCCGGCTGTCTGGCGCTGGCTACCGCCACTG

CACAGGCCAGACAGGCCGTCGGCTGTGGCTGCAGGCACGGCTAAAGGAGCAGCCGGCGCTCAAGCGCTGT
CAGGCGGGGATACCCAGCGCGCGGGTGGCGTCAGCAGCTGACCATGAGCGGCGAATACGCTACCTCTACT
CTGGAGGTGGCGTGGACGCGGCGGCGGCGGCCGGGTCTGCGCCTGCGCCGGTGCAACAGTACGGGCTGTG
GCACCTACTCCGTTTCTACCGGCAGTGGGGCCAGCGGCGGTGGCGCCTGACTGTCCATGTGCGGACGCAG
AAGGTGGGGGTTGAGGGGAGGGGGCCAGCAGATGCATCCACACCAACTGCACTGCGCTGACGCTACACCA
ATGCCACGGGTACAGGTGCTGGAGCACACGGCGCAGCAGCTGGCTGGTGGAAGGCCCAAGGAGGAGGTCA
TTGTTGGCTGGGGCAACGCCAACACTGGCTATGGCGGTTGCATCAGCAGGTTGGGGCGGGGCCCAAACCG
GGCACTGCTCCGCCTGCTGGTGGACAAGTACGCGCACCTGGTGGTGTACGTGGACGAGTTCTACACATCG
CAGGCAAGATGAGAGCAGACGGGTTGGAATGAGTAGACAGGTGGTCGGGTGGACAGGATGGGGCTCGTGT
ACAGGTAGCCGGGTAGCCAGCAAGGTTCACACTGTACTCCTGCCCCCTCCCTCCCCAGGTGTGTGCGAAG
TGCGGGCGGCGCTTACTCGGCAACGGGCAGCGCTGCCTGCAGGCGGTCGTACCATGGGGGGGCTTACGTG
CTCACCAAGTCCAAGTGTGCAACCACTGCGGGACGGTGTGGGTGAGTCCACTTTGTGGGCGGGCGGCTGG
CTGGGCGATGGATGGGTGGCTGGGTGGCGGCTCAGTGGGGCTCAGTGGGTGATGGCTGGCTGGGTGACGG
CTCAGTGGGGCTCAGTGGGGCTCAGTGGGGCGCAGTCGTGGGCTCAGTGGGGCAGTGGGTGATGGCTGGG
TGACGGCTCAGTCGTGGGCTCAGTCGTGGGCTCAGTGGGACTCACTCACTCTACCCTCCCGACGGTGCAG
GGACGTGACGTCAACTCCGCAACCAACATCCGGCACGCGCTGGTGGAAATGCTGCTGGGGCACAAGCGAC
CTGCATCGCTGCAGACTGGCGGCGGTGGCGGCGGCGGCGGCGGCAGCGGCGGCGGCGGCGGCAGCGGCGG
CGGCGGCGGCAGCGGCAGCGGCGGCGGCGCGTGCGCGCATCTTGGTAGCGGAGGGGGCGGGAAAGGCCAC
GTGGAGGAGGAGAGCGCGGCGCCACCCAAGAAGCGGCGCAAGCGCGCAGGCTGAGGAGCCGGCTGGCGGT
GTCTAGGTAGCGGTCCGCGGTGGGGCTGGCAGGGCATGCGTAGCGGCATGACAAGTAGTGCGGCTGTTGA
CAACTGTGCTGTGTCTGGCTGCGGTTGGCTCTGCTGTGGCATCGTGATTGCTAGGCACCCAGTTTGGCTA
TGTGCCTGTGGAAACCGACCCCGGCTATGTCCGGGCGATGAGGTTGTTCGGCCTCAGACTGTGGAGGGGC
TCAGCCCCTTTTCCCGTGACCGGGGAACACTTGTTTACCCCCCGC

> Locus-15, Chr_9 28759-30621, correspond to 3102-4688 of CRe-3

TTTTCTAAACCAGCCCATAGCCGCTGGCTACCGCC
ACTGCACAGGCCAGACAGGCCGTCGGCTGTGGCAGCAGGCACGGCT
AGAGGAGCAGCCGGTGCTCAAGCGCTGTCAGGCGGGGATACGTACCCAGCGCGCGGGTGGCGTCGGCAGC
TGAGCATGAGCTGCGAATACGCTACCTCTACTCTGGAGGTGGCGTGGACGCGGCGGCGGCTGCCGGGTCT
GCGCCTGCGCCAGTGCAACAGTACGGACTGTGGCACCTGCTCCGTTTCTACCGGCAGTGGGGCCAGCGGC
GGTGGCGCCTGACTGTCCATGTGCGGATGCAGAAGGTGGGGGTTGAGGGGAGGGGGCCAGCAGTAGATGC
ACCCACACCAACTGCACTGCGCTGACGCTACACCAATGCCACGGGTACAGGTGCTGGAGCACACGGCGCA
GCAGCTGGCTGGTGGAAGCCCAAGGAGGAGGTCATTACGTACTGGCTGGGGCAACGCCACCACTGGTCAT
GGCCGTTGCATCAGCAGGTTGGCTGGGGCGGGGCCTAAACCGGGCACTGCTCCGCCTGCTGGTGGACAAG
TACGCGCACCTGGTGGTGTATGTGGACGAGTTCTACATATCACAGGCAAGATGAGAACAGACGGGTTGGA
ATGAGTAGACAGGTGGTCGGGTGGACAGGATGGGGCTCGTGTACATACAGGTACCCGGGTAGCCAGCAAG
GTTCACACTGTACTCCTGCCCGCTCCCTCCCAGGTGTGTGCGAAGTGCGGGCGGCGCTTGCTCGGCGACG
GGCAGCGCTGCCTGCAGGCGGTCGTACCATGGGGGGTGGGGCTCACGTGCTCACCAAGTCCAAGTGTGCA
ACCACTGCGGGACGGTGTGGGTGAGTCTACTTTGTGCGCGGGCCGGCCGCGGCTAGGTGATGGCTGGTGG
CTGGGTGACGGCTCAATGGGGCTCAGTGGGGCTCAGTGGGGCTCAGTCGTGGGCTCAGTGGGGCTCAGTG
GGGATGACACGTGGATGACACGGCTCAGTGGGTGAGGGCTCAGTGAGGCTCAGTGGGGCTCAGTGGGGCT
CAGTGGGGCTCAGTGGGTGATGGCTGGCTGGGTGATGCATGGCTCAGTGGGGCCCAGTGGGGCTCAGTCG
TGGGCTCAGTGGGGCTCAGTGGCAATGACACGTGGATGACACGGCTCAGTGGGTGAGGGCTCAGTGGGGC
TCAGTGGGGCTCAGTGGGGCTCAGTCGTGGGCTCAGTGGGGCTCAGTGGGTGATGTCTGGGTGACGGCTG
GGTGATGGCTCAGTCGTGGCCTCGTGGGCTCAGTGGGACGCACTCACTCTACCCTCCCGACGGTAGGTGC
AAGGACGTGACGTCAACTCTGCAACCAACATCCGGCACGCGCTGGTGGCGATGCTGCTGGGGTGCAAGCG
ACCTGCATCGCTGCAGACTGGCGGCGGCAGCGGCGGCGGTGGCGGCAGCGGCAGCGGCAGCGGCAGCAGC
GGGGGCGCGTGCGCGCACCTTGGTAGCGGAGGGGGCGGGAAAGGCCACGTGGAGGAGGAGAGCGCAGCGC
CGCCCAAGAAGCGGCGCAAGCGCGCAGGCTGAGGAGCCGGCTGGTGTCTAGGTAGAGGTCCGCGGTGGGG
CTGGCAGGGCATGCGTAGCGGCATGACAAGTAGTGCGGCTGTTGACAACTGTGCTGTGTCTGGCTGCGGT
TGGCTCTGCTGTGGCATCGCGACTGCTAGATAGGCACCTGGTTTGGCTATGTGCCTGTGGAAACCGACCC
CGGCTATGTCCGGGCGATGAGGTTGTTCGGCCTCAGACTGTGGCGGGGCTCAGCCCCTTTTCCCGTGACC
GGGGAACACTTGTCTAATCCAGCCCATAGCCG
