# Supplementary material for: Homologues of bacterial TnpB_IS605 are widespread in diverse eukaryotic transposable elements
Source: Mob DNA. 2013 Apr 1;4:12. doi: 10.1186/1759-8753-4-12 (PMC3627910; doi:10.1186/1759-8753-4-12)
Supplement: Additional file 9 — Alignment of the RepHel proteins of Helitron1 and Helitorn2 groups. [file 1759-8753-4-12-S9.pdf]

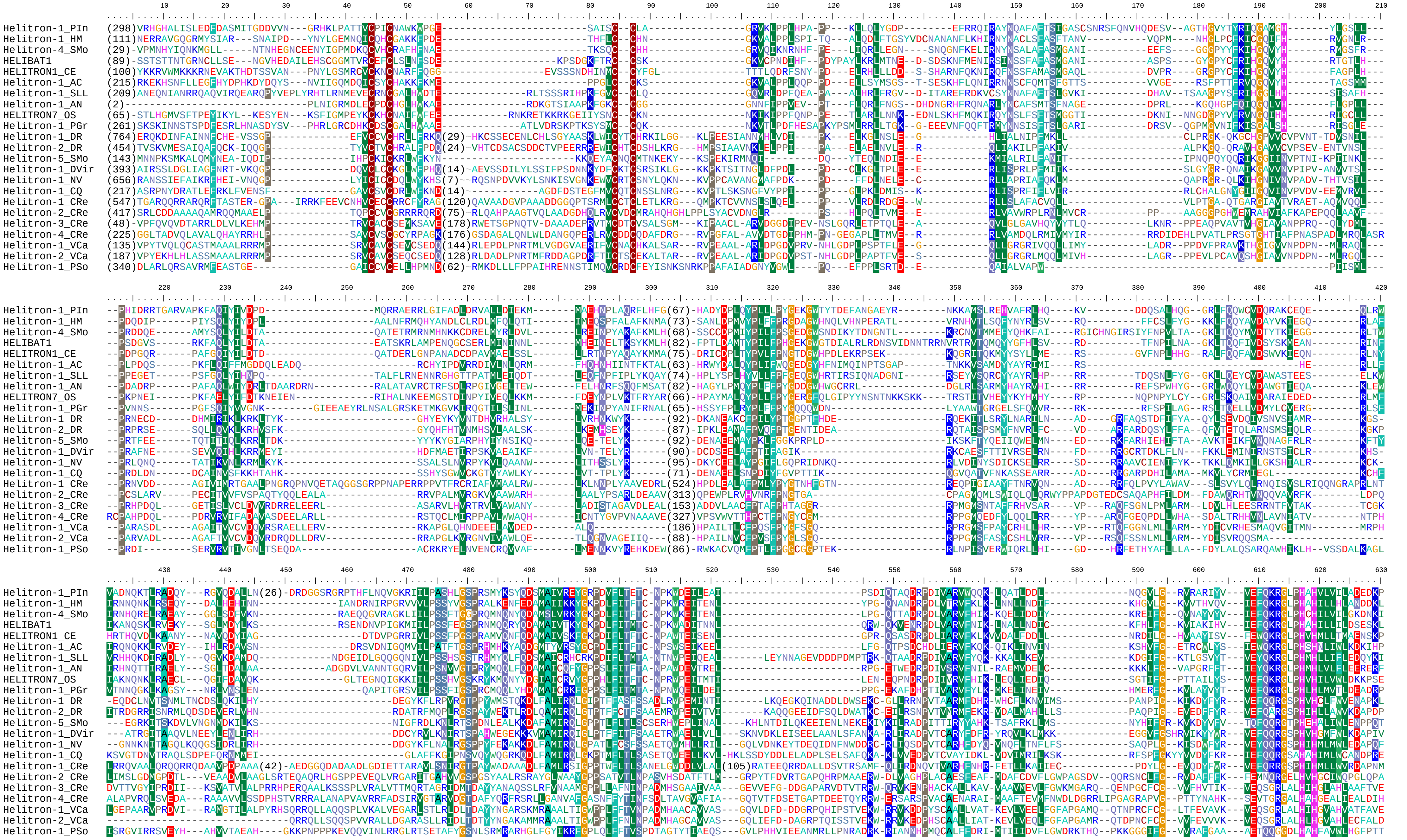

Helitron-1_PIn
Helitron-1_HM
Helitron-4_SMo
HELIBAT1
HELITRON1_CE
Helitron-1_AC
Helitron-1_SLL
Helitron-1_AN
HELITRON7_OS
Helitron-1_PGr
Helitron-1_DR
Helitron-2_DR
Helitron-5_SMo
Helitron-1_DVir
Helitron-1_NV
Helitron-1_CQ
Helitron-1_CRe
Helitron-2_CRe
Helitron-3_CRe
Helitron-4_CRe
Helitron-1_VCa
Helitron-2_VCa
Helitron-1_PSo
10
210
220
420
430
630

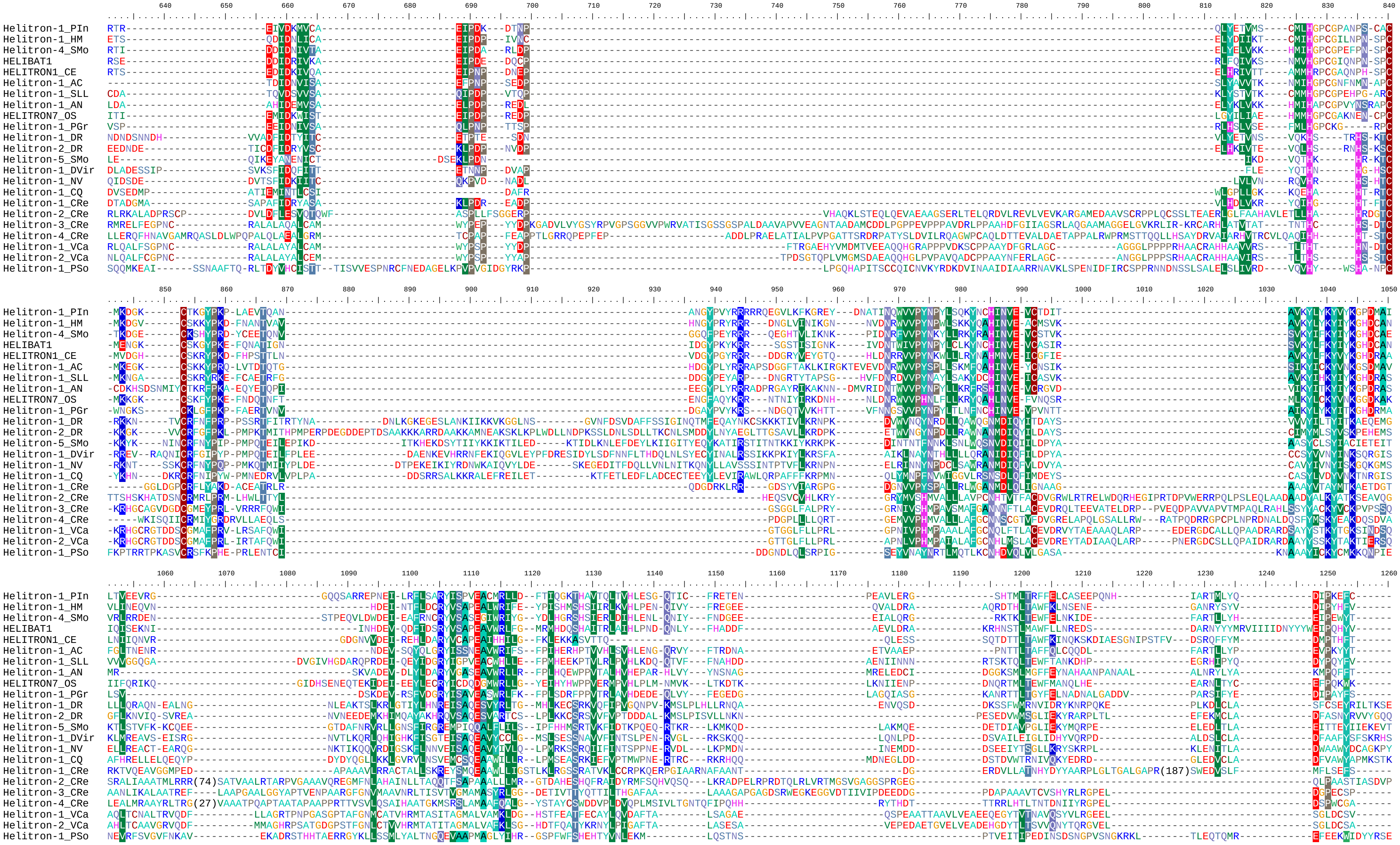

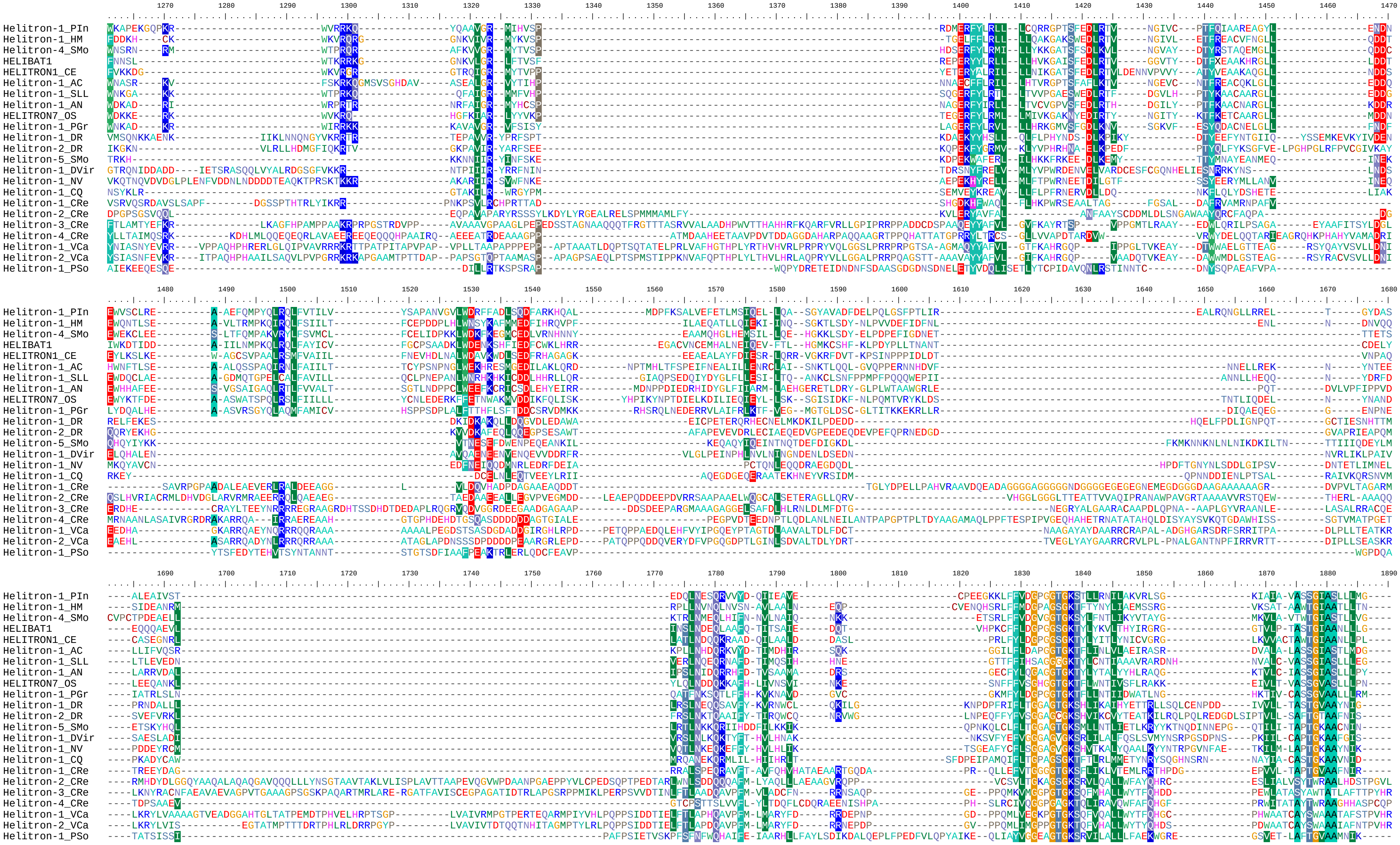
Helitron-1_PIn
Helitron-1_HM
Helitron-4_SMo
HELIBAT1
HELITRON1_CE
Helitron-1_AC
Helitron-1_SLL
Helitron-1_AN
HELITRON7_OS
Helitron-1_PGr
Helitron-1_DR
Helitron-2_DR
Helitron-5_SMo
Helitron-1_DVir
Helitron-1_NV
Helitron-1_CQ
Helitron-1_CRe
Helitron-2_CRe
Helitron-3_CRe
Helitron-4_CRe
Helitron-1_VCa
Helitron-2_VCa
Helitron-1_PSo

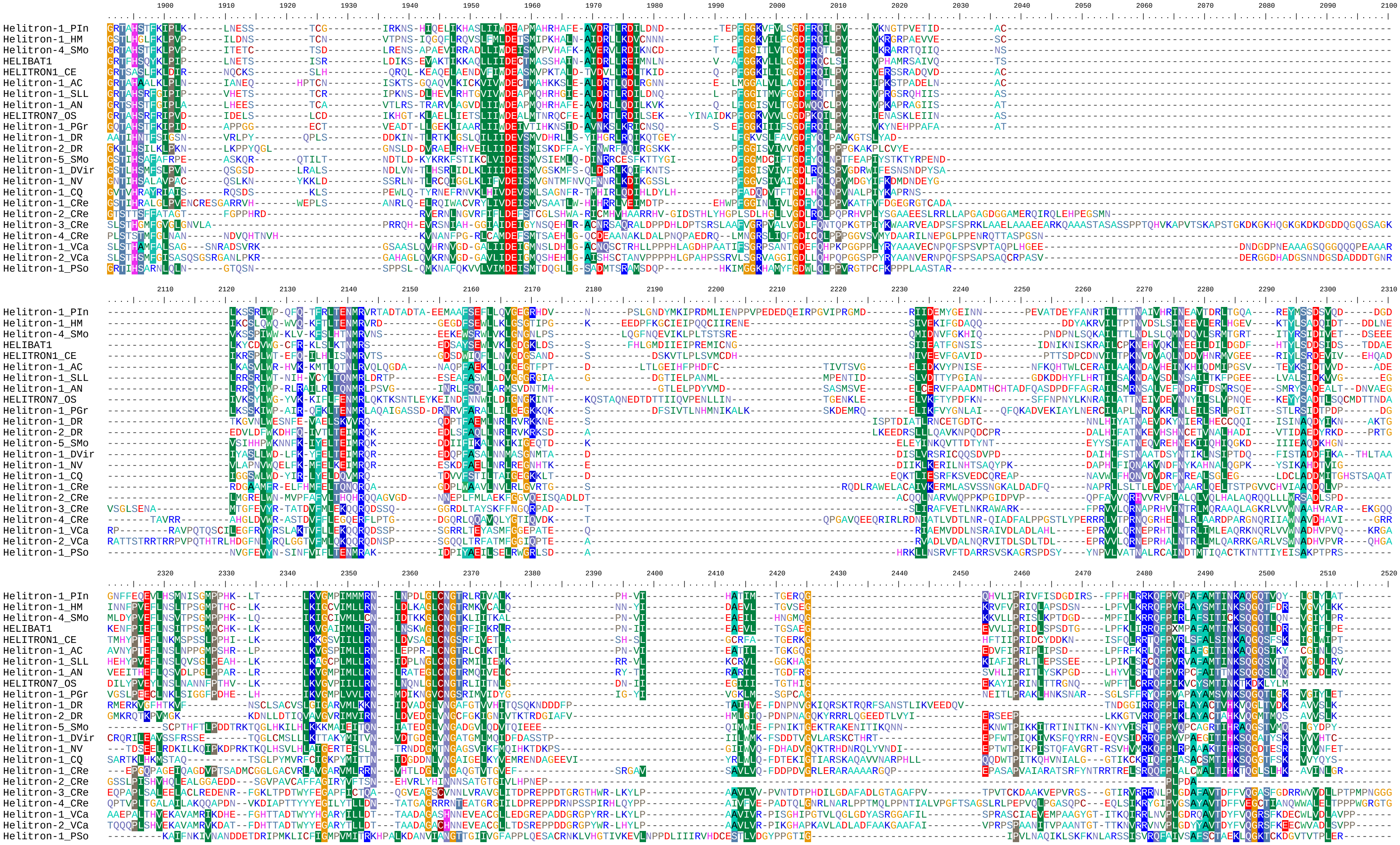

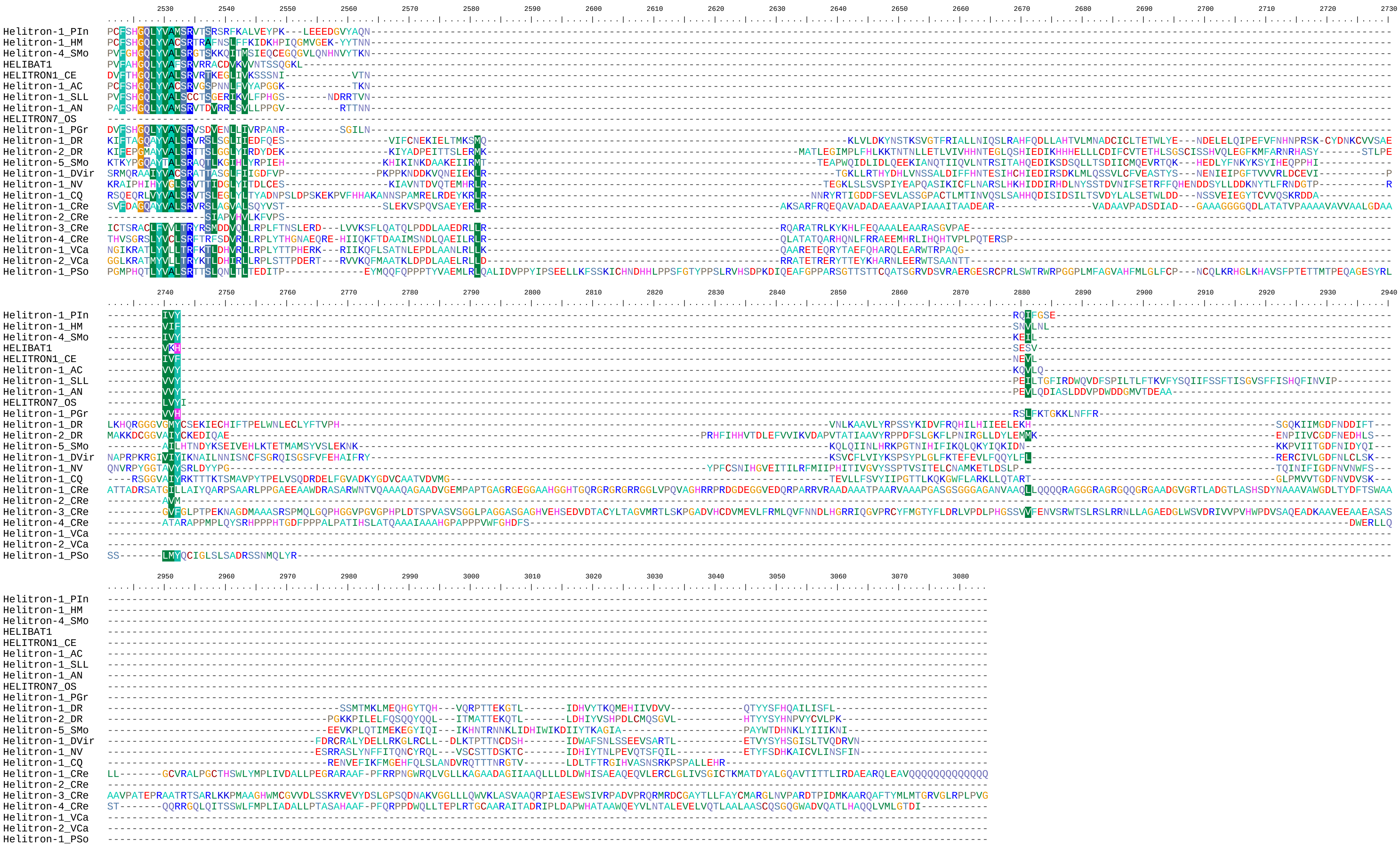

2530 2540 2550 2560 2570 2580 2590 2600 2610 2620 2630 2640 2650 2660 2670 2680 2690 2700 2710 2720 2730
2740 2750 2760 2770 2780 2790 2800 2810 2820 2830 2840 2850 2860 2870 2880 2890 2900 2910 2920 2930 2940
2950 2960 2970 2980 2990 3000 3010 3020 3030 3040 3050 3060 3070 3080
Helitron-1_PIn
Helitron-1_HM
Helitron-4_SMo
HELIBAT1
HELITRON1_CE
Helitron-1_AC
Helitron-1_SLL
Helitron-1_AN
HELITRON7_OS
Helitron-1_PGr
Helitron-1_DR
Helitron-2_DR
Helitron-5_SMo
Helitron-1_DVir
Helitron-1_NV
Helitron-1_CQ
Helitron-1_CRe
Helitron-2_CRe
Helitron-3_CRe
Helitron-4_CRe
Helitron-1_VCa
Helitron-2_VCa
Helitron-1_PSo
