# Supplementary material for: Homologues of bacterial TnpB_IS605 are widespread in diverse eukaryotic transposable elements
Source: Mob DNA. 2013 Apr 1;4:12. doi: 10.1186/1759-8753-4-12 (PMC3627910; doi:10.1186/1759-8753-4-12)
Supplement: Additional file 10 — Alignment of the Sola2-Tpases. [file 1759-8753-4-12-S10.pdf]

Additional file 10

```
                            10        20        30        40        50        60        70        80        90       100       110       120
                    ....|....|....|....|....|....|....|....|....|....|....|....|....|....|....|....|....|....|....|....|....|....|....|....|
Sola2-2_HM_1p       (371)--------IKTARHLKQMHGIMAEPSKKEGRSISEKVKNLVHEFYQSDEYSRMCPGKKQYVSIQ-VDGKRQHFQ----KRLLLLNIKELYLEFVK---------ISLDVK--IGF
Sola2-5_HM_1p       (184)--------IQNAKLLRDQKGIIAYPNVVERHRISKDVIELVKLFYCDDEYSRQMPGKKDCVSVG----KKKYMS----KKLIMCNLKELYVAYKI---------KYPDHK--IGF
Sola2-1_HM_1p       (166)--------AKMARQIFNKKGLLAISPLYKGKVLHKEIEDSVKLFYDSSDLCRTMSGKKDYVSIQ----KNIHKQ----KKLLLCNLKELYVLYKE---------NNPEIQ--ISY
Sola2-4_NVi_1p      ~~~~~-------------------------------------------------------------------------------------------------------------------
Sola2-5_AAe_1p      (287)--------VRQVKQLVNEQGILCTTRARSGHGISETDKQVVIDFFDSDDISRPMPGANDFVSEY-RNGSKEQVQ----KRLLMMSLKEAYMIFEE---------RCQNVE--IGF
Sola2-6_AAe_1p      (263)--------ATEAKQLLNKSGVHSTIGPRATTGLDENIKHAVMQFYEDDETSRAMPGQRDCVTIR-KDGKRQAVQ----KRLMTTTLREAYNRFQE---------LNTEIQ--IGF
Sola2-2_AAe_1p      (210)--------ITESK--KYNLGIQPLS-KVGRPSHGSDVQDIVSNFYLRDDISRPFPGLKDTISIKLPNGVRQNVQ----KRLLLDPLDXLYKQYLE---------TCKSEQESVSF
Sola2-3_SP_1p       ~~~~~-------------------------------------------------------------------------------------------------------------------
Sola2-1_SP_1p       (136)-----------------LPSASAEKRKTGPPKVSEDTVKVIEDFYERDNVSRQAPGRKDVVTIW-NADGKKKMQ----ARHLTASIKETYAFFCE---------IYPEVH--VGK
Sola2-2_SP_1p       (119)----------------------SDYSQNGPTKVTDELCEAISTFYCRDDISWQSPNRKDVVISRKEDGTKEYIQ----KRFLIMTLKECHNLYLE---------ENPDQS--VGL
Sola2-1_BM_1p       (164)------------RKLAKYRMRLHRIKKGQKRGNTVLVKRKIHDFFIDDEHSRLTAGKKETITRR-----KVKKQ----IRLLNDTILNLHKIFNN----------KTGLN--ISY
Sola2-1_Hrobusta_1p ~~~~~-------------------------------------------------------------------------------------------------------------------
DFa-2               (131)AKQYGPGRNIRAEEYTQFCQALGRSDINKTDANKKKIMEDIKEFYAQTTITEPSSKCSTKNQK--DANNKTVRVSTPIRKLLCPSIAQAYAYYVEFIAKKYKDCSDNDEKTPIHR
PPa-1               (171)VHKYGAGRLINAEEISNWRSKFTHNGQMTRRQRTTMLRTAVEGYFNMKDITEESSKNMRLYSKELDADGKRAFELVPIKNLALGNINTCFTDYLK------KSEVDQVKYPLMGR

                           130       140       150       160       170       180       190       200       210       220       230       240
                    ....|....|....|....|....|....|....|....|....|....|....|....|....|....|....|....|....|....|....|....|....|....|....|....|
Sola2-2_HM_1p       SKFCELRPKWCIPVGGASGLHSVCVCEYHQNAKLLALKI------PDISDYKN-------------------LLTLVVCD--LTNRDCMLHCCDNCPD--NDTLKEYLTTLFDKH-----
Sola2-5_HM_1p       SKFCSLRPKWCI-LAGPKGTHSVCVCTIHQNVKLMLSAV------GLETSYHE-------------------IIEKIVCS--RKSKVCMIHRCNSCPG--IQAAQIYFQQYLTQNDDPKE
Sola2-1_HM_1p       SKFASLRPKWCI-LPGANGTHSVCVCCYHQNAILLVDAL------NIGLKYKD-------------------LLSKTVCS--VENKECMLAQCDNCPG--KETLTKYMYEIFGEY-----
Sola2-4_NVi_1p      ----------------------------------------------------K-------------------LTKMVKLS--MFQKDCYYVILKYCIELLKKNVHNFLSALVNSP-----
Sola2-5_AAe_1p      TLFTMLRPKHCR-LLDSTGIHNVCVCTYHENVKLMLNSL------GIVSQAEI--------------------CEKLLCDVLVRTTDCFFRECEKCNS--KENITGELISLLEES-----
Sola2-6_AAe_1p      SSFAKLRPKNCK-LLTSSGTHNVCVCTIHENVNLITHSLKKY---GLSNELKV-------------------FTDSLTCE--NATVDCFLRRCENCID--TTSLEKKLLEEMDEK-----
Sola2-2_AAe_1p      TSFWKLKPKQCV-YTKDSSAMNVCVCMIHENMKFMVDALKKTNCFEVHNTEKKTKKLIALKFIIQKKKLNTFLTSQMICP--DSTGDCYLRSCEDCKL--KKL--DFVANRLDEN-----
Sola2-3_SP_1p       ------------------------------------------------------------------------------------------------------------------------
Sola2-1_SP_1p       SKFAECRPKHV--LLSQKLPHNVCLCRYHENAINAFTALHKAQP-EFPAYTRD-------------------LPALLLCK--QPSRECWMNECAECKD--GAGFSKAFS--FEGS-----
Sola2-2_SP_1p       SKFCSLRPCHV--FLSRDFPHNVCICKYHENIKLLLECIDKSVARKIPTHFRD-------------------FIATVVCD--QEKENCMFGKCSACRS----KFDDTYC--FPEE-----
Sola2-1_BM_1p       ETFRRHRPFWV--IFPKTTSRNTCLCRQHANNDYIASALHQAKIISFSNATD--------------------VARSLCCDN-ILRVSCLERTCTLCFE-------------------KTL
Sola2-1_Hrobusta_1p ------------------------------------------------------------------------------------------------------------------------
DFa-2               STFYKFVPSDVKIFNNRT------------------------------------------------------------------------DTCPYCHL--YDVLKAKAEKTT--------
PPa-1               SAFYSFVPEYVREMKNRT------------------------------------------------------------------------DMCPMCVQ--YQALESK-------------

                           250       260       270       280       290       300       310       320       330       340       350       360
                    ....|....|....|....|....|....|....|....|....|....|....|....|....|....|....|....|....|....|....|....|....|....|....|....|
Sola2-2_HM_1p       ------SIEEINFNQ----WQKANKKHDIVPVT--------LPVDD----FIEKACQQIDQLRDHHYIAKNQAA-----------------------YLQHLKI----------------
Sola2-5_HM_1p       QYDSDENEQSVDFKQ----WTTTDRT-ELLTIQ--------LPLNE----FIELLCEKLDKITSHSFIAKSQSS-----------------------YLKHLKE----------------
Sola2-1_HM_1p       -----EDDFEIHYKQ----WQTTDRA-TLLSLT--------ADVST----FVELLVSCFEKLQAHSFIAHSQSQ-----------------------YLNQLKQ----------------
Sola2-4_NVi_1p      -----------NYVQNGVFW----------------------PVDD----FMSELSLSLKKLIPYHFIAKNQSK-----------------------YIKDQKN----------------
Sola2-5_AAe_1p      ------DKEDVVFQQ----WLTTDRC-NLETII--------KPVEE----FVTYFVEKVDKLITHDFICKEQSS-----------------------FLRNKKKLLKTRLMILFAKNSLL
Sola2-6_AAe_1p      ------FVDEIIFEQ----WVTTDRC-DIETFT--------KQKEE----FVSYFIQKLEKLIPHDLIKKEQST-----------------------FLKNKKK----------------
Sola2-2_AAe_1p      ------NVEEVKYCF----WIISPRC-EIINKE--------ENVND----FIENLKNLTEKFLVHQFKVDKQNX-----------------------FIRAKKE----------------
Sola2-3_SP_1p       ------------------------------------------------------------------------------------------------------------------------
Sola2-1_SP_1p       ------LNSPCSWLQ----WETDKDGRMMKIQE-------EGTVGE----LVQHITAILPQFLQHCYVKREQAS-----------------------AYNAQRE----------------
Sola2-2_SP_1p       ------ADDEAEWIQ----WITVEGK-ATKSIQ-------RGTLSG----CIKSIRQQLPGFLIHTFIKRKQSA-----------------------HFEREKD----------------
Sola2-1_BM_1p       DYTVINGNDTILYQR----WVTKKVPQIIKGNEKLCQKTLKECVRTSHQLLVNKFNISLSTFMQHLANIMNQYK-----------------------AIRYIKQ----------------
Sola2-1_Hrobusta_1p ----------------------------------------------------------------------------------------------------LLKE----------------
DFa-2               --------------------------------------------------LTEKEDTDFKTCKIHKEFVEKRKK-----------------------DFEKKKE----------------
PPa-1               ---------RIKFGQ-----------------------------------LSDQDHKLFEILTIHKMFVQQRKNDYLTCVIIILLIHLVNDLLMKFFFFFIXKN----------------

                           370       380       390       400       410       420       430       440       450       460       470       480
                    ....|....|....|....|....|....|....|....|....|....|....|....|....|....|....|....|....|....|....|....|....|....|....|....|
Sola2-2_HM_1p       -------TL-ASNHILILLDFAENYSF-LIQDAVQGFHW--NNSQATLHPFVIYFMEE----NKIKCKSICIISDNLQHNTNSVHCFIHEVLKHLKNL-IPYFNHCIYFSDGASSQYKNY
Sola2-5_HM_1p       -------TI-SIDEAIILADFAENYTF-VVQDEIQSYHW--SKSQCSLHPVVIYYKKV-----KLEISSFCVISDDLSHDVGFINEVMHKTINHIKTHLCPAITKIHYFSDGCAGQYKNC
Sola2-1_HM_1p       -------NM-DQSNIIIIGDFAENYTF-VVQDEIQSYHW--NTQQCSLHPLVIYYKDD---KGVLKHISYCFISDDITHDVTYVYKIFQLIIPILKTK-FSNLSKLHLFTDGCAGQYKNC
Sola2-4_NVi_1p      -------NL-QENEGLLEMDYSENFAY-VVQDAAQQFHF--NNDQCTINVAVLYYRED----NETRHCSFIALSECTTHDTAAVYILQEKIISEIKKR-FPKMNKIIYVTDGAKQHYKNR
Sola2-5_AAe_1p      FYETKKNSL-KQGEILVISDFSENYSF-IIQNAAQGYHW--NNSQATIHPFEIYHKKD----NKLENVSFIIISEVLTHDTVAVQLFISKLINFVKQK--INFSKVIFMSDGAAAHYKNK
Sola2-6_AAe_1p      --KIKKNNL-QEREFVAICDFSENYTF-VLQDEVQSHHW--NAQQATIHPFAIYFKEN----GMLNHLSFVVISEDLRHDSISVNLFISKMINFIRQEKHLNLNKIYFMSDGAASQYKNR
Sola2-2_AAe_1p      -------SLVENKEIMCQMDFAENYSC-VIQDSIQSHYF--VRPQVTIHPFVIYYKDK----SSIKVLNFVVIADIKKHNTTSVYAFQTKLISRLKNK-FPELEKIIYLSDGCGEQYKNK
Sola2-3_SP_1p       ----------DNEVALLQVDFSENYTC-VHQDEIQSAHW--RQRQVSLFTAAIWNKGL--------MQSHVVASDNLTHSKETIMAYMDTLPDPLPHG----VKLVYVWSDGPASQFKNR
Sola2-1_SP_1p       --KVSPESH-DTHSALLQVDFSENFTC-VAQDEVQSAHW--NQRQVSLFTAALWYSGS--------LHSHILASDDLTHSKETIIAYLDVLLGYLPDT----VTSISIWSDGPASQFKNR
Sola2-2_SP_1p       -------KA-DGRHIVVQVDFAENYSI-IHQDEIQSGHW--NHEQVTIFTGCANIGNG-------KVESFAIVSDDLVHGKYQVATFLEHIIMELKKS-CPEMETVSFFSDGAASQFKQR
Sola2-1_BM_1p       -------NL-SPSQSLLHIDFSEKYSC-KYGSEVQSAHFGGSKSQLSLHTCVYYSVDSQPPTNLIKTTSICTVSENLRHDPVLICAHLKPVIEKIKLI-TPDLTELHILSDGPATQYRNK
Sola2-1_Hrobusta_1p -------SL-QHSEAVLHIDFSENYGF-KNASAIQTCHFGASNQQATIHTGVLYKYNG--------LISFASISESLRKDPSAIWAHIEPILQNLRES-YPGITTLHFFSDGPTTQYRNK
DFa-2               -------SL-TKDQVIVLMDFKENIALGMGPEELSQNFY--GKPQRSILGMVIYHRDP--ATNEVVKEHFDVFSDVLNHCSSYVIHSIKLFFSTSELFKARHFSQIFFWSDG-AKHFKSA
PPa-1               -------EL-KAGECIILCDFKENISL--GKSKVQVSHEFYQQPARTFFGMVLFYKQG----DKLKIHYYDCLSQVLNHCSTFAISCLDKLINDPLFT-SLNINKITFWCDN-ARHFKSN
                                       *                                                                                          *
                           490       500       510       520       530       540       550       560       570       580       590       600
                    ....|....|....|....|....|....|....|....|....|....|....|....|....|....|....|....|....|....|....|....|....|....|....|....|
Sola2-2_HM_1p       KNLSNLCHHKQ------------DHD-VS-----AEWHFFATSHGKSACDGVGGTVKRLVARASLQ-SLVDPINSSKKMYDWCRQN------IDGIHFQHISNDAVTLHCMKFKLE--ER
Sola2-5_HM_1p       KHFYNLCHHAQ------------DFS-VQ-----CIWNFFATSHGKSPCDGIGGTVKRLVSTASLQSPTTGQILSSQAMFEYCQKS------ISGITFVYVTAEEMEL--VRIKLA--DR
Sola2-1_HM_1p       KSFYNLCQLES------------EFS-LK-----VEWNFFATSHGKSPCDGIGGTVKRLTAQESLKRPYRNQILTSEAMYEFCIEK------IKVVNFIYIKGIDLQL--QREEQK--ER
Sola2-4_NVi_1p      YQMMNLVNHES------------DFQ-IM-----ADWHFHATAHGKGSCDGVGAVLKREATRTSLQAKATEAILNSKQLYDWARQK------FQTITFFHYSKKDYEK--TSRSLK--KR
Sola2-5_AAe_1p      KKIASLCNFKK------------IYG-LE-----AEWHFFATSHGKGPCDAIGGTLKRMAKRASLAKDYGNTIATPRELFDWAVKQ--TDTCITKLNFCYISNEQYVK--MSEELM--EL
Sola2-6_AAe_1p      KNFSSLCQFKK------------NYD-ID-----VEWHFFATSHGKGPCDALGGTIKRMATRASLAKEREHPIKNAKELFDWAQKR--KEEQLTQIFFSYATTTEYEH--IKEQLN--EQ
Sola2-2_AAe_1p      SNFKNVCNHEN------------DFK-IR-----AEWHFFPTSHGKGPCDGIGGNIKRMARDASIRKSA--EINNAKQFFDWAVSQKVKDQFKKDWEFIYATENDYSE--AEKLLQ--ER
Sola2-3_SP_1p       FIAAAIDALQK------------KHN-VQ-----VQWNFFCTSHGKGPVDGIGGSVKRHMWKKVSTRKS--IITDTASFTCACADM-------SGVAVRELTDDEIQERNQMLDLK--SV
Sola2-1_SP_1p       FVAAALCTLQE------------AHK-IQ-----IKRNFFSTSHGKGPVDGIGGSAKRFVMQRVLSRQD--IVADASSFVLAASFM-------ENVCVTEVKSTEIAKRNEKLKLK--DV
Sola2-2_SP_1p       FLFENLTYFSE------------MYS-IQ-----TSWNFFATSHGKGSVDGIGGQIKRLVFTACKTGA---FITDPKSFATEAKKH-------TKIKVMFVPATDVDA--RKDVLD--AR
Sola2-1_BM_1p       TMFHMLANYVS------------KISNVET----IVWHFSEAGHGKSAPDGVGGCVKRTCDKAVANGQ---DISGIDSFVDCVKGT------CKGIDIIRINN-------DVSDIQ--KI
Sola2-1_Hrobusta_1p QNFYLLSTQIY------------KLGFTD-----ASWNFFEAGHGKGAPDAIGGALKRRADDKVNMGC---DITTAQSLFKVMSES------DSQIKLYYIEGSDIGR--ITSYCH--QS
DFa-2               EILSFCIDLKK------------SPS---QSLNRVEWQFSAQYHGKSDVDRHFADLSNITAQQSKCYD----IYTGKDFIKGGDRC----------------------------------
PPa-1               EILSYMHTLSKLQVADPNTPNKTKRKYL------VELNTFAPYHGKSECDQHFSMVSRAIADKSSFKD---SIYTDQDVINAIKSTTDNNNYQRTYKLTSESEKEAVTIKVTVEIHNPKE
                                                                     *
                           610       620
                    ....|....|....|....|....|....
Sola2-2_HM_1p       YATC--STIPGTRNHHCFIPQSLT(131)
Sola2-5_HM_1p       LLIA--TTIPGTRSFHQFIPSSHS(124)
Sola2-1_HM_1p       YTGV--TTLPGTRSFHQFIHLGDN(133)
Sola2-4_NVi_1p      FSAA--PSVTKISEGHAFLVSPDK(19)~
Sola2-5_AAe_1p      FDKV--KTVPGTQKYHCFMPISDT(25)~
Sola2-6_AAe_1p      YSKA--ITIQGTQKYHSFIPVSVD(23)~
Sola2-2_AAe_1p      FSNL--VPIPGTKKYHSFIPKDER(44)~
Sola2-3_SP_1p       FDSA--SPINGISRMHFMKHVNNE(103)
Sola2-1_SP_1p       FIQA--KPITGIGKMHFLKVVKDE(101)
Sola2-2_SP_1p       WANV--SALPNTHNTHVVVPLRKH(186)
Sola2-1_BM_1p       ADANKVRPFKGTLKIHQITWSSKS(111)
Sola2-1_Hrobusta_1p L-----TAIAGTMKVHQLYTDTEL(196)
DFa-2               ---------YSFSDYNEFVFTNK-(386)
PPa-1               LKQKEIKIFPGFTEYHRFQFLDNR(547)
```
