# Supplementary material for: Homologues of bacterial TnpB_IS605 are widespread in diverse eukaryotic transposable elements
Source: Mob DNA. 2013 Apr 1;4:12. doi: 10.1186/1759-8753-4-12 (PMC3627910; doi:10.1186/1759-8753-4-12)

Additional file 11

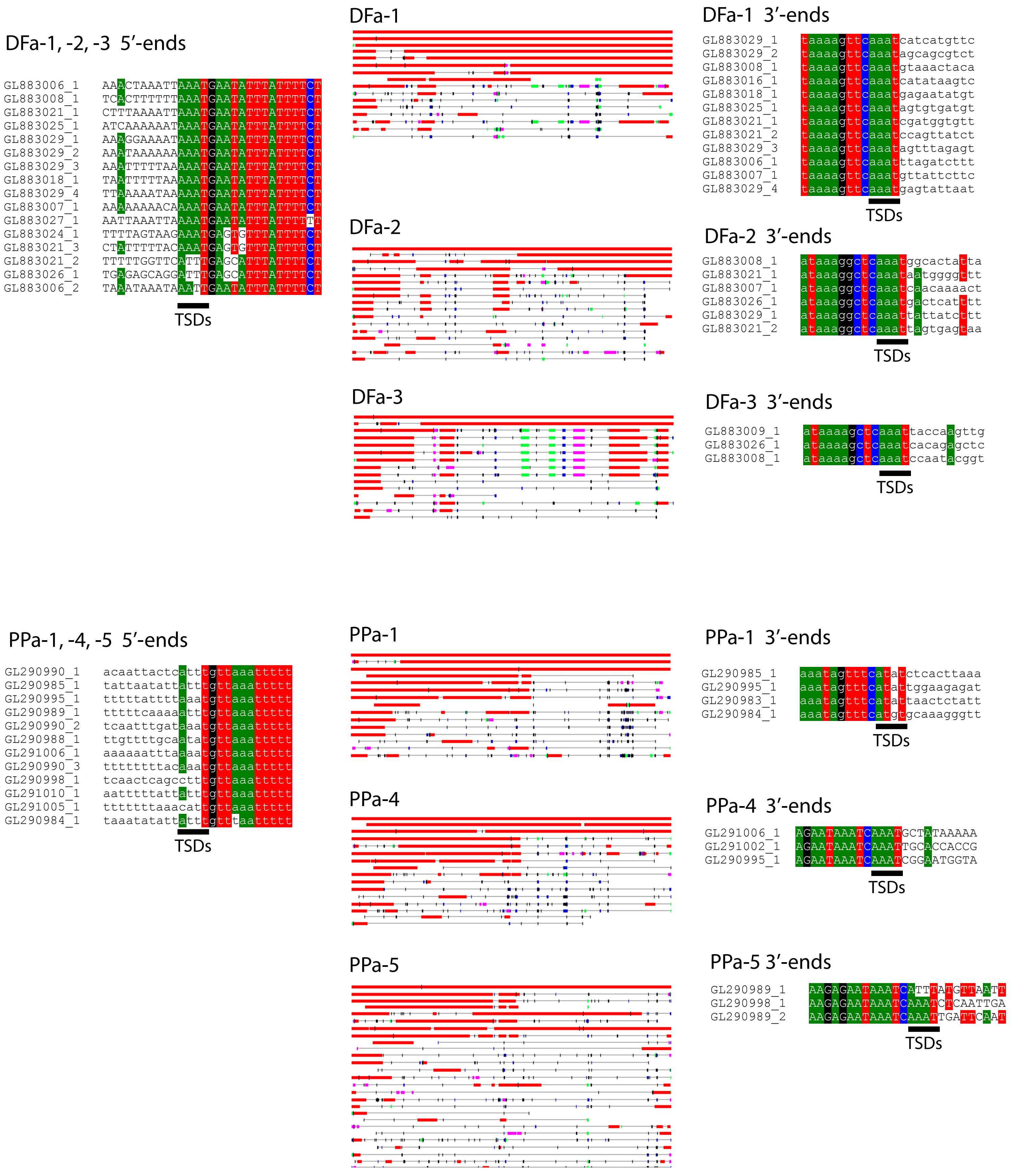

Supplement: Additional file 11 — Alignments of the ends of DFa-1, 2, 3 and PPa-1, 4, 5 families. [file 1759-8753-4-12-S11.pdf]
