# Supplementary figures and images for: Homologues of bacterial TnpB_IS605 are widespread in diverse eukaryotic transposable elements
Source: Mob DNA. 2013 Apr 1;4:12. doi: 10.1186/1759-8753-4-12 (PMC3627910; doi:10.1186/1759-8753-4-12)

Additional file 12

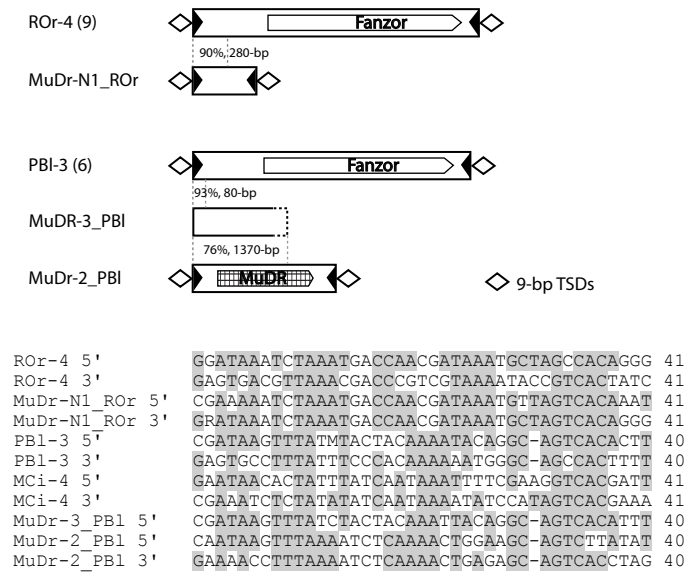

Supplement: Additional file 12 — Fanzor1 protien in MuDr superfamily. [file 1759-8753-4-12-S12.pdf]

Additional file 13

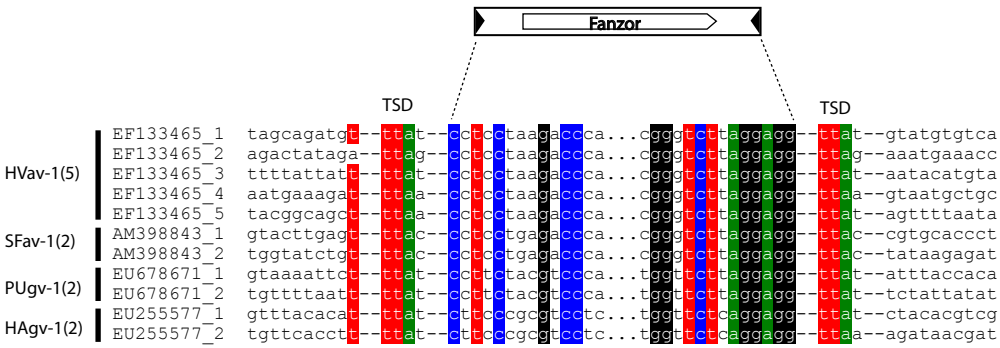

Supplement: Additional file 13 — TSDs of four viral Fanzor1 families. [file 1759-8753-4-12-S13.pdf]
