# Supplementary material for: Homologues of bacterial TnpB_IS605 are widespread in diverse eukaryotic transposable elements
Source: Mob DNA. 2013 Apr 1;4:12. doi: 10.1186/1759-8753-4-12 (PMC3627910; doi:10.1186/1759-8753-4-12)
Supplement: Additional file 14 — Crypton Tpase alignment. [file 1759-8753-4-12-S14.pdf]

Additional file 14

```
MCi-2A           ---------------------------------------------------------------MNAMNGDYAYAQAVEEAAQ-----QTADKAKKVRPKNTTKSYERRKK 42
Crypton-1_MCi    MPRQTLKNKTQAEVDIIVAHENHCDELVEKYIPHFLDIWRNNKRRLQQSENGAPVETVSDTDVPSFLNAEGTYDAALQEEHDRQMQSQAYSDIIKNRSYNTTVTYGYRQI 110
CryptonF-1_CI    -------------------------------------------------------------MATPTLNSQSALMVEMEARVR-----HALDSALDARPKKTRMQYESRQE 44
CryptonF-2_TS    -----------------------------------------------------------MESISNGLLAEQDRQLELHAKIQLHSQ-KVAHRIQKNRPAATTRQYDSRQK 50

MCi-2A           EFLEWCHKH--CPPGALNEIPDDRKLHFFLVTQVLADSQLRD-ELLERAA-----MTSDTV------KRVGLATVKMYIAAVVDLHN-EQSL-ADPHSNRPHPHGKLVDA 136
Crypton-1_MCi    EFLLFCEKYYASMPLADRFQANEKTLLAFMMREIVERRVQKAVESYDEPKATVDISQFESVINNEGGKKLSYSTCNIYVCAVADLWAYQHHVGRNDQ---PSPRTKLITN 217
CryptonF-1_CI    EWRHFCRTK--GFQDG--ELVTEQKLLWFLSECVVGRPAKRSCHSRDRTN-----ENGEPVV-----QTLGQPSIKAYKAAIVNLWSYQRSCRTNPH---PHPVGQAVRA 137
CryptonF-2_TS    EFIDFCTKE--GFPDG--QVVTEKKLVYFLDHYVINRPIRPSRYLRNRTD-----SQGAAVV-----QTLGLPSVKAYTSAIVDLWRFQQSLGTNPY---PNPRGHLVGA 143

MCi-2A           LLQSLKYEEQKHKRDNYVDRGVGTVADGYSTTAEMVKLVDHYFR------------KNTQEGLRNGTAFLLQHYGLLRGESIRMMEFPDLQCMLFEN-EGISTCYALVM- 232
Crypton-1_MCi    ILKIVRQREAKENRDNDVDRAIESVASGYTTVEQVCNISRKLIN-W--------NEQGYQYSFRNSLAFLLSHYLLLRGESIRNLELADLQFMELEKMKSNSTSYPAIIC 318
CryptonF-1_CI    LLETHSRQEHERKKAEFVDRGAGTLLDGYHE-KDIVRLVEYCWQGW----SESKKGQSVEPHLRTAVDFLMGHSMLLRGESRRTAQLADLFTLELTN-EGPTPCFPMVL- 240
CryptonF-2_TS    MIKNHQFDETQRKRTQFTDRGFNTLQDGYTS-ENIRAIVRYCWAGWLSDQTRGRKPQAQEAYLRTTVDFLFGHNMLLRGEDRRHLELADLFTLRMD--EGPTPCWPMIL- 249

MCi-2A           VLKQGKTNQEGRLEFSACLRNKNILICPQMMLSCYLFYRWHIAGEPFPDFNENRDWFDYKLTR---SRNDPKASISYDHHLNCVKEAFKAVGLNSKAK-THAMRGSGSRM 338
Crypton-1_MCi    VFHQGKTNRFNKTESTACIRNREVEACPFMAMALHFFSRYHRYKEPFPSLDCNKDWFKMKVIHVPLAKKHLHAPISKTAHTRFMKYAIQAANIVVKNKLTHMPRICGARM 428
CryptonF-1_CI    IMGNGKTNQMGRIEYATVMRHCNPLLCTMAQTAFYLFYRWDIVREPPPQFHNRQDWYQLHLIK----GDDVRKPLSYETQLDWIRRIYSGTGLSGLKK-THAGRAAGARH 345
CryptonF-2_TS    MKLNGKTNQFGRLEYMGVVRHKDPLLCTICHTAFYLFHXWEIMHEPVPQFYQRQQWYKXVLFK----GSDSEHSFSYETQLKWINQVFQSIGLNSKKK-THSGRSSGARH 354

MCi-2A           AEMFGTSEAAIQRLGRWNNSALTSNYLTHLPREALRTLAGF-TKDAGQFYLVRSTVMPPEELLVKVFPRVDYWLERISKEEVLE-------------------------- 421
Crypton-1_MCi    ASMAGVPEEEVRRQGRWNQDVLTNCYINVLPRQFIRAMAGF--EKDSLYHLPRAMEEPCQALKDMVFPWVDDWKAKIKNGEVKE-------------------------- 510
CryptonF-1_CI    AEQVGVSEGQIRRAGRWNSDALSQCYLTNIPRKFVRAMAGFDSRTPGNFYLPRARVPVPESLERAVWPWVDDWMRWFESYDAQDPNSCLQGPELRRFSDQSGPWDQPRGP 455
CryptonF-2_TS    AELQGVDENSIRRAGHWNQDSMSNCYLSELPRPFIRTLAGFKPTDQGNYYLPRAAIEPPETLVRALWPWIDQWLAWFSPSESNP-------VELSKLDLPPLPLLVQQG- 456

MCi-2A           -------SSTAADGFLNLLVQMRVVFLQDSVILKQKFPDHLLWFDPLFQDELYLQFERY--------------------------------------------------- 473
Crypton-1_MCi    -------QSASASHFLALLTCFKTTLLQDAAVMMDLHPDHPIWKDPIFKTDLFLDFKRKVNSHVAANVIPQSVILTELAPEVQQEITALKTALDE----QHSSVNSRLDA 609
CryptonF-1_CI    SAVRLDRDDLAAQGFLRLLHHLRTVLLQDSVILQPLFPGHPLWTSPVFMREDYRQFAEAVRVANTHKEEPYEMQLQQTVPMVADQLRIVQQDLGVACGHLHTALETGLQK 565
CryptonF-2_TS    -VEKCDQDDLAAQSFLKLLSSFRTVIIQXAVFLQQEFPGHSMWTHPLFQRSDFQSFSQQIIDLVRTSETPHEIKLRQTIPLVANRITTIGENLEHIIQLNHQQTQDSIRA 565

MCi-2A           -------------------------------------------------------------------------------------------------------------- 473
Crypton-1_MCi    TDNGVQQVLS---------LPASSVVNSPFNSPSNPSTNNIPSTSTATTSNIPIPAATTSSASNPRRFQANGVPDFKMSRGLTTVEQLHQEWYTGLGGDWPVAELEATWG 710
CryptonF-1_CI    INNQLEALTSGQVSFLVRAFPAGQRMMTAAAAFEDKDEERDSGSTASREAQAP----ACSLGVAPASASASVPATYQLSRTITTVPDLWQEWTVGLGGGPSVQSLDALYG 671
CryptonF-2_TS    IQSQMDQLFSGEVTFTARLTGSDEKPSTPGNTTSQSTEQNTAHTVVQSLQVTPNPIQDHPPGIHLPNEPSGSPPFYRMSRTIQTVRELWEEWHVGIHGNPSIQSLEDSYG 675

MCi-2A           ---------------------------------------------------------------------------- 473
Crypton-1_MCi    TQWRLNDR-KFVNIRRCVISAISDLENDLGLSTTAAIEQLQDVMARNNWSLHKLGVQLQNGNFYPCEVTKRRKLNQ 785
CryptonF-1_CI    AKWRPGGTERMFYSRRKVIIDYINRQRQGSASGSAAVEELELVRQRGKLSLNRLSRMLRVKKKLP----------- 736
CryptonF-2_TS    CRWRSDNKERVFFSRRKVIIDWIQARVSKGILLADAIDEIELMRRNSQRTLYQLQALLKKGV-------------- 737
```
