# Supplementary material for: Homologues of bacterial TnpB_IS605 are widespread in diverse eukaryotic transposable elements
Source: Mob DNA. 2013 Apr 1;4:12. doi: 10.1186/1759-8753-4-12 (PMC3627910; doi:10.1186/1759-8753-4-12)
Supplement: Additional file 15 — Alignment of Harbinger Tpase. [file 1759-8753-4-12-S15.pdf]

Additional file 15

```
MCi-5              ---MSNLNVSRQLRQRSQRLQSDSHQDNEETNSQANQRRHRTVYKR-GTLEAVLINNANCRYMCRFSIQEIYQITALLGLEENLRFKG-ISVSRQLGFAMLACRYSFPRR 105
Harbinger2-1_HM    ---MIKLSQVRNQLLIFNSDNTINDEELLLLYDVNKSNNLNLSYQSYPRFNLDNMSVDEAKSEFRFLSKDIYEMIDLLNIPEKITCYNGITISADEAFCIFLKRFAYPCR 107
Harbinger2-1_SP    ---MANLHRVQDAVMAAYAFDVIDDVEFILLMDINTSNNLDFQHDG-GHFELDDFNDDECRSYF-FKKDDIFRLKHALGVPDRITLPNRSRMDGLEALCLGLQRYAYPCR 105
Harbinger2-2_NV    ---MSTFRDAREALLLANDLEVIDDDELLLLQNLNKSKNLDIPFEKYDRFDLESLTDDECLSEFRFIKNDLYRLNEALNFPDQITCPNRLTVDGMEALCMTLRRFAYPCR 107
Harbinger2-2_HM    MTALNSLKTARDSLLIAYSEDIIDDCEFALLYQLSYSRDI-YPHWDYNKFNLSLLDDAQCWTDLRFRKTDLPHLLNIFRLPDVIKCTQGTICRGMEALCIMLKRLAFPCR 109
Harbinger2-1_ES    ------MFEAYTDMWMAYNMAFIYTMASLVTDDVATRVAR--------RDVTNFFSPQDCINFLRFTQPQIMLMLDLLLIPAFIRTDCGFAVSGREALCVLLYRLAFPCR 96

MCi-5              YGDMERVFPMHRQNIGKVCKGMEDMVFDKMKYGIQ-FNTHQFREENLKKFAAAIDEAGALIPNVVGFIDGTLQQVSRPATDDDMQKSLYNGWKHVHAIKYQAIVTPDGIT 214
Harbinger2-1_HM    YQDMIPRFSRPVPQLCMISNHIMNLLFAQWGHLLTNLNQGWLDTQHLEMFAAAIHAKGAPLTNCWGFIDGTLRPISRPREHQ---RILYNGHKRCHGIKFQSLVAPNGLI 214
Harbinger2-1_SP    YGDLVKDYRRPVPQLCLAFNWVTNFIFDAHKHRLTTLEQHWLAPQQLRIYADVIHQKGAPLNNCWGFIDGTVRPICRPGEHQ---RVAYNGHKRVHSLKYQSVTSPNGMI 212
Harbinger2-2_NV    YEDLVPRFARAVPQISMVVNEAVSYIYTNYRYLLSSFNQAWLSPAHLEDYARLVALKGAALDNCWGFIDGTVRPICRPGLHQ---RTMYNGHKRVHAIKFQSVVAPNGLI 214
Harbinger2-2_HM    YTDLANTFGRNPTEICLIFNTVIDHVYNKLSHKLLLWDQPMLQSNNLRQFADYIHGKGAPLDNCFGFIDGTVRRIARPKTNQ---RIVYNGHKRVHALKFQSIVVPNGMI 216
Harbinger2-1_ES    LKDMRLVFGLSESCISETFNWMLHFLEFKWGWLLS-LDVERLKPR-LVEFAEAIYNADCPLTHCWGFIDGTVRGIA-PVRFQ---RWYYNGHKRKHAIKFQGVITPDGLF 200

MCi-5              SSMMGPVIGARHDKFMYTMLQTEKRLQKYLHISDREKDNYAIYGDPAYEESEHLHCPLDKTFPSQSTDPLEIECNKSMAKVRIAVEWEFAEVMKYFSYCKYKYAMKVDSN 324
Harbinger2-1_HM    ANLYGPVEGKRHDSGMLADSNLLNKLAV--CSFNSNREPLCVYGDPAYPHRVNLQCGFKGAN----ISPEQLIWNKNMSKVRVAVEWVFGDIVNYFKFLDFKKNLKVGLS 318
Harbinger2-1_SP    ANLYGPVEGRRHDAYLLRESGLLTMLEG--GSHDAEGNLLCIYGDPAYPVRPQLQAPFPTAN----ITPEQAAFNRAMSKVRITVEWSFGDVINFFKYTDFKKSQKILLS 316
Harbinger2-2_NV    ANLFGPIEGKRHDSAMLHESGLLPLLER--HAINTAGQPLCLYGDPAYPLRAQLQCPFRGRN----LQAQQELFNLSMSKVRTAVEWVFNDISTYFAFLDFKKDLKIGLS 318
Harbinger2-2_HM    ANLAGPFEGKKHDSTMLCESGLLQQLQQ--FAWHD-GRPLCLYGDPAYPIGVHLLAPYRSLN----ITPDQHAFNKAMSAHRVSVEWVFGLMTNYFKFIDFKQSQKLGSS 319
Harbinger2-1_ES    VDMWGPVLGTRHDSYLLAQSGLMQKLAT--HSNSPSGHPYCLYGDPAYGLSDHLACPFSASSHG-PLTPDMADFNQRMSHCRVAVEWGFKEMTSKWTFVDMKPQQKYLLS 307

MCi-5              NPAKVYILSTIFKNMLHCAYQRGYSTFAKFKVNPPTLEDYIQGMRREKIEGEDEDM 380
Harbinger2-1_HM    PVSKMYLVCALMHNARVCLYG--STTTTYFDCQPPSLSDYFK-------------- 358
Harbinger2-1_SP    SCAKMYMVSAILTNAHTCIYG--NNTSSYFELQPPSLDEYFQ-------------- 356
Harbinger2-2_NV    PVGKMYIVCALLRNANTCLYG--SSTSQFFGLDPPDLEVYFT-------------- 358
Harbinger2-2_HM    PIGKVYIVCSLIQNAHTCLYG--NIVSDYFGLEAPSLQEYFQ-------------- 359
Harbinger2-1_ES    PVGKQYLVATLLSNIHSCLNGG-NQISQFFDVQPPTLQEYLKV------------- 349
```
